# Water-soluble extracellular vesicle probes based on conjugated oligoelectrolytes

**DOI:** 10.1126/sciadv.ade2996

**Published:** 2023-01-11

**Authors:** Cheng Zhou, Sarah J. Cox-Vázquez, Geraldine W. N. Chia, Ricardo Javier Vázquez, Hui Ying Lai, Samuel J. W. Chan, Jakkarin Limwongyut, Guillermo C. Bazan

**Affiliations:** ^1^ Department of Chemistry, National University of Singapore, Singapore 117543, Singapore.; ^2^ Institute of Polymer Optoelectronic Materials and Devices, State Key Laboratory of Luminescent Materials and Devices, South China University of Technology, Guangzhou 510640, PR China.; ^3^ Institute for Functional Intelligent Materials, National University of Singapore, Singapore 117544, Singapore.; ^4^ Department of Chemistry and Biochemistry, Center for Polymers and Organic Solids, University of California, Santa Barbara, Santa Barbara, CA 93106, USA.; ^5^ Department of Chemical and Biomolecular Engineering, National University of Singapore, Singapore 117585, Singapore.

## Abstract

We developed a series of transmembrane conjugated oligoelectrolytes (COEs) with tunable optical emissions from the UV to the near IR to address the false-positive problem when detecting nanometer-sized extracellular vesicles (EVs) by flow cytometry. The amphiphilic molecular framework of COEs is defined by a linear conjugated structure and cationic charged groups at each terminal site. Consequently, COEs have excellent water solubility and the absence of nanoaggregates at concentrations up to 50 μM, and unbound COE dyes can be readily removed through ultrafiltration. These properties enable unambiguous and simple detection of COE-labeled small EVs using flow cytometry with negligible background signals. We also demonstrated the time-lapsed tracking of small EV uptake into mammalian cells and the endogenous small EV labeling using COEs. Briefly, COEs provide a class of membrane-targeting dyes that behave as biomimetics of the lipid bilayer and a general and practical labeling strategy for nanosized EVs.

## Teaser

A class of lipophilic probes addresses the challenge of accurate and straightforward detection of extracellular vesicles.

## INTRODUCTION

**Fig. 1.**
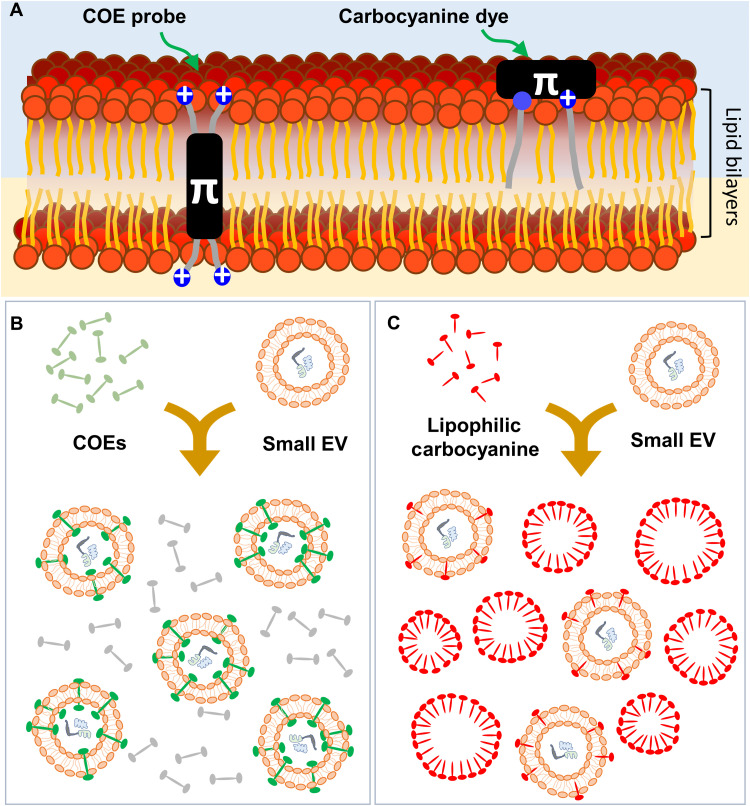
Schematic diagrams of small EV dye design principles. (**A**) Membrane intercalation of COE and carbocyanine probes. (**B**) Small EV labeling using COEs and the corresponding fluorescence “turn-on” mechanism. (**C**) Small EV labeling using lipophilic carbocyanine dyes and the “false-positive” signals due to micelle formation.

**Fig. 2.**
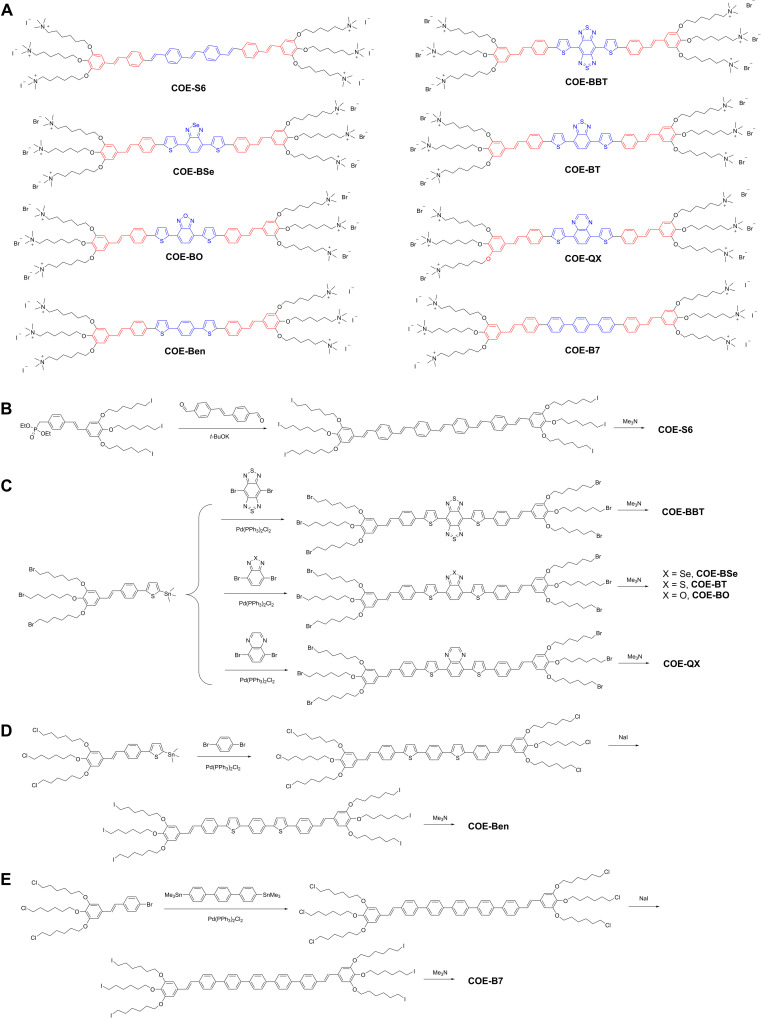
Chemical structure and synthesis. (**A**) Chemical structures of COEs in this study. (**B** to **E**) Their synthesis routes.

Conjugated oligoelectrolytes (COEs) are a class of molecules comprising a hydrophobic conjugated backbone decorated by pendant ionic functional groups (*1*–*3*). Membrane-intercalating COEs are based on a linear topology and mimic the general spatial distribution of hydrophilic and hydrophobic domains of lipid bilayers (Fig. 1A). As a result of electrostatic and hydrophobic interactions, these linear COEs intercalate into lipid membranes with a concomitant fluorescence enhancement (*4*). When the molecular lengths are similar to the thickness of the lipid bilayers, COEs exhibit biocompatibility and stable binding (*5*). These general features have led to observations that COEs can be used as labeling dyes for yeast, bacteria, and mammalian cells (*1*, *6*, *7*). Other functions have also been reported. For example, **COE-S6** (Fig. 2A), containing an oligophenylenevinylene backbone, was used for selective in situ staining of Gram-positive bacteria in a three-dimensional polymicrobial biofilm (*4*). The narrow optical bandgap **COE-BBT**, which contains a donor-acceptor-donor configuration in the conjugated segment, was used as a long-term in vivo NIR-II fluorescent tracker to monitor the progression of the subcutaneous tumor over 26 days (*8*). The unique structural features of COEs and their straightforward synthesis open opportunities for designing advanced optical probes tailored for specific applications.

Extracellular vesicles (EVs) are membrane-confined particles that comprise endosome-origin “exosomes” and plasma membrane–derived “ectosomes” (*9*, *10*). Exosomes (called small EVs after this paragraph) are typically ~30 to 140 nm in size and contain proteins and genetic materials from the cells they originate (*11*). They are involved in cell-to-cell communication and the exchange of genetic information, which have downstream implications on cellular functions and activities (*12*). Moreover, the onset and progression of certain diseases (e.g., cardiovascular inflammation, neurodegeneration, and cancer) are associated with exosome biogenesis (*13*, *14*). Thus, exosomes hold potential as biomarkers and are promising targets for liquid biopsy assays (*15*). Exosomes also mediate biological responses, implying that they can be engineered as therapeutic vehicles for drug delivery (*16*). For example, somatic exosomes from the blood of healthy individuals are being used to deliver therapeutic drugs because of the advantages of specificity, safety, and stability (*17*, *18*). The multifaceted potential of exosomes has led to vigorous research activities (*19*).

A common challenge arises from the difficulty in detecting and characterizing small EVs (*20*–*22*). Methods for their detection include electron microscopy, flow cytometry, nanoparticle tracking analysis, dynamic light scattering (DLS), atomic force microscopy, and resistive pulse sensing (*23*, *24*). Flow cytometry is most commonly reported among these techniques, given its ability to generate rapid and high-throughput multiparameter analysis (*25*, *26*). Improvements in resolution have been achieved using dedicated high-sensitivity flow cytometry (*27*, *28*). These capabilities rely on the availability of dyes designed explicitly for small EV labeling and provide sufficient optical output from nanoscale particles.

Three general classes of fluorescent probes are commonly reported for small EV detection by flow cytometry: antibody dyes, dyes that bioconjugate to intravesicular components, and membrane-targeting lipophilic dyes (*21*). Antibody-based dyes rely on specific interactions with small EV surface markers, such as tetraspanins CD9, CD63, and CD81. Intracellular dyes such as carboxyfluorescein succinimidyl ester penetrate the small EV and covalently bind with amine groups in the intracellular proteins (*29*). The antigen expression and intracellular protein content depend on the cellular origin, which may be heterogeneous within a small EV population. Therefore, for population analysis, it is essential to use a universal optical reporter that labels the vast majority of small EVs unbiasedly to provide a normalization basis for comparison across different samples. The lipid bilayer, being a hallmark component of all small EVs, would make a reasonable target for this purpose.

Lipophilic carbocyanine–based membrane probes with a phospholipid-like structure that contains a fluorescent polar head group and two hydrocarbon tails have been widely used to label small EVs. Prominent examples include the Di (**DiO**, **DiI**, **DiD**, and **DiR**) and PKH (**PKH-67** and **PKH-26**) families of probes (fig. S1) (*30*, *31*). Their molecular structures include relatively long hydrocarbon chains that increase hydrophobicity and enable more stable intercalation into lipid membranes than the similar dyes with shorter carbon chains (*32*). However, long hydrocarbon chains may limit solubility in aqueous media. As a result, it is known that these lipophilic carbocyanine dyes in aqueous media aggregate into particles with sizes similar to small EVs, a situation that can lead to the generation of false-positive signals in flow cytometry (Fig. 1C) (*33*). These particles may be removed through additional purification steps (such as ultracentrifugation or size exclusion chromatography) at the expense of low recovery yields (*34*). Lipophilic dyes, such as MemBright and Mem family, have been recently developed with the intention of mitigating aggregation by increasing the hydrophilicity in the molecular framework of cyanine dyes (*35*, *36*). However, these modifications may compromise the membrane binding efficiency due to reducing the hydrophobic content. Therefore, groundbreaking approaches in dye design specific to small EV detection are of interest to address these unsolved problems.

We disclose here the application of COEs as effective optical labels for small EVs from the ultraviolet (UV) to the near-infrared (NIR) regions. The optical fine-tuning is achieved by judiciously tuning the π-conjugated molecular structure of the COE (Fig. 2A). Our design used six positively charged side chains to achieve solubility in water greater than 50 mg ml^−1^. Biophysical experiments using liposome models show that COEs are fluorogenic membrane-targeting probes, a feature that helps mitigate fluorescence background from unbound dyes (*37*). Ultrafiltration and light scattering experiments indicate that these COEs do not form aggregates in aqueous media under conditions useful for labeling small EVs for flow cytometry. It is also possible to endogenously label small EVs by adding COEs to parent cells. Last, we show that the time-lapsed uptake of COE-labeled small EVs could be tracked within mammalian cells through single-particle analysis. These results highlight unique opportunities that the COE chemical framework provides for developing dyes suitable for small EV analysis.

## RESULTS

### Design, synthesis, and characterization of COEs

Eight COEs were examined, in which their conjugated backbones share a stilbene “wing” (marked in red) but differ in the “core” segments (marked in blue); see Fig. 2A. Although **COE-S6** and **COE-BBT** were previously reported, we discuss their preparation with those other COEs to generate a context for differences in synthetic strategies (*5*, *8*). The backbone of **COE-S6** was synthesized through the Horner-Wadsworth-Emmons reaction between the stilbene phosphonate wing and the stilbene aldehyde core, followed by a quaternization reaction to obtain the final product (Fig. 2B). The backbone of **COE-BBT** was constructed via a Stille coupling reaction between the benzobisthiadiazole core and the stilbenethiophenyl wing (Fig. 2C). To obtain a range of optical profiles between those of **COE-S6** and **COE-BBT**, we used a design strategy based on modulating donor-acceptor-donor units in the core segment, where the strong acceptor benzobisthiadiazole in **COE-BBT** was replaced with gradually weaker electron-deficient units, such as benzoselenadiazole (**COE-BSe**), benzothiadiazole (**COE-BT**), benzooxadiazole (**COE-BO**), quinoxaline (**COE-QX**), and phenylene (**COE-Ben**). Compounds **COE-BT**, **COE-BSe**, **COE-QX**, and **COE-BO** share the same synthetic route as that of **COE-BBT** while using different aryl halides in the Stille coupling reaction (Fig. 2C). The preparation of **COE-Ben** requires one to use a different reactant in the step that organizes the aromatic framework, specifically a stannylated styrylphenyl thiophene with chloro-hexyl side chains in the terminal aromatic unit. This requirement is needed to avoid the generation of by-products, given that dibromobenzene has lower reactivity in the Stille coupling reaction relative to its more electron-deficient counterparts (*38*). Halogen exchange reaction from chloride to iodide was performed before the final quaternization reaction that yields **COE-Ben** (Fig. 2D). Detailed synthesis procedures and structural characterizations for all COEs and intermediates are documented in the Supplementary Materials.

**Fig. 3.**
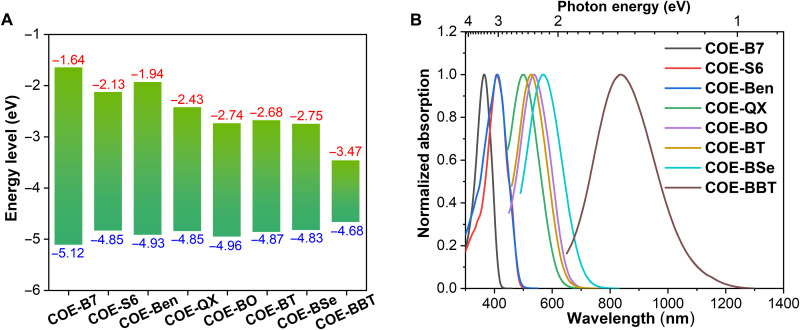
Simulated and optical bandgaps of COEs. (**A**) DFT-calculated HOMO (blue) and LUMO (red) energy levels using the B3LYP/6-31G(d,p) functional and basis set. (**B**) Normalized absorption spectra of each COE in PBS.

The optimized backbone conformations and energy levels of the highest occupied molecular orbitals (HOMOs) and lowest unoccupied molecular orbitals (LUMOs) of all COEs were calculated using density functional theory (Fig. 3A and figs. S2 and S3) (*39*). Calculated HOMO/LUMO energy gaps match the experimental observations of the absorption edges in phosphate-buffered saline (PBS; Fig. 3B). The optical bandgaps increase as the core segments are substituted with more electron-rich donor moieties. Consequently, there is a gradual blue-shift in the absorption spectra from **COE-BBT** to **COE-Ben**. Notably, **COE-Ben** has the same optical bandgap of 2.6 eV as **COE-S6** (Table 1).

**Table 1.** Chemical and physical properties of COEs. N.D., not determined.

COE-compound	B7	S6	Ben	QX	BO	BT	BSe	BBT
Molecular weight (g mol^−1^)	2298	2300	2310	2080	2070	2086	2133	2144
Simulated bandgap (eV)	3.5	2.7	3.0	2.4	2.2	2.2	2.1	1.2
Optical bandgap (eV)	3.0	2.6	2.6	2.1	2.0	2.0	1.9	1.2
λ_abs, in PBS_ (nm)	365	408	410	500	536	527	569	836
ε (×10^4^ M^−1^ cm^−1^)	9.8	11	7.0	2.6	3.0	2.6	2.2	1.9
λ_abs, with SUVs_ (nm)	362	415	415	499	525	524	563	859
λ_em, with SUVs_ (nm)	434	467	492	609	601	614	674	1017
Stokes shift in SUVs (nm)	72	52	77	110	76	90	111	158
Stokes shift in SUVs (eV)	0.57	0.33	0.47	0.45	0.30	0.35	0.36	0.22
PL fold increase in SUVs	4.9	8.2	13	11	220	56	190	N.D.
Quantum yield in SUVs (%)	94	70	60	23	27	25	9.2	0.8
PL lifetime in SUVs (ns)	1.1	0.9	0.9	2.4	1.9	2.6	2.7	0.34
IC_50_ against A549 cells (μM)	>256	>256	>256	120	170	>256	>256	250

**COE-B7** was designed and synthesized to achieve blue-shifted absorption relative to **COE-S6**. The core of **COE-B7**, which relies on a pentaphenylene sequence, instead of oligophenylinevinylene, was synthesized via the Stille coupling reaction between a terphenyl core and the stilbenyl wing, followed by halogen exchange and quaternization reactions (Fig. 2E). The optical bandgap in PBS solution was determined to be 3.0 eV by the absorption spectrum, as a result of the increased dihedral angle that reduces the intermolecular charge transfer (fig. S2) (*40*). With **COE-B7**, it is possible to complete a set of COEs with absorption spectra that range from the UV to the NIR.

### COEs as membrane-targeting fluorogenic probes

**Fig. 4.**
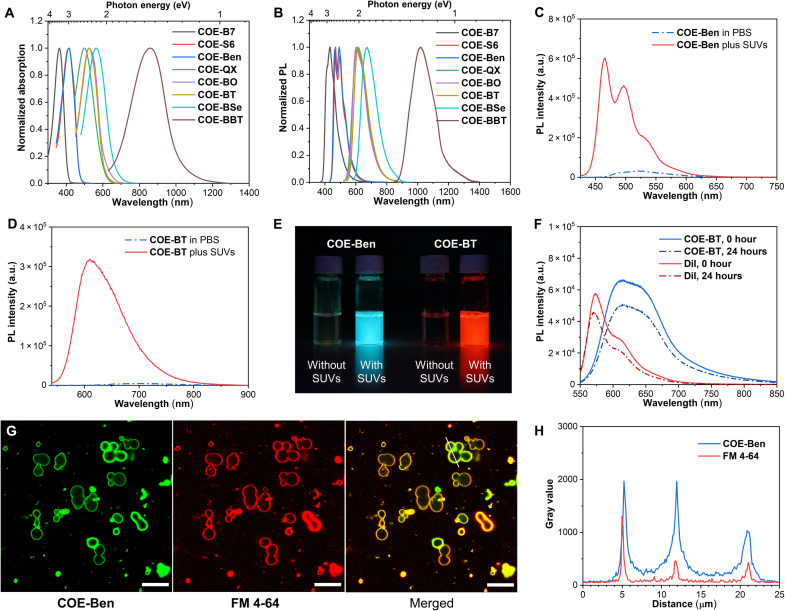
Properties of COEs as membrane-targeting fluorogenic probes. (**A**) Normalized absorption spectra of 20 μM COEs after adding 1 mM SUVs in PBS. (**B**) Normalized PL spectra of each COE after adding SUVs (1 mg ml^−1^) in PBS. (**C** and **D**) PL spectra of 1 μM **COE-Ben** and 2 μM **COE-BT** in PBS without or with the addition of 1 mM SUVs. a.u., arbitrary units. (**E**) Photograph of 0.5 μM **COE-Ben** and 1 μM **COE-BT** in PBS with or without 1 mM SUV treatment under exposure using a handheld UV 365-nm lamp. (**F**) Photostability evaluation of **COE-BT** with SUV addition under continuous exposure of a 5-mW 532-nm laser over 24 hours. (**G**) Confocal micrographs of LMVs (6.25 mg ml^−1^) after staining with 15 μM **COE-Ben** and 15 μM **FM 4-64** in PBS for 30 min at room temperature. The mixture was diluted five times using PBS before confocal imaging. The **COE-Ben** channel was observed by excitation at 405 nm and collecting the emission in the range of 450 to 490 nm (represented in green), and the **FM 4-64** channel was observed by excitation at 561 nm and collecting the emission in the range of 640 to 700 nm (represented in red). Scale bars, 20 μm. (**H**) Gray value curves represent the fluorescence intensity profile of the white line in its left-side fluorescent micrograph for both channels.

Optical absorption and photoluminescence (PL) spectra were measured after the individual COEs were added to solutions of small unilamellar vesicles (SUVs; Fig. 4, A and B). These SUVs contained 1-palmitoyl-2-oleoyl-glycero-3-phosphocholine (POPC) and 1-palmitoyl-2-oleoyl-*sn*-glycero-3-phospho-(1′-rac-glycerol) sodium (POPG) in a molar ratio of 85:15 and were extruded using a 100-nm membrane. These SUVs were chosen to mimic mammalian cell and small EV membranes (*41*). No substantial differences were observed in the spectral shapes among the absorption spectra measured in neat PBS (Fig. 3B), absorption spectra measured in the presence of 1 mM SUVs (Fig. 4A), and excitation spectra in the SUV solutions (fig. S4) for all COEs studied in this work.

We first examined the binding of COEs to SUVs. A 5 mM SUV solution was treated with 50 μM **COE-BT** (fig. S5). Passing the resulting suspension through a 100,000 Da molecular weight cutoff (MWCO) ultrafiltration membrane, with a mean pore size of approximately 11 nm (*42*), led to the exclusive recovery of COE-labeled SUVs. No **COE-BT** could be detected in the filtrate via absorbance measurements. Similar results were observed for the other COEs (fig. S5).

Insertion into SUVs was also evident from an increase in the zeta potential of POPC-only SUVs with increasing concentrations of the positively charged COEs. For example, the SUV zeta potentials changed gradually from approximately 0 to 40 mV when the concentrations of **COE-BT** increased from 0 to 10 μM (fig. S6). Similar changes in the zeta potentials were observed for the POPC SUVs treated with any of the other seven COEs (fig. S7A).

More intense emissions from the COEs were observed in the presence of SUVs (see Table 1). For example, the PL intensity increased by 13- and 56-fold for **COE-Ben** and **COE-BT**, respectively, when comparing the integrated emission area in PBS against that observed in the presence of SUVs (Fig. 4, C and D, and see fig. S8 for other COEs). This feature can be easily appreciated by the emission captured on a Nikon camera when **COE-Ben** or **COE-BT** was illuminated under a 365-nm UV lamp with or without SUV addition (Fig. 4E and see fig. S9 for other COEs). We attributed the increase in PL intensity to the insertion of COEs into the SUV lipid bilayers, wherein emitter sites are in a less polar environment with less contact with water molecules (*43*). **COE-BO** exhibited the largest increase in PL intensity (220-fold), while **COE-B7** exhibited the smallest emission increment (4.9-fold; fig. S8). It is likely that **COE-B7** is less sensitive to polarity changes due to the less pronounced contribution from intramolecular charge-transfer excited states (*44*). In the case of **COE-BBT**, which has the narrowest optical gap and its emission in PBS is below the detection limits of our instrument (fig. S10), the fold increase in fluorescence intensity could not be determined.

PL quantum yields (PLQYs) for COEs in the presence of SUVs were calculated using a relative method (fig. S11). For instance, a PLQY of 94 ± 3% was observed for **COE-B7** relative to quinine sulfate (PLQY = 58%, in 0.1 M H_2_SO_4_ aqueous solution) (*45*). The PLQY of the NIR emitting **COE-BBT** was 0.8 ± 0.1% with respect to IR-26 (0.05%, in 1,2-dichloroethane) (*46*). The PLQY is generally correlated to the emission wavelength, where COEs with the more red-shifted emission exhibit a lower PLQY, as would be naturally expected on the basis of the energy gap law (*47*). The PL lifetimes for the COEs in SUVs ranged from 0.34 to 2.7 ns (figs. S12 and S13).

Photostability after membrane intercalation was evaluated using **COE-BT** as a representative example. Carbocyanine dye **DiI** (1,1′-dioctadecyl-3,3,3′,3′-tetramethylindocarbocyanine perchlorate) (λ_em_ = 574 nm), with a close emission wavelength to **COE-BT** (λ_em_ = 614 nm), was used as a comparator because of its reported outstanding photostability (*48*). **COE-BT** (5 μM) and **DiI** (2.87 μM) were added to a suspension of SUVs to achieve a similar absorbance at 532 nm (fig. S14). After 24 hours of continuous exposure using a 5-mW 532-nm laser source, the integrated areas under the emission curves are 75% for **COE-BT** and 78% for **DiI** relative to the initial SUV-containing suspensions, suggesting similar photostabilities for the two emitters (Fig. 4F). Excellent photostability was also reported for **COE-BBT** after membrane intercalation (*8*).

To support that COEs are membrane-targeting fluorophores, large multilamellar vesicles (LMVs) with several micrometer sizes were prepared. A commercially available lipophilic probe **FM 4-64** (PLQY = 4% in dichloromethane) was selected as the reference (Fig. 4G and fig. S15) (*4*, *49*). For optical compatibility with red-emitting **FM 4-64**, the green-emitting **COE-Ben** was used in this study, so that both dyes could be excited and observed separately by fluorescence microscopy in the same sample. Membrane intercalation competition of the two positive-charged dyes and heterogeneity of LMVs caused the intensity differences for the two fluorescent channels (*4*). Although the LMVs were costained by both dyes, the overlay of micrographs from the two channels indicates colocalization of **COE-Ben** and **FM 4-64**, as determined by the coincidence of green and red fluorescence traces in Fig. 4H. Further microscopy studies validated the colocalization of COE and **FM 4-64** in mammalian cell membranes (fig. S16). Membrane-targeting labeling micrographs have also been observed for other COEs (*4*, *5*, *8*). In tandem with these data, all COEs were observed to exhibit a blue-shift in their emission spectra in the presence of SUVs (fig. S10), which further supports the incorporation from an aqueous polar environment into the more hydrophobic lipid bilayer. These results are consistent with the function of COEs as fluorogenic membrane-targeting probes.

### Absence of COE aggregation in aqueous media

**Fig. 5.**
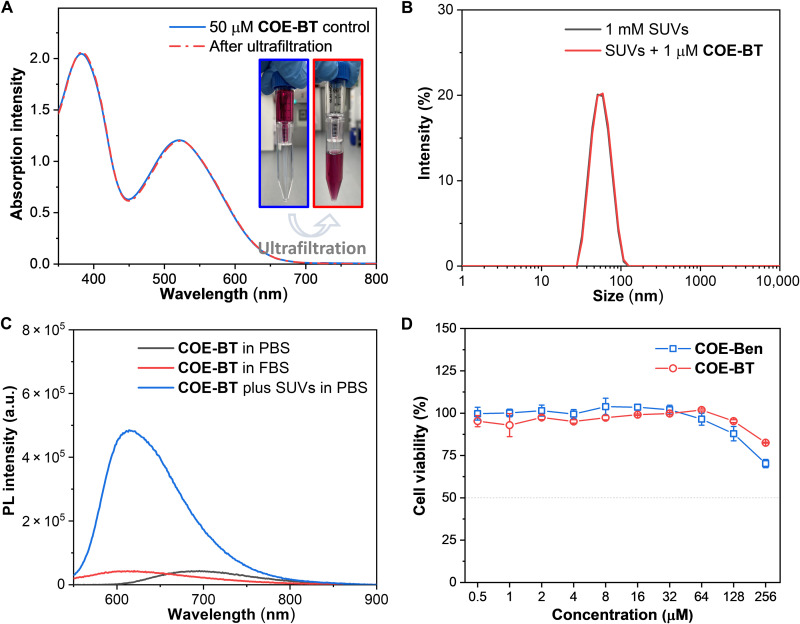
Properties of COEs as potential small EV probes. (**A**) Absorption spectra and photographs of 50 μM **COE-BT** before and after ultrafiltration using 100,000 MWCO protein concentrator tubes. (**B**) DLS measured size distribution curves by the intensity of 1 mM SUVs without or with 1 μM **COE-BT** treatment in PBS. (**C**) PL spectra of 2 μM **COE-BT** in PBS or FBS or with SUV treatment (1 mg ml^−1^) in PBS. (**D**) Cytotoxicity measurements against the A549 cell line.

We examined the possibility of COE aggregation. First, we passed 50 μM **COE-BT** in PBS through the same ultrafiltration tubes used to test for SUVs’ binding. More than 99% of the COE passed through the membrane filter (Fig. 5A), as determined by absorption spectroscopy. The other COEs in this study showed similar behaviors (fig. S17). Furthermore, DLS measurements of 1 μM **COE-BT** in PBS recorded a weak correlation coefficient curve and low derived mean count rate (fig. S18, A and B). The inability to generate a reliable size distribution curve for **COE-BT** by DLS is consistent with the ultrafiltration experiment results, namely, there is no detectable aggregation at the concentrations tested. Similar observations were made for other COEs under the same test conditions (fig. S18, C and D). The Tyndall effect’s absence also supported that these COEs will not form nanoaggregates in an aqueous solution (fig. S19).

### Vesicle morphology after labeling

The binding of an optical reporter to small EVs should not modify their physiochemical properties substantially. It has been reported that carbocyanine dyes, such as **PKH-26**, induce a change in small EV size after labeling (*36*, *50*). We thus investigated whether the same phenomenon would be observed with COEs by using SUVs as a model for the small EVs. SUVs, prepared by extrusion through a 30-nm membrane, were characterized by DLS to have a *Z*-average of 53.6 ± 0.5 nm and a polydispersity index (PDI) of 0.06 ± 0.02 (Fig. 5B). We observed that the addition of 1 μM **COE-BT** into 1 mM SUVs did not result in a notable change in the size distribution curves, wherein similar *Z*-average and PDI values (53.5 ± 0.6 nm and 0.05 ± 0.01, respectively) were measured. Even with a higher **COE-BT** concentration of 10 μM, the size of the COE-labeled SUVs remained unchanged (54.0 ± 0.5 nm for *Z*-average and 0.06 ± 0.03 for PDI; fig. S20). We further observed that the other seven COEs did not induce a change in SUV size (fig. S7B). SUVs keep an intact morphology and similar size was further visualized using cryogenic transmission electron microscopy (cryo-TEM; fig. S21). The COE binding has a minor effect on small EV morphology, as confirmed by nano–flow cytometry scattering (fig. S22) and electron microscopy (fig. S23) experiments.

### Selectivity in the presence of proteins

We examined whether COEs are fluorogenic in the presence of proteins instead of lipids by measuring their emission in fetal bovine serum (FBS). As shown in Fig. 5C, **COE-BT** in FBS exhibited a slight blue-shift in emission, relative to **COE-BT** in PBS. However, although FBS contains a high number of EVs, the emission intensities of **COE-BT** in FBS are ~9 times lower in comparison to that of **COE-BT** in the presence of SUVs (see fig. S24 for other COEs). We repeated the ultrafiltration experiments for **COE-BT** in FBS, in which a substantial fraction of **COE-BT** was found to be associated with the proteins, or other components, in FBS (fig. S25). The high association to proteins and negligible fluorescence increase were further validated when FBS was replaced by bovine serum albumin (fig. S26). These results indicate that COEs may bind proteins, but their emission intensities are not substantially increased, as compared to their binding into lipid membranes.

### Biocompatibility

Cytotoxicity measurements for the COEs were carried out using A549 cells (Fig. 5D and fig. S27A). These studies showed that the half-maximal inhibitory concentration (IC_50_) values were generally higher than 100 μM. The lowest IC_50_ value among the COEs was 120 μM for **COE-QX**. We attribute this higher cytotoxicity to the presence of the bioactive quinoxaline moiety in its core unit (*51*). **COE-B7**, **COE-S6**, and **COE-Ben** were selected as representative COEs to investigate hemolysis because their absorption profiles do not interfere with the absorption of lysed erythrocytes at 540 nm. Notably, all three COEs only exhibited <5% hemolysis, even at the highest tested concentration of 250 μM (fig. S27B).

### Small EV labeling using COEs

**Fig. 6.**
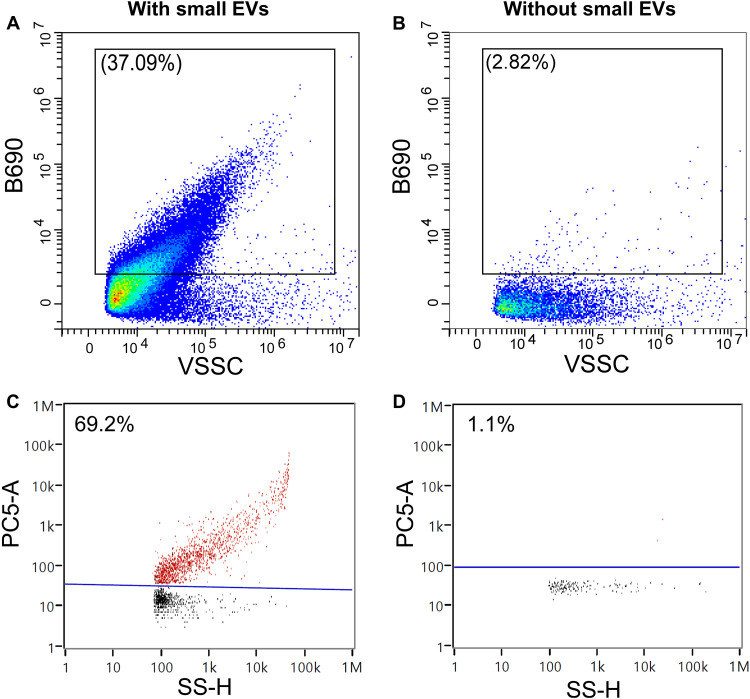
Small EV labeling using flow cytometry. (**A** and **B**) Flow cytometry data were collected on a CytoFLEX LX (Beckman Coulter) based on PC-3 small EVs stained by 1 μM **COE-BT** in PBS. (**C** and **D**) Flow cytometry data were collected on a NanoAnalyzer (NanoFCM) based on SW480 small EVs stained by 1 μM **COE-BT** in PBS. The same concentration of **COE-BT** in PBS was used as the negative control. The small EV mixture and controls were diluted 100 times using PBS before measurements. The excitation and emission wavelengths are 488 and 690 nm for the B690 channel, 561 and 585 nm for the Y585 channel, and 488 and 670 nm for the PC5 channel, respectively. The size was detected by either VSSC or SS channel.

The absence of COE nanoaggregates prompted our interest in their application to label small EVs. We first examined whether COE-labeled small EVs could be analyzed using a conventional flow cytometer (CytoFLEX LX, Beckman Coulter). To increase the sensitivity for small-particle detection, the blue side scatter (SSC; 488 nm) channel configuration was first switched to the violet side scatter (VSSC; 405 nm), since the shorter-wavelength laser provides more orthogonal light scattering (*52*). Commercially available small EVs derived from the PC-3 human cancer cell line were purchased from Abcam and used as test samples, whose quality was verified using multiple techniques, including nano–flow cytometry scattering, electron microscopy, and EV surface markers (figs. S28, S23A, and S29). Dye-positive events were gated relative to the dot plots of the unlabeled small EV sample on the appropriate channels (fig. S30). The same gating strategy was used throughout all the measurements. The sample containing small EV (approximately 10 μg ml^−1^) and 1 μM **COE-BT** in PBS was observed to achieve 37% positive events (Fig. 6A). Under the same conditions, but in the absence of small EVs, a lower percentage of signals (2.8%) could be observed at the same concentration of **COE-BT** in PBS (Fig. 6B). Hence, we can assign all the positive events in Fig. 6A to be signals from the **COE-BT**–labeled small EVs given the low rate of false positives (i.e., absence of dye-only artifacts). The same small EV analysis was carried out with the six other COEs here, and the same certainty in accurately detecting COE-labeled small EVs was observed (fig. S31). Among them, 0.5 μM **COE-Ben** achieved the highest positive percentage of 76%. Other EVs secreted from different cell lines or isolated using different methods were added as testing samples, and COE retains a high performance (fig. S32). The small EVs can simultaneously be costained by COE and antibody markers (fig. S33), indicating that COEs exhibit less impact on EV physiology and highlighting theirversatile capabilities.

We next examined the applicability of COEs for small EVs using a dedicated nano–flow cytometer (NanoAnalyzer, NanoFCM) that contains a reconstructed scattered light system with specialized data processing strategy for high-resolution and distinct nanoparticle detection (*53*, *54*). Dye-positive events were gated using a linear line in the gap between the two distinct populations. After staining with 1 μM **COE-BT** and using excitation at 488 nm, 69% positive events were observed for small EVs derived from SW480 cells (Fig. 6C). Under the same test conditions, the sample containing only **COE-BT** exhibited negligible background (1%) in the positive subpopulation with much fewer events in the negative subpopulation (Fig. 6D). Similar high EV staining ratio and negligible background were also observed for **COE-BO**, **COE-QX**, and **COE-BSe** (fig. S34).

On the basis of the information presented above, we provide in Fig. 1B a schematic representation summarizing the use of COEs to label small EVs. COEs have excellent solubility and resist the formation of nanoaggregates in aqueous solution, at least up to concentrations of 50 μM. After incorporation into small EV membranes, the fluorescence of COEs increases substantially. Unbound COE is not detected by scattering in flow cytometry. Therefore, COE-labeled small EVs provide distinct signals with low background.

We selected **PKH-26** as a reference carbocyanine dye for comparison in both flow cytometers. In the **PKH-26**–only controls, serious “positive events” were observed, compromising the interpretation of data obtained for the **PKH-26**–labeled small EVs despite reporting a high percentage of positive events (fig. S35). The false positives are likely to arise from detecting **PKH-26** dye particles that are self-assembled into micelles upon exposure to an aqueous buffer environment, as confirmed by the Tyndall effect and DLS measurements (fig. S36).

### Small EV tracking by using COEs

**Fig. 7.**
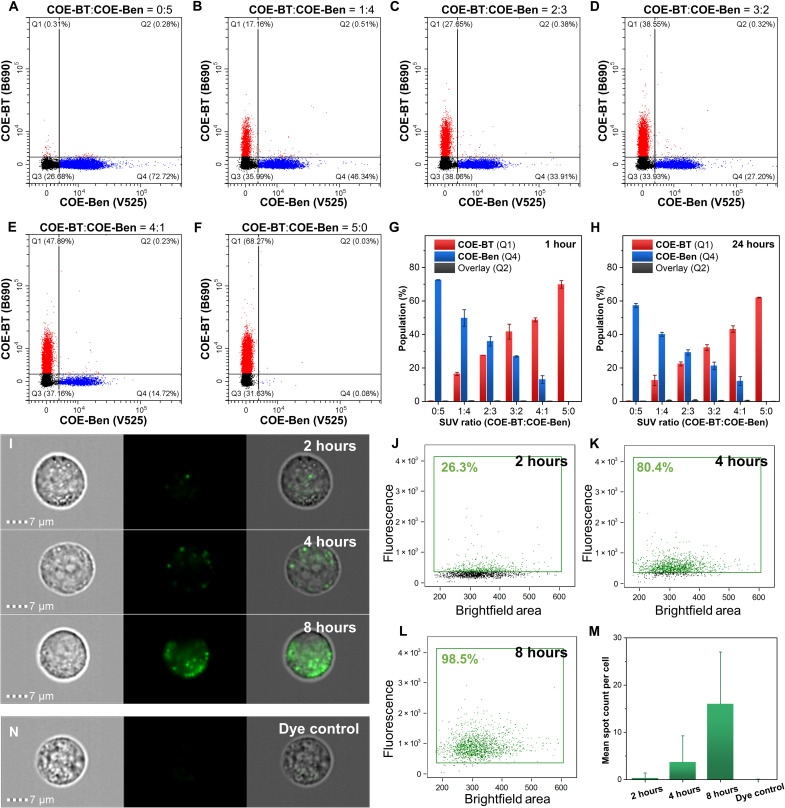
Time-lapsed tracking of small EV uptake into mammalian cells. (**A** to **F**) Flow cytometry measurements for the mixtures of **COE-BT**–stained and **COE-Ben**–stained SUVs in PBS with different mixing ratios. Data were collected on a CytoFLEX LX flow cytometer (Beckman Coulter). The excitation/emission wavelengths for the **COE-BT** (B690) and **COE-Ben** (V525) channels are 488/690 and 405/525 nm, respectively. The size was detected by the VSSC channel. (**G** and **H**) SUV population percentage in different gates after mixing for 1 or 24 hours, respectively. (**I** to **L**) Representative imaging flow cytometry images of A549 cells stained by the **COE-Ben**–stained small EVs for different treatment times and their corresponding flow cytometric analysis. The **COE-Ben** channel was excited using 405 nm, the emission was collected in the range of 505 to 560 nm, and the brightfield area of the whole cell was used as the indication of size. (**M**) Mean spot count of **COE-Ben**–labeled small EVs in each cell. (**N**) Representative imaging flow cytometry images of A549 cells after being stained by the residue of **COE-Ben** after washing using ultrafiltration.

Fluorescent labels are useful for tracking the fate of small EVs after they are taken up by cells (*55*). It is therefore important that these labels bind stably to their small EV targets. Dyes falling off their targets may result in unspecific labeling, leading to misleading signatures in uptake and biodistribution experiments (*56*). We evaluated the binding stability of COEs using an SUV lipid bilayer (POPC:POPG = 85:15). Two samples were first prepared by labeling 1 mM SUVs with either 20 μM **COE-Ben** or 40 μM **COE-BT**. The difference in the PLQY values of both COEs accounts for the different concentrations used here. The two SUV populations were mixed in different volumetric ratios before single-particle analysis using flow cytometry (Fig. 7, A to F, and fig. S37). Figure 7G shows that the percentage of **COE-BT** positive events in the Q1 regions (**COE-BT** channel) increased progressively with the increase in **COE-BT**:**COE-Ben** ratio and vice versa for the **COE-Ben**–positive events in the Q4 regions (**COE-Ben** channel). We observed a negligible percentage of positive events for both **COE-BT** and **COE-Ben**, wherein ≤0.5% dual-positive events were reported in all Q3 regions. Even after 24 hours of incubation at room temperature after mixing, the percentage of dual-positive particles in Q3 remained low (≤0.5%; Fig. 7H and fig. S38). This study demonstrates that COEs are retained in the lipid bilayer of SUVs under these experimental conditions, and no interparticle dye transfer has occurred.

We examined whether the binding stability of COEs would enable a single-particle analysis to monitor the uptake of small EVs into mammalian cells. PC-3 small EVs were labeled with 1 μM **COE-Ben** for 1 hour at room temperature before being washed by ultrafiltration using a 100,000 Da cutoff centrifugal filter unit. The purified stained small EVs were then added to A549 cells in FBS-free DMEM (Dulbecco’s modified Eagle’s medium), followed by incubation at 37°C for different periods of time (2, 4, and 8 hours). These cells were then analyzed using imaging flow cytometry (ImageStream, Amnis). Figure 7I and fig. S39 show that individual spot-like fluorescent signals were located within the A549 cells after they were treated with the **COE-Ben**–labeled small EVs. These fluorescent patterns differed from those of cells being directly labeled with **COE-Ben** for 30 min, in which the fluorescence signals were dispersed and occupied nearly the entire cell (fig. S40). Noticeably, the number of internalized small EVs (i.e., emissive spots) per cell increased with longer incubation periods. These results suggest that the **COE-Ben**–labeled small EVs maintained their integrity after cellular uptake.

Analysis of the small EV uptake experiments using imaging flow cytometry data shows that the **COE-Ben**–positive cell population increased with incubation (2 to 8 hours; Fig. 7, J to L). Consequently, 98% of cells were positive for **COE-Ben**–labeled small EVs after 8 hours (Fig. 7L), with a mean spot number of 16 per cell after analyzing more than 1000 cells (Fig. 7M). We repeated the experiment with a COE-only control, i.e., in the absence of any small EVs. After the same ultrafiltration procedures, any remaining sample on the filter membrane was resuspended in FBS-free DMEM and introduced to the A549 cells at 37°C for 8 hours. The absence of any fluorescent signals when analyzed on the **COE-Ben** channel (Fig. 7N), together with a negligible dye-positive cell subpopulation (2%; fig. S41), suggests that the excess and unbound COE had been readily removed by ultrafiltration in the small EV tracking experiments above.

Similar EV tracking experiments were performed for **DiD**, for which the fluorescent signals were indistinguishable as single particles (fig. S42). These fluorescent patterns are likely contributed by the formation of **DiD** nanoaggregates that cannot be purified using ultrafiltration (fig. S41). In contrast, COE provided a lower background noise, higher cell labeling percentage, and more EV spot numbers within cells, demonstrating their use as a high-efficiency and accurate optical label to track the biodistribution of small EVs (fig. S43).

### Endogenous labeling of small EVs using COEs

**Fig. 8.**
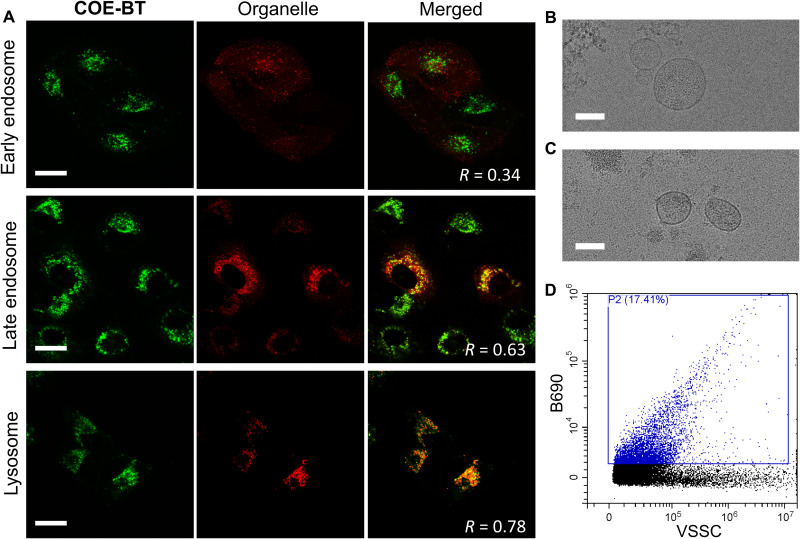
Endogenous labeling of small EVs using COEs. (**A**) Colocalization micrographs of A549 cells after being incubated with 2 μM **COE-BT** and then stained by Early or Late Endosomes-GFP reagent (BacMam 2.0) or 100 nM lysosome-specific dye LysoTracker Green DND-26. The **COE-BT** channel (represented in red) was observed by excitation at 561 nm and collecting the emission in the range of 570 to 620 nm. The GFP and LysoTracker Green channels (represented in green) were observed by excitation at 488 nm and collecting the emission in the range of 500 to 540 nm. Pearson’s correlation coefficient (*R*) as the metric of colocalization was analyzed using ImageJ. Scale bars, 20 μm. (**B** and **C**) Cryo-TEM images of small EVs secreted by A549 cells (B) without or (C) with **COE-BT** endogenous staining. Scale bars, 100 nm. (**D**) Flow cytometry analysis of small EVs secreted by **COE-BT**–stained A549 cells after the first 24-hour incubation. The excitation and emission wavelengths are 488 and 690 nm, respectively, for the B690 channel.

Endogenous labeling of small EVs has been reported, wherein small EVs can be labeled before harvesting from the cell culture medium (*57*). This procedure is typically done by allowing cells to incubate with the dye throughout their growth cycle so that the dyes can be incorporated into the small EVs during biogenesis. We thus investigated using the noncytotoxic and lipid bilayer–mimicking COEs to endogenously label small EVs. Previous work showed that COEs would label the cellular membrane and be internalized after 24 hours of incubation (*8*). Here, we further probed the intracellular localization of COEs to determine whether cellular trafficking will colocalize COEs with organelles for small EV biogenesis. Accordingly, A549 cells were first labeled with 2 μM **COE-BT** that will not affect cell viability (Fig. 5D) and left to incubate overnight. We used confocal microscopy to investigate the colocalization of the internalized COEs, early and late endosomes, and lysosomes. Lentiviral vectors (CellLight Early/Late Endosomes-GFP, Thermo Fisher Scientific) were used to induce cells to produce green fluorescent proteins (GFPs) that were fused to Rab5a or Rab7a proteins found in the early and late endosomes, respectively. The LysoTracker Green dye was used for tracking the lysosomes (Fig. 8A). The colocalization analyses were estimated by calculating the Pearson correlation coefficient using the Coloc2 plugin on the ImageJ software (fig. S44) (*58*). It was estimated that the internalized **COE-BT** had a 34% chance of colocalization with the early endosomes, 63% for late endosomes, and 78% for lysosomes. These data imply that **COE-BT** can label the membrane structures of different organelles in the cytoplasm after internalization (*7*).

To validate endogenous small EV labeling, A549 cells were stained with 2 μM **COE-BT** and incubated in DMEM supplementing exosome-depleted FBS. After 24 hours of incubation, the cell culture supernatant was collected, and a series of ultracentrifugation steps were carried out to isolate any prestained small EVs (*28*). Cryo-TEM confirmed the presence of the small EVs secreted by **COE-BT**–stained A549 cells after harvesting and purification, and the morphology of EVs did not obviously change in endogenous labeling (Fig. 8, B and C). Dye-positive events were directly detected in flow cytometry for these small EVs without the need of an exogenous dye. The flow cytometry data suggest that at least 17% of the small EV population has been endogenously labeled with **COE-BT** (Fig. 8C). These **COE-BT**–stained A549 cells were also found to continuously produce labeled small EVs for the next 24 hours after the first harvest (fig. S45). Although future COEs for internalization specific to the endosome are expected to increase the COE-positive population further, these results indicate that COEs can be helpful in endogenous small EV labeling strategies, in addition to the direct labeling and tracking of small EVs.

## DISCUSSION

Eight COEs are described here with optical and water-soluble features that encourage their use as membrane-targeting probes for small EV labeling. Their molecular lengths and chemical structures are designed to mimic the dimensions and hydrophobic and hydrophilic distributions of lipid bilayers. Furthermore, the presence of six cationic charges per molecule ensures solubility in aqueous media, despite the extended hydrophobic conjugated segments. This molecular feature minimizes the formation of nanoaggregates at concentrations useful for labeling biological nanoparticles. The increase in emission intensity when COEs are located within lipid bilayers is advantageous for minimizing the background emission of the fluorophore. Through tailoring the conjugated moieties, we have achieved a series of COEs that have emissions from the UV to the NIR-II regions of the spectrum without influencing membrane interactions.

The general characteristics described above for COEs were used to label small EVs directly. The number of false-positive signals observed in flow cytometry measurements is sufficiently low to allow for detection. As a result of the stable binding within lipid membranes, it is possible to track small EV uptake in cells. From a practical perspective for method development, the absence of COE aggregation is key to enabling excess-free dye removal through easy-to-use ultrafiltration or dialysis. Moreover, the lipid bilayer–mimicking structure confers COE good compatibility when bound to the membrane. Hence, COE treatment unaltered the size of small EVs nor induced toxicity to cells. Furthermore, COEs can be used to endogenously label small EVs by prestaining mammalian cells. These results support that COEs serve as a versatile platform to improve the detection of biological particles across multiple length scales.

## MATERIALS AND METHODS

### CytoFLEX LX flow cytometry of small EVs

Lyophilized PC-3 small EVs derived from cell culture were purchased from Abcam (ab239689). A fresh vial of 100 μg was reconstituted in 1 ml of MiliQ water. The small EV sample was diluted to 10 μg ml^−1^ using PBS. Lyophilized solids of all COEs were solubilized to a concentration of 100 μM in PBS and heated and sonicated at 45°C for 20 min to facilitate the dissolving process. COEs were diluted using PBS to their working concentration. Small EV solution (10 μg ml^−1^) was mixed with each dye working solution in a 1.5-ml Eppendorf tube to different concentrations (0.5 μM for **COE-B7** and **COE-Ben**; 1 μM for **COE-S6**, **COE-QX**, **COE-BO**, and **COE-BT**; and 2.5 μM for **COE-BSe**). Samples were incubated in the dark at room temperature for 1 to 1.5 hours. PBS was added to dilute each sample 100 times before performing flow cytometry measurements.

Flow cytometry experiments were performed on the Beckman Coulter CytoFLEX LX using the VSSC configuration as described in previous literature (*52*). Briefly, the blue SSC (488 nm) was modified to VSSC (405 nm) by moving the 405/10 VSSC filter to the V450 channel in the wavelength-division multiplexing. Latex beads (100 and 300 nm) were used for calibration. The gain setting for the V405 channel (VSSC) was set to 450. All other channel gains were set to 3000. Detection was triggered on the VSSC > 4000. The fluorescence of **COE-B7** was measured using a spare 450-nm bandpass filter in place of the UV675 channel. Before the acquisition of samples, the instrument was washed for 15 min with Contrad 70 detergent (Beckman Coulter) and for another 30 min using deionized (DI) water. Once events for DI water were low (<1000 μl^−1^), the instrument was deemed ready for data collection. Samples were boosted by running for 1 min on the high flow rate (60 μl min^−1^) and then changed to slow (10 μl min^−1^). Data collection was terminated by controlling the sample volume (10.0 μl). Data were analyzed using CytExpert software. As shown in the resulting figures, positive gates were set to exclude events from the small EV–only sample.

### Nano–flow cytometry analysis on SW480 small EVs

Small EVs isolated from SW480 colon cancer cell lines and purified using size exclusion chromatography were provided by NanoFCM (UK). The labeling and flow cytometry analysis were also carried out by the trained technicians at NanoFCM (UK). Briefly, the small EVs were labeled at a particle concentration of approximately 10^10^ particles/ml, in which 9 μl of the sample was added with 1 μl of 10 μM COE dye solutions to achieve a final 1 μM staining concentration. The mixture was left to incubate at room temperature for 30 min and then diluted 100 times using PBS before analysis on the NanoAnalyzer (NanoFCM). The small EVs were detected on the small threshold settings (68-155 S16M-Exo), and the labeled population was analyzed on the PC5 channel (excitation, 488/emission, 670). Dye control samples were prepared by replacing the 9 μl of small EV solution with PBS, followed by the same steps for the small EV samples for flow analysis. The results were validated through at least two replicates.

### Imaging flow cytometry

A549 cells in DMEM containing 10% FBS were seeded at a concentration of 1 × 10^5^ cells/ml in a six-well plate and allowed to adhere overnight before experiments. The next day, 4.5 ml of PC-3 (10 μg ml^−1^) small EVs or controls (PBS only) was mixed with **COE-Ben** to a final dye concentration of 1 μM or **DiD** to a final dye concentration of 2.5 μM. Samples were incubated at room temperature for 1 hour in the dark. Samples were washed by filtration using a 100,000 Da-cutoff centrifugal filter unit (Amicon Ultra) and centrifuged at 2000*g* for 15 min to remove the free dye. The concentrated samples were recovered and resuspended in FBS-free DMEM, which were divided into three aliquots. The cells in microplates were rinsed using FBS-free DMEM before stained small EVs or controls (residual dye solutions) were added at different time points, such as 2, 4, and 8 hours before harvesting the cells. After small EV or dye uptake, cells were washed with warmed PBS (37°C), and 1× trypsin-EDTA was added. The cells were left to incubate in the 5% CO_2_ chamber at 37°C for up to 5 min. DMEM containing 10% FBS was added into microplates, and cells were centrifuged at 200*g* for 4 min. The supernatant was removed, and cells were resuspended in DMEM (containing 10% FBS) and taken for imaging flow cytometry using an Amnis ImageStream^X^ Mk II Imaging Flow Cytometer. The collection gate was set using the area versus aspect ratio of the brightfield channel to select for intact cell events only. For the **COE-Ben** channel (i.e., Ch02), the cells were excited using a 405-nm laser, and the emission was collected in the range of 505 to 560 nm. For the **DiD** channel (i.e., Ch05), the cells were excited using a 638-nm laser, and the emission was collected in the range of 642 to 745 nm. The brightfield area of the whole cell was used as the indication of size, i.e., the M04 or M01 channel. Data were processed using IDEAS v6.3. A gradient root mean square on the brightfield channel was applied to exclude cells out of focus. The flow cytometry results were presented using the M04 channel (size) versus the Ch02 channel (**COE-Ben**) or Ch05 channel (**DiD**). Single cells were gated using a plot of the area versus aspect ratio of the brightfield channel. Gates were set according to the unstained control samples. The built-in “spot count algorithm” function in IDEAS 6.3 software was used to count the number of bright spots within the cell and used as a metric for exosome uptake. More than 25 images of high spot count and more than 25 images of low spot count were manually identified as truth populations on the fluorescent channel of interest before running the analysis. At least 1000 cells were counted in each group.

### Production of COE-prestained small EVs

To distinguish between small EVs produced by the A549 cells and the small EVs that originate from serum, a fresh cell culture media (CCM-Exo-Dep) was prepared for the small EV production by supplementing DMEM with exosome-depleted FBS (Systems Biosciences, catalog EXO-FBS-250A-1). The A549 cells were seeded at 5 × 10^5^ cells/ml in 10 ml of CCM-Exo-Dep within T75 flasks and left to adhere for 1 hour before 2 μM **COE-BT** was introduced into the cell culture. The cells were left to incubate for at least 24 hours before the cell culture supernatant was recovered for the first round of small EV production and stored at 4°C before purification. Without passaging the labeled cells, 10 ml of fresh CCM-Exo-Dep was added to the cells and left to incubate for at least another 24 hours. The cell culture supernatant for the second round of small EV production was recovered after 24 hours. The collected cell cultures were first spun at 10,000*g* for 30 min at room temperature to remove any cellular debris. Small EV isolation was then carried out via ultracentrifugation (Optima XPN-100, Beckman Coulter) at 100,000*g* for 1 hour at 4°C. The supernatant was carefully removed, and the bottom of the centrifuge tube, at which the pellet is expected to be located, was rinsed three times with PBS. The small EVs were pelleted again under the same conditions after washing and then resuspended in 100 μl of PBS. Aliquots of the purified small EVs were stored at −80°C before downstream analysis (flow cytometry or TEM).

## Supplementary Materials

### This PDF file includes:

Figs. S1 to S94 Supplementary Measurements Synthesis Details

## Notes

View/request a protocol for this paper from *Bio-protocol*.

## References

[1] L. E. Garner, J. Park, S. M. Dyar, A. Chworos, J. J. Sumner, G. C. Bazan, Modification of the optoelectronic properties of membranes via insertion of amphiphilic phenylenevinylene oligoelectrolytes. J. Am. Chem. Soc. 132, 10042–10052 (2010). 20608655 10.1021/ja1016156

[2] H. Y. Woo, B. Liu, B. Kohler, D. Korystov, A. Mikhailovsky, G. C. Bazan, Solvent effects on the two-photon absorption of distyrylbenzene chromophores. J. Am. Chem. Soc. 127, 14721–14729 (2005). 16231926 10.1021/ja052906g

[3] C. Zhou, G. W. N. Chia, K.-T. Yong, Membrane-intercalating conjugated oligoelectrolytes. Chem. Soc. Rev. 51, 9917–9932 (2022). 36448452 10.1039/d2cs00014h

[4] C. Zhou, J. C. S. Ho, G. W. N. Chia, A. S. Moreland, L. Ruan, B. Liedberg, S. Kjelleberg, J. Hinks, G. C. Bazan, Gram-typing using conjugated oligoelectrolytes. Adv. Funct. Mater. 30, 2004068 (2020).

[5] C. Zhou, G. W. N. Chia, J. C. S. Ho, A. S. Moreland, T. Seviour, B. Liedberg, A. N. Parikh, S. Kjelleberg, J. Hinks, G. C. Bazan, A chain-elongated oligophenylenevinylene electrolyte increases microbial membrane stability. Adv. Mater. 31, 1808021 (2019). 10.1002/adma.201808021 30908801

[6] J. Hinks, W. H. Poh, J. J. H. Chu, J. S. C. Loo, G. C. Bazan, L. E. Hancock, S. Wuertz, Oligopolyphenylenevinylene-conjugated oligoelectrolyte membrane insertion molecules selectively disrupt cell envelopes of gram-positive bacteria. Appl. Environ. Microbiol. 81, 1949–1958 (2015). 25576607 10.1128/AEM.03355-14 PMC4345381

[7] P. Gwozdzinska, R. Pawlowska, J. Milczarek, L. E. Garner, A. W. Thomas, G. C. Bazan, A. Chworos, Phenylenevinylene conjugated oligoelectrolytes as fluorescent dyes for mammalian cell imaging. Chem. Commun. 50, 14859–14861 (2014). 10.1039/c4cc06478j 25322778

[8] C. Zhou, Z. Li, Z. Zhu, G. W. N. Chia, A. Mikhailovsky, R. J. Vázquez, S. J. W. Chan, K. Li, B. Liu, G. C. Bazan, Conjugated oligoelectrolytes for long-term tumor tracking with incremental NIR-II emission. Adv. Mater. 34, 2201989 (2022). 10.1002/adma.202201989 35306702

[9] M. Tkach, C. Théry, Communication by extracellular vesicles: Where we are and where we need to go. Cell 164, 1226–1232 (2016). 26967288 10.1016/j.cell.2016.01.043

[10] C. Théry, K. W. Witwer, E. Aikawa, M. J. Alcaraz, J. D. Anderson, R. Andriantsitohaina, A. Antoniou, T. Arab, F. Archer, G. K. Atkin-Smith, D. C. Ayre, J.-M. Bach, D. Bachurski, H. Baharvand, L. Balaj, S. Baldacchino, N. N. Bauer, A. A. Baxter, M. Bebawy, C. Beckham, A. B. Zavec, A. Benmoussa, A. C. Berardi, P. Bergese, E. Bielska, C. Blenkiron, S. Bobis-Wozowicz, E. Boilard, W. Boireau, A. Bongiovanni, F. E. Borràs, S. Bosch, C. M. Boulanger, X. Breakefield, A. M. Breglio, M. Á. Brennan, D. R. Brigstock, A. Brisson, M. L. D. Broekman, J. F. Bromberg, P. Bryl-Górecka, S. Buch, A. H. Buck, D. Burger, S. Busatto, D. Buschmann, B. Bussolati, E. I. Buzás, J. B. Byrd, G. Camussi, D. R. F. Carter, S. Caruso, L. W. Chamley, Y.-T. Chang, C. Chen, S. Chen, L. Cheng, A. R. Chin, A. Clayton, S. P. Clerici, A. Cocks, E. Cocucci, R. J. Coffey, A. Cordeiro-da-Silva, Y. Couch, F. A. W. Coumans, B. Coyle, R. Crescitelli, M. F. Criado, C. D’Souza-Schorey, S. Das, A. D. Chaudhuri, P. de Candia, E. F. De Santana, O. De Wever, H. A. del Portillo, T. Demaret, S. Deville, A. Devitt, B. Dhondt, D. Di Vizio, L. C. Dieterich, V. Dolo, A. P. D. Rubio, M. Dominici, M. R. Dourado, T. A. P. Driedonks, F. V. Duarte, H. M. Duncan, R. M. Eichenberger, K. Ekström, S. El Andaloussi, C. Elie-Caille, U. Erdbrügger, J. M. Falcón-Pérez, F. Fatima, J. E. Fish, M. Flores-Bellver, A. Försönits, A. Frelet-Barrand, F. Fricke, G. Fuhrmann, S. Gabrielsson, A. Gámez-Valero, C. Gardiner, K. Gärtner, R. Gaudin, Y. S. Gho, B. Giebel, C. Gilbert, M. Gimona, I. Giusti, D. C. I. Goberdhan, A. Görgens, S. M. Gorski, D. W. Greening, J. C. Gross, A. Gualerzi, G. N. Gupta, D. Gustafson, A. Handberg, R. A. Haraszti, P. Harrison, H. Hegyesi, A. Hendrix, A. F. Hill, F. H. Hochberg, K. F. Hoffmann, B. Holder, H. Holthofer, B. Hosseinkhani, G. Hu, Y. Huang, V. Huber, S. Hunt, A. G.-E. Ibrahim, T. Ikezu, J. M. Inal, M. Isin, A. Ivanova, H. K. Jackson, S. Jacobsen, S. M. Jay, M. Jayachandran, G. Jenster, L. Jiang, S. M. Johnson, J. C. Jones, A. Jong, T. Jovanovic-Talisman, S. Jung, R. Kalluri, S.-I. Kano, S. Kaur, Y. Kawamura, E. T. Keller, D. Khamari, E. Khomyakova, A. Khvorova, P. Kierulf, K. P. Kim, T. Kislinger, M. Klingeborn, D. J. Klinke, M. Kornek, M. M. Kosanović, Á. F. Kovács, E.-M. Krämer-Albers, S. Krasemann, M. Krause, I. V. Kurochkin, G. D. Kusuma, S. Kuypers, S. Laitinen, S. M. Langevin, L. R. Languino, J. Lannigan, C. Lässer, L. C. Laurent, G. Lavieu, E. Lázaro-Ibáñez, S. Le Lay, M.-S. Lee, Y. X. F. Lee, D. S. Lemos, M. Lenassi, A. Leszczynska, I. T. S. Li, K. Liao, S. F. Libregts, E. Ligeti, R. Lim, S. K. Lim, A. Linē, K. Linnemannstöns, A. Llorente, C. A. Lombard, M. J. Lorenowicz, Á. M. Lörincz, J. Lötvall, J. Lovett, M. C. Lowry, X. Loyer, Q. Lu, B. Lukomska, T. R. Lunavat, S. L. N. Maas, H. Malhi, A. Marcilla, J. Mariani, J. Mariscal, E. S. Martens-Uzunova, L. Martin-Jaular, M. C. Martinez, V. R. Martins, M. Mathieu, S. Mathivanan, M. Maugeri, L. K. McGinnis, M. J. McVey, D. G. Meckes, K. L. Meehan, I. Mertens, V. R. Minciacchi, A. Möller, M. M. Jørgensen, A. Morales-Kastresana, J. Morhayim, F. Mullier, M. Muraca, L. Musante, V. Mussack, D. C. Muth, K. H. Myburgh, T. Najrana, M. Nawaz, I. Nazarenko, P. Nejsum, C. Neri, T. Neri, R. Nieuwland, L. Nimrichter, J. P. Nolan, E. N. M. Nolte-‘t Hooten, N. N. Hooten, L. O’Driscoll, T. O’Grady, A. O’Loghlen, T. Ochiya, M. Olivier, A. Ortiz, L. A. Ortiz, X. Osteikoetxea, O. Østergaard, M. Ostrowski, J. Park, D. M. Pegtel, H. Peinado, F. Perut, M. W. Pfaffl, D. G. Phinney, B. C. H. Pieters, R. C. Pink, D. S. Pisetsky, E. P. von Strandmann, I. Polakovicova, I. K. H. Poon, B. H. Powell, I. Prada, L. Pulliam, P. Quesenberry, A. Radeghieri, R. L. Raffai, S. Raimondo, J. Rak, M. I. Ramirez, G. Raposo, M. S. Rayyan, N. Regev-Rudzki, F. L. Ricklefs, P. D. Robbins, D. D. Roberts, S. C. Rodrigues, E. Rohde, S. Rome, K. M. A. Rouschop, A. Rughetti, A. E. Russell, P. Saá, S. Sahoo, E. Salas-Huenuleo, C. Sánchez, J. A. Saugstad, M. J. Saul, R. M. Schiffelers, R. Schneider, T. H. Schøyen, A. Scott, E. Shahaj, S. Sharma, O. Shatnyeva, F. Shekari, G. V. Shelke, A. K. Shetty, K. Shiba, P. R. M. Siljander, A. M. Silva, A. Skowronek, O. L. Snyder, R. P. Soares, B. W. Sódar, C. Soekmadji, J. Sotillo, P. D. Stahl, W. Stoorvogel, S. L. Stott, E. F. Strasser, S. Swift, H. Tahara, M. Tewari, K. Timms, S. Tiwari, R. Tixeira, M. Tkach, W. S. Toh, R. Tomasini, A. C. Torrecilhas, J. P. Tosar, V. Toxavidis, L. Urbanelli, P. Vader, B. W. M. van Balkom, S. G. van der Grein, J. Van Deun, M. J. C. van Herwijnen, K. Van Keuren-Jensen, G. van Niel, M. E. van Royen, A. J. van Wijnen, M. H. Vasconcelos, I. J. Vechetti, T. D. Veit, L. J. Vella, É. Velot, F. J. Verweij, B. Vestad, J. L. Viñas, T. Visnovitz, K. V. Vukman, J. Wahlgren, D. C. Watson, M. H. M. Wauben, A. Weaver, J. P. Webber, V. Weber, A. M. Wehman, D. J. Weiss, J. A. Welsh, S. Wendt, A. M. Wheelock, Z. Wiener, L. Witte, J. Wolfram, A. Xagorari, P. Xander, J. Xu, X. Yan, M. Yáñez-Mó, H. Yin, Y. Yuana, V. Zappulli, J. Zarubova, V. Žėkas, J.-Y. Zhang, Z. Zhao, L. Zheng, A. R. Zheutlin, A. M. Zickler, P. Zimmermann, A. M. Zivkovic, D. Zocco, E. K. Zuba-Surma, Minimal information for studies of extracellular vesicles 2018 (MISEV 2018): A position statement of the International Society for Extracellular Vesicles and update of the MISEV2014 guidelines. J. Extracell. Vesicles 7, 1535750 (2018). 30637094 10.1080/20013078.2018.1535750 PMC6322352

[11] X. Li, A. L. Corbett, E. Taatizadeh, N. Tasnim, J. P. Little, C. Garnis, M. Daugaard, E. Guns, M. Hoorfar, I. T. S. Li, Challenges and opportunities in exosome research—Perspectives from biology, engineering, and cancer therapy. APL Bioeng. 3, 011503 (2019). 31069333 10.1063/1.5087122 PMC6481742

[12] M. Yáñez-Mó, P. R. M. Siljander, Z. Andreu, A. B. Zavec, F. E. Borràs, E. I. Buzas, K. Buzas, E. Casal, F. Cappello, J. Carvalho, E. Colás, A. C.-d. Silva, S. Fais, J. M. Falcon-Perez, I. M. Ghobrial, B. Giebel, M. Gimona, M. Graner, I. Gursel, M. Gursel, N. H. H. Heegaard, A. Hendrix, P. Kierulf, K. Kokubun, M. Kosanovic, V. Kralj-Iglic, E.-M. Krämer-Albers, S. Laitinen, C. Lässer, T. Lener, E. Ligeti, A. Linē, G. Lipps, A. Llorente, J. Lötvall, M. Manček-Keber, A. Marcilla, M. Mittelbrunn, I. Nazarenko, E. N. M. Nolte-‘t Hoen, T. A. Nyman, L. O’Driscoll, M. Olivan, C. Oliveira, É. Pállinger, H. A. del Portillo, J. Reventós, M. Rigau, E. Rohde, M. Sammar, F. Sánchez-Madrid, N. Santarém, K. Schallmoser, M. S. Ostenfeld, W. Stoorvogel, R. Stukelj, S. G. Van der Grein, M. H. Vasconcelos, M. H. M. Wauben, O. De Wever, Biological properties of extracellular vesicles and their physiological functions. J. Extracell. Vesicles 4, 27066 (2015). 25979354 10.3402/jev.v4.27066 PMC4433489

[13] S. Fu, Y. Zhang, Y. Li, L. Luo, Y. Zhao, Y. Yao, Extracellular vesicles in cardiovascular diseases. Cell Death Discov. 6, 68 (2020). 32821437 10.1038/s41420-020-00305-y PMC7393487

[14] A. G. Thompson, E. Gray, S. M. Heman-Ackah, I. Mäger, K. Talbot, S. E. Andaloussi, M. J. Wood, M. R. Turner, Extracellular vesicles in neurodegenerative disease—Pathogenesis to biomarkers. Nat. Rev. Neurol. 12, 346–357 (2016). 27174238 10.1038/nrneurol.2016.68

[15] R. Tutrone, M. J. Donovan, P. Torkler, V. Tadigotla, T. McLain, M. Noerholm, J. Skog, J. McKiernan, Clinical utility of the exosome based ExoDx prostate (IntelliScore) EPI test in men presenting for initial biopsy with a PSA 2–10 ng/mL. Prostate Cancer Prostatic Dis. 23, 607–614 (2020). 32382078 10.1038/s41391-020-0237-z PMC7655505

[16] P. D. Robbins, A. E. Morelli, Regulation of immune responses by extracellular vesicles. Nat. Rev. Immunol. 14, 195–208 (2014). 24566916 10.1038/nri3622 PMC4350779

[17] O. G. de Jong, S. A. A. Kooijmans, D. E. Murphy, L. Jiang, M. J. W. Evers, J. P. G. Sluijter, P. Vader, R. M. Schiffelers, Drug delivery with extracellular vesicles: From imagination to innovation. Acc. Chem. Res. 52, 1761–1770 (2019). 31181910 10.1021/acs.accounts.9b00109 PMC6639984

[18] C. K. Das, B. C. Jena, I. Banerjee, S. Das, A. Parekh, S. K. Bhutia, M. Mandal, Exosome as a novel shuttle for delivery of therapeutics across biological barriers. Mol. Pharm. 16, 24–40 (2019). 30513203 10.1021/acs.molpharmaceut.8b00901

[19] S. Fais, L. O’Driscoll, F. E. Borras, E. Buzas, G. Camussi, F. Cappello, J. Carvalho, A. C. da Silva, H. Del Portillo, S. El Andaloussi, T. F. Trček, R. Furlan, A. Hendrix, I. Gursel, V. Kralj-Iglic, B. Kaeffer, M. Kosanovic, M. E. Lekka, G. Lipps, M. Logozzi, A. Marcilla, M. Sammar, A. Llorente, I. Nazarenko, C. Oliveira, G. Pocsfalvi, L. Rajendran, G. Raposo, E. Rohde, P. Siljander, G. van Niel, M. H. Vasconcelos, M. Yáñez-Mó, M. L. Yliperttula, N. Zarovni, A. B. Zavec, B. Giebel, Evidence-based clinical use of nanoscale extracellular vesicles in nanomedicine. ACS Nano 10, 3886–3899 (2016). 26978483 10.1021/acsnano.5b08015

[20] S. T.-Y. Chuo, J. C.-Y. Chien, C. P.-K. Lai, Imaging extracellular vesicles: Current and emerging methods. J. Biomed. Sci. 25, 91 (2018). 30580764 10.1186/s12929-018-0494-5 PMC6304785

[21] F. J. Verweij, L. Balaj, C. M. Boulanger, D. R. F. Carter, E. B. Compeer, G. D’Angelo, S. El Andaloussi, J. G. Goetz, J. C. Gross, V. Hyenne, E.-M. Krämer-Albers, C. P. Lai, X. Loyer, A. Marki, S. Momma, E. N. M. Nolte-‘t Hoen, D. M. Pegtel, H. Peinado, G. Raposo, K. Rilla, H. Tahara, C. Théry, M. E. van Royen, R. E. Vandenbroucke, A. M. Wehman, K. Witwer, Z. Wu, R. Wubbolts, G. van Niel, The power of imaging to understand extracellular vesicle biology in vivo. Nat. Methods 18, 1013–1026 (2021). 34446922 10.1038/s41592-021-01206-3 PMC8796660

[22] H. Shao, H. Im, C. M. Castro, X. Breakefield, R. Weissleder, H. Lee, New technologies for analysis of extracellular vesicles. Chem. Rev. 118, 1917–1950 (2018). 29384376 10.1021/acs.chemrev.7b00534 PMC6029891

[23] J. A. Welsh, J. A. Holloway, J. S. Wilkinson, N. A. Englyst, Extracellular vesicle flow cytometry analysis and standardization. Front. Cell Dev. Biol. 5, 78 (2017). 28913335 10.3389/fcell.2017.00078 PMC5582084

[24] T. Arab, E. R. Mallick, Y. Huang, L. Dong, Z. Liao, Z. Zhao, O. Gololobova, B. Smith, N. J. Haughey, K. J. Pienta, B. S. Slusher, P. M. Tarwater, J. P. Tosar, A. M. Zivkovic, W. N. Vreeland, M. E. Paulaitis, K. W. Witwer, Characterization of extracellular vesicles and synthetic nanoparticles with four orthogonal single-particle analysis platforms. J. Extracell. Vesicles 10, e12079 (2021). 33850608 10.1002/jev2.12079 PMC8023330

[25] J. P. Nolan, Flow cytometry of extracellular vesicles: Potential, pitfalls, and prospects. Curr. Protoc. Cytom. 73, 13.14.11–13.14.16 (2015). 10.1002/0471142956.cy1314s73 26132176

[26] J. A. Welsh, E. Van Der Pol, G. J. A. Arkesteijn, M. Bremer, A. Brisson, F. Coumans, F. Dignat-George, E. Duggan, I. Ghiran, B. Giebel, A. Görgens, A. Hendrix, R. Lacroix, J. Lannigan, S. F. W. M. Libregts, E. Lozano-Andrés, A. Morales-Kastresana, S. Robert, L. De Rond, T. Tertel, J. Tigges, O. De Wever, X. Yan, R. Nieuwland, M. H. M. Wauben, J. P. Nolan, J. C. Jones, MIFlowCyt-EV: A framework for standardized reporting of extracellular vesicle flow cytometry experiments. J. Extracell. Vesicles 9, 1713526 (2020). 32128070 10.1080/20013078.2020.1713526 PMC7034442

[27] H. Lian, S. He, C. Chen, X. Yan, Flow cytometric analysis of nanoscale biological particles and organelles. Annu. Rev. Anal. Chem. 12, 389–409 (2019). 10.1146/annurev-anchem-061318-115042 30978294

[28] E. J. van der Vlist, E. N. M. Nolte-’t Hoen, W. Stoorvogel, G. J. A. Arkesteijn, M. H. M. Wauben, Fluorescent labeling of nano-sized vesicles released by cells and subsequent quantitative and qualitative analysis by high-resolution flow cytometry. Nat. Protoc. 7, 1311–1326 (2012). 22722367 10.1038/nprot.2012.065

[29] L. de Rond, E. van der Pol, C. M. Hau, Z. Varga, A. Sturk, T. G. van Leeuwen, R. Nieuwland, F. A. W. Coumans, Comparison of generic fluorescent markers for detection of extracellular vesicles by flow cytometry. Clin. Chem. 64, 680–689 (2018). 29453194 10.1373/clinchem.2017.278978

[30] P. J. Sims, A. S. Waggoner, C.-H. Wang, J. F. Hoffman, Mechanism by which cyanine dyes measure membrane potential in red blood cells and phosphatidylcholine vesicles. Biochemistry 13, 3315–3330 (1974). 4842277 10.1021/bi00713a022

[31] Y. W. Yi, J. H. Lee, S.-Y. Kim, C.-G. Pack, D. H. Ha, S. R. Park, J. Youn, B. S. Cho, Advances in analysis of biodistribution of exosomes by molecular imaging. Int. J. Mol. Sci. 21, 665 (2020). 31963931 10.3390/ijms21020665 PMC7014306

[32] M. G. Honig, R. I. Hume, Fluorescent carbocyanine dyes allow living neurons of identified origin to be studied in long-term cultures. J. Cell Biol. 103, 171–187 (1986). 2424918 10.1083/jcb.103.1.171 PMC2113786

[33] P. Pužar Dominkuš, M. Stenovec, S. Sitar, E. Lasič, R. Zorec, A. Plemenitaš, E. Žagar, M. Kreft, M. Lenassi, PKH26 labeling of extracellular vesicles: Characterization and cellular internalization of contaminating PKH26 nanoparticles. Biochim. Biophys. Acta Biomembr. 1860, 1350–1361 (2018). 29551275 10.1016/j.bbamem.2018.03.013

[34] P. Li, M. Kaslan, S. H. Lee, J. Yao, Z. Gao, Progress in exosome isolation techniques. Theranostics 7, 789–804 (2017). 28255367 10.7150/thno.18133 PMC5327650

[35] M. Collot, P. Ashokkumar, H. Anton, E. Boutant, O. Faklaris, T. Galli, Y. Mély, L. Danglot, A. S. Klymchenko, MemBright: A family of fluorescent membrane probes for advanced cellular imaging and neuroscience. Cell Chem. Biol. 26, 600–614.e607 (2019). 30745238 10.1016/j.chembiol.2019.01.009

[36] T. Shimomura, R. Seino, K. Umezaki, A. Shimoda, T. Ezoe, M. Ishiyama, K. Akiyoshi, New lipophilic fluorescent dyes for labeling extracellular vesicles: Characterization and monitoring of cellular uptake. Bioconjug. Chem. 32, 680–684 (2021). 33719402 10.1021/acs.bioconjchem.1c00068

[37] F. Ender, P. Zamzow, N. von Bubnoff, F. Gieseler, Detection and quantification of extracellular vesicles via FACS: Membrane labeling matters! Int. J. Mol. Sci. 21, 291 (2020). 10.3390/ijms21010291 PMC6981603 31906247

[38] Z. Bao, W. K. Chan, L. Yu, Exploration of the stille coupling reaction for the synthesis of functional polymers. J. Am. Chem. Soc. 117, 12426–12435 (1995).

[39] W. Kohn, A. D. Becke, R. G. Parr, Density functional theory of electronic structure. J. Phys. Chem. 100, 12974–12980 (1996).

[40] J. Roncali, Synthetic principles for bandgap control in linear π-conjugated systems. Chem. Rev. 97, 173–206 (1997). 11848868 10.1021/cr950257t

[41] G. van Meer, A. I. P. M. de Kroon, Lipid map of the mammalian cell. J. Cell Sci. 124, 5–8 (2011). 21172818 10.1242/jcs.071233

[42] S. Singh, K. C. Khulbe, T. Matsuura, P. Ramamurthy, Membrane characterization by solute transport and atomic force microscopy. J. Membr. Sci. 142, 111–127 (1998).

[43] P. Van Haver, N. Helsen, S. Depaemelaere, M. Van der Auweraer, F. C. De Schryver, The influence of solvent polarity of the nonradiative decay of exciplexes. J. Am. Chem. Soc. 113, 6849–6857 (1991).

[44] J. Seo, S. Kim, S. Y. Park, Strong solvatochromic fluorescence from the intramolecular charge-transfer state created by excited-state intramolecular proton transfer. J. Am. Chem. Soc. 126, 11154–11155 (2004). 15355088 10.1021/ja047815i

[45] J. R. Lakowicz, *Principles of fluorescence spectroscopy* (Springer, 2006), pp. XXVI, 954.

[46] O. E. Semonin, J. C. Johnson, J. M. Luther, A. G. Midgett, A. J. Nozik, M. C. Beard, Absolute photoluminescence quantum yields of IR-26 dye, PbS, and PbSe quantum dots. J. Phys. Chem. Lett. 1, 2445–2450 (2010).

[47] Y.-C. Wei, S. F. Wang, Y. Hu, L.-S. Liao, D.-G. Chen, K.-H. Chang, C.-W. Wang, S.-H. Liu, W.-H. Chan, J.-L. Liao, W.-Y. Hung, T.-H. Wang, P.-T. Chen, H.-F. Hsu, Y. Chi, P.-T. Chou, Overcoming the energy gap law in near-infrared OLEDs by exciton–vibration decoupling. Nature Photon. 14, 570–577 (2020).

[48] A. D. Thompson, M. H. Omar, F. Rivera-Molina, Z. Xi, A. J. Koleske, D. K. Toomre, A. Schepartz, Long-term live-cell STED nanoscopy of primary and cultured cells with the plasma membrane HIDE probe DiI-SiR. Angew. Chem. Int. Edit. 56, 10408–10412 (2017). 10.1002/anie.201704783 PMC5576494 28679029

[49] X. Zhang, C. Wang, L. Jin, Z. Han, Y. Xiao, Photostable bipolar fluorescent probe for video tracking plasma membranes related cellular processes. ACS Appl. Mater. Interfaces 6, 12372–12379 (2014). 25039476 10.1021/am503849c

[50] M. Dehghani, S. M. Gulvin, J. Flax, T. R. Gaborski, Systematic evaluation of PKH labelling on extracellular vesicle size by nanoparticle tracking analysis. Sci. Rep. 10, 9533 (2020). 32533028 10.1038/s41598-020-66434-7 PMC7293335

[51] M. Montana, F. Mathias, T. Terme, P. Vanelle, Antitumoral activity of quinoxaline derivatives: A systematic review. Eur. J. Med. Chem. 163, 136–147 (2019). 30503938 10.1016/j.ejmech.2018.11.059

[52] G. C. Brittain, Y. Q. Chen, E. Martinez, V. A. Tang, T. M. Renner, M.-A. Langlois, S. Gulnik, A novel semiconductor-based flow cytometer with enhanced light-scatter sensitivity for the analysis of biological nanoparticles. Sci. Rep. 9, 16039 (2019). 31690751 10.1038/s41598-019-52366-4 PMC6831566

[53] L. Yang, S. Zhu, W. Hang, L. Wu, X. Yan, Development of an ultrasensitive dual-channel flow cytometer for the individual analysis of nanosized particles and biomolecules. Anal. Chem. 81, 2555–2563 (2009). 19260698 10.1021/ac802464a

[54] S. Zhu, L. Ma, S. Wang, C. Chen, W. Zhang, L. Yang, W. Hang, J. P. Nolan, L. Wu, X. Yan, Light-scattering detection below the level of single fluorescent molecules for high-resolution characterization of functional nanoparticles. ACS Nano 8, 10998–11006 (2014). 25300001 10.1021/nn505162u PMC4212780

[55] Y.-J. Li, J.-Y. Wu, J.-M. Wang, X.-B. Hu, D.-X. Xiang, Emerging strategies for labeling and tracking of extracellular vesicles. J. Control. Release 328, 141–159 (2020). 32882270 10.1016/j.jconrel.2020.08.056

[56] K. Takov, D. M. Yellon, S. M. Davidson, Confounding factors in vesicle uptake studies using fluorescent lipophilic membrane dyes. J. Extracell. Vesicles 6, 1388731 (2017). 29184625 10.1080/20013078.2017.1388731 PMC5699187

[57] M. P. Monopoli, A. Zendrini, D. Wu, S. Cheung, G. Sampedro, B. Ffrench, J. Nolan, O. Piskareva, R. L. Stalings, S. Ducoli, P. Bergese, D. F. O’Shea, Endogenous exosome labelling with an amphiphilic NIR-fluorescent probe. Chem. Commun. 54, 7219–7222 (2018). 10.1039/c8cc02135j 29900459

[58] C. A. Schneider, W. S. Rasband, K. W. Eliceiri, NIH Image to ImageJ: 25 years of image analysis. Nat. Methods 9, 671–675 (2012). 22930834 10.1038/nmeth.2089 PMC5554542

