# Supplementary material for: Water-soluble extracellular vesicle probes based on conjugated oligoelectrolytes
Source: Sci Adv. 2023 Jan 11;9(2):eade2996. doi: 10.1126/sciadv.ade2996 (PMC9833659; doi:10.1126/sciadv.ade2996)
Supplement: Supplementary file 1 — Figs. S1 to S94 Supplementary Measurements Synthesis Details [file sciadv.ade2996_sm.pdf]

Science Advances
AAAS

Supplementary Materials for

Water-soluble extracellular vesicle probes based on conjugated oligoelectrolytes

Cheng Zhou *et al.*

Corresponding author: Guillermo C. Bazan, chmbgc@nus.edu.sg

Sci. Adv. **9**, eade2996 (2023)
DOI: 10.1126/sciadv.ade2996

This PDF file includes:

Figs. S1 to S94
Supplementary Measurements
Synthesis Details

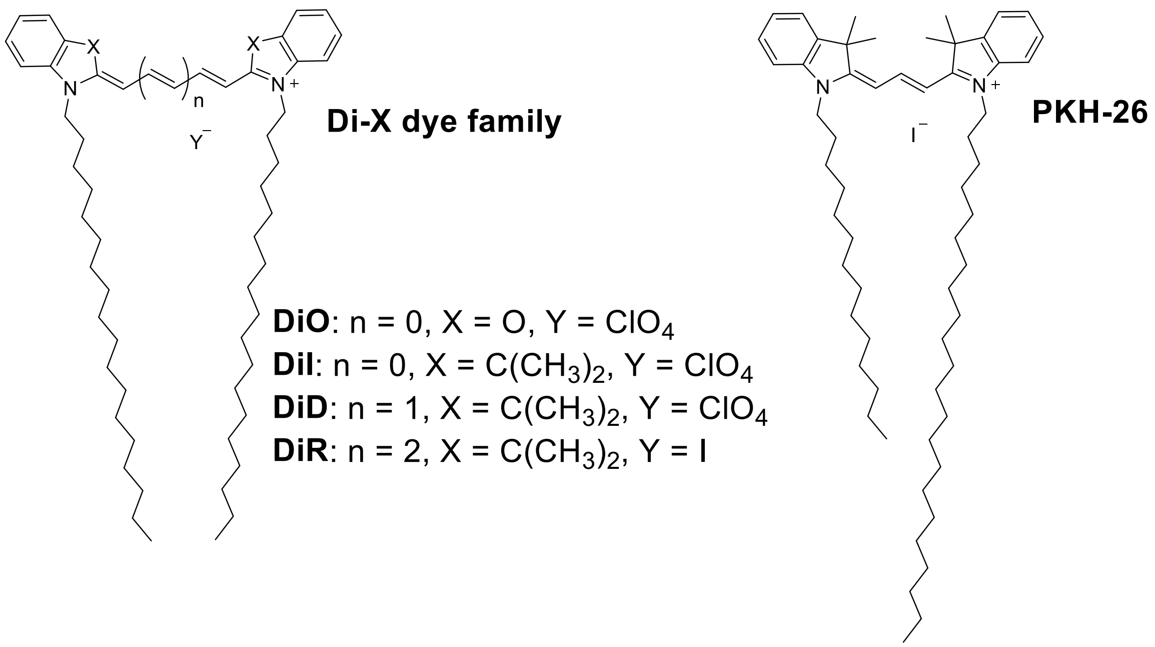

Fig. S1. Chemical structures. Chemical structures of lipophilic carbocyanine dyes.

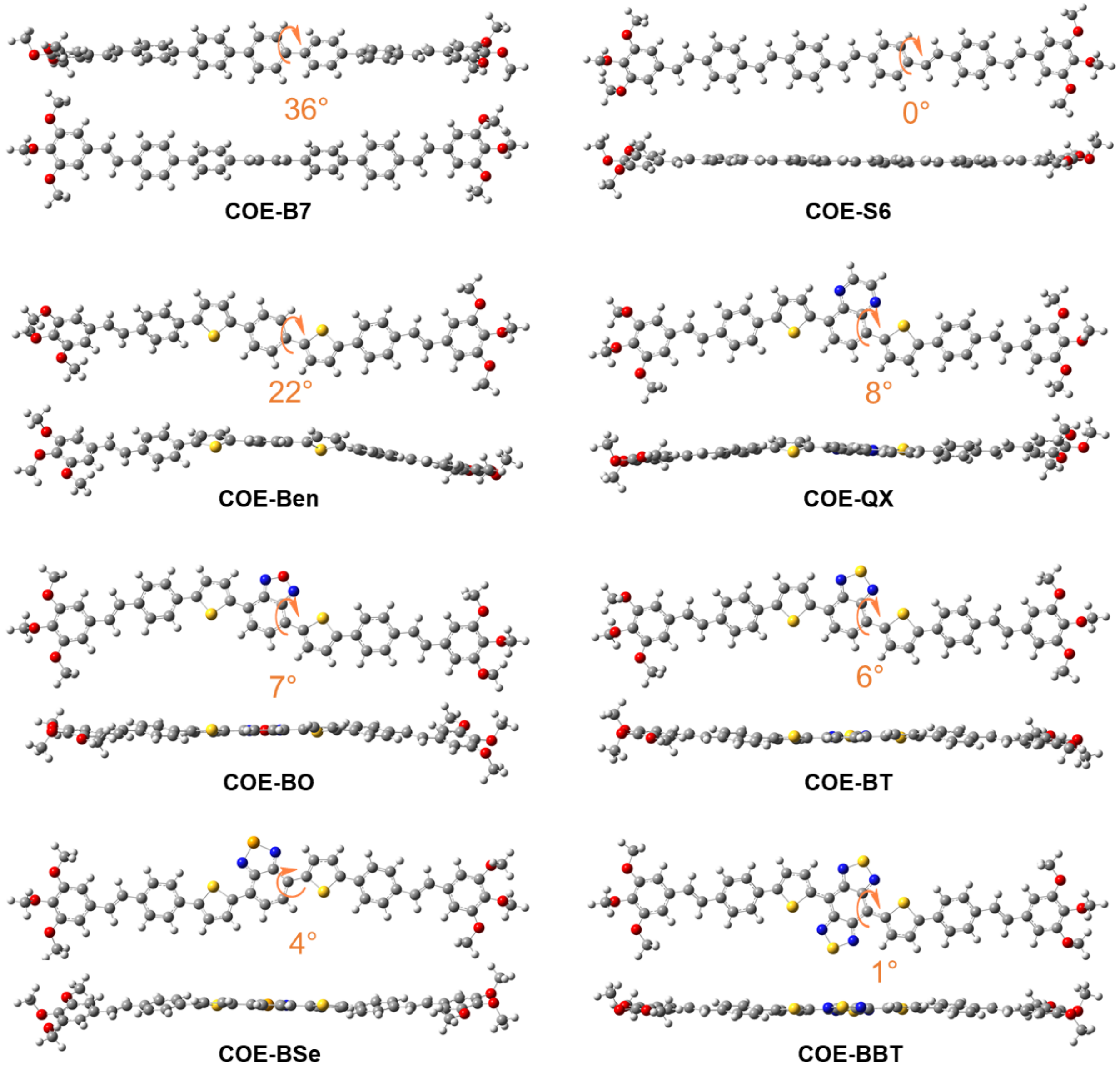

Fig. S2. Simulated COE backbone structures. DFT (density functional theory) optimized structure of all eight COE conjugated backbones using the B3LYP/6-31G(d,p) functional and basis set without solvation.

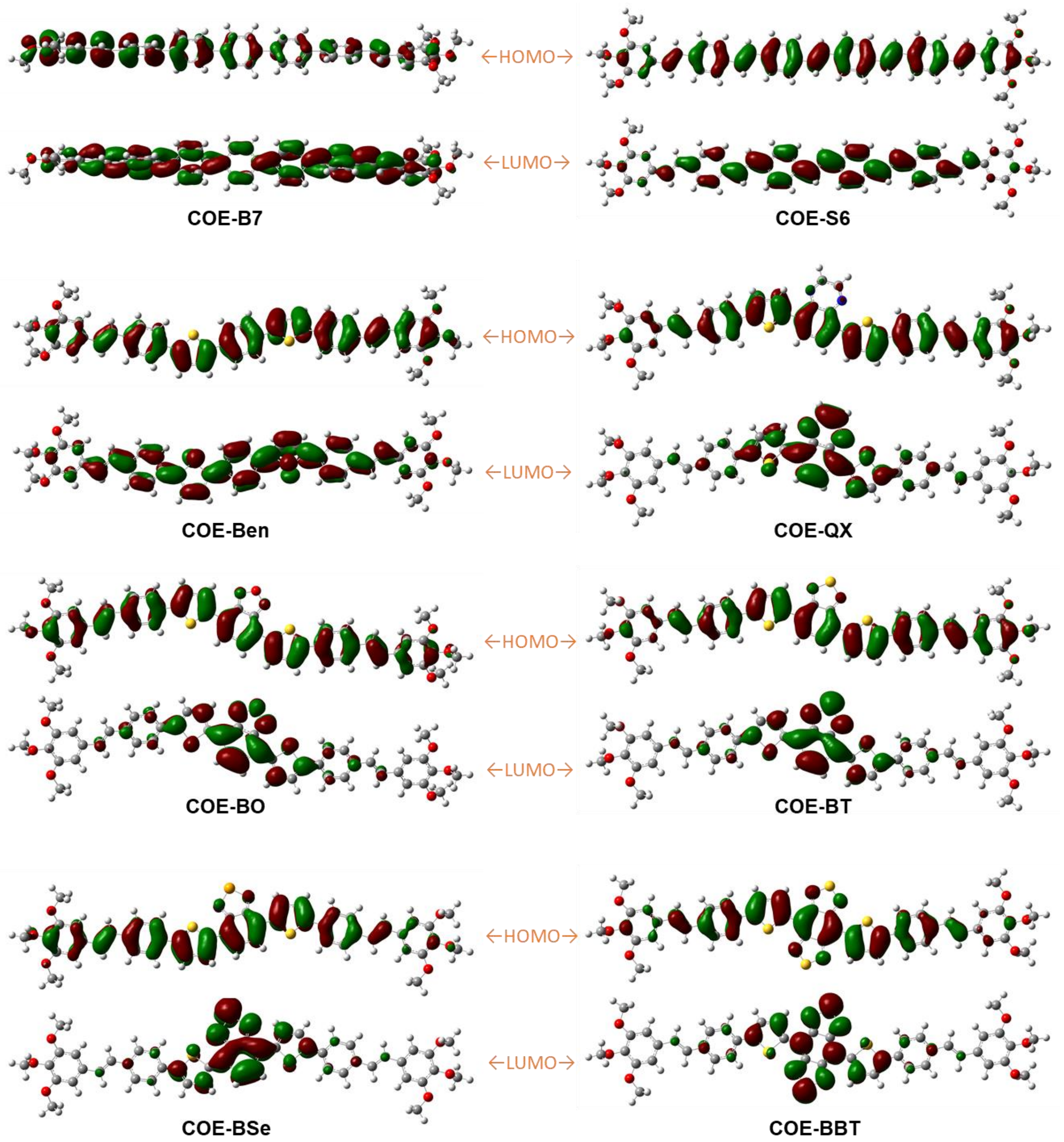

Fig. S3. Simulation of COE molecular orbitals. DFT optimized distribution of the HOMO (highest occupied molecular orbital) and LUMO (lowest unoccupied molecular orbital) along the eight COE conjugated backbones using the B3LYP/6-31G(d,p) functional and basis set without solvation.

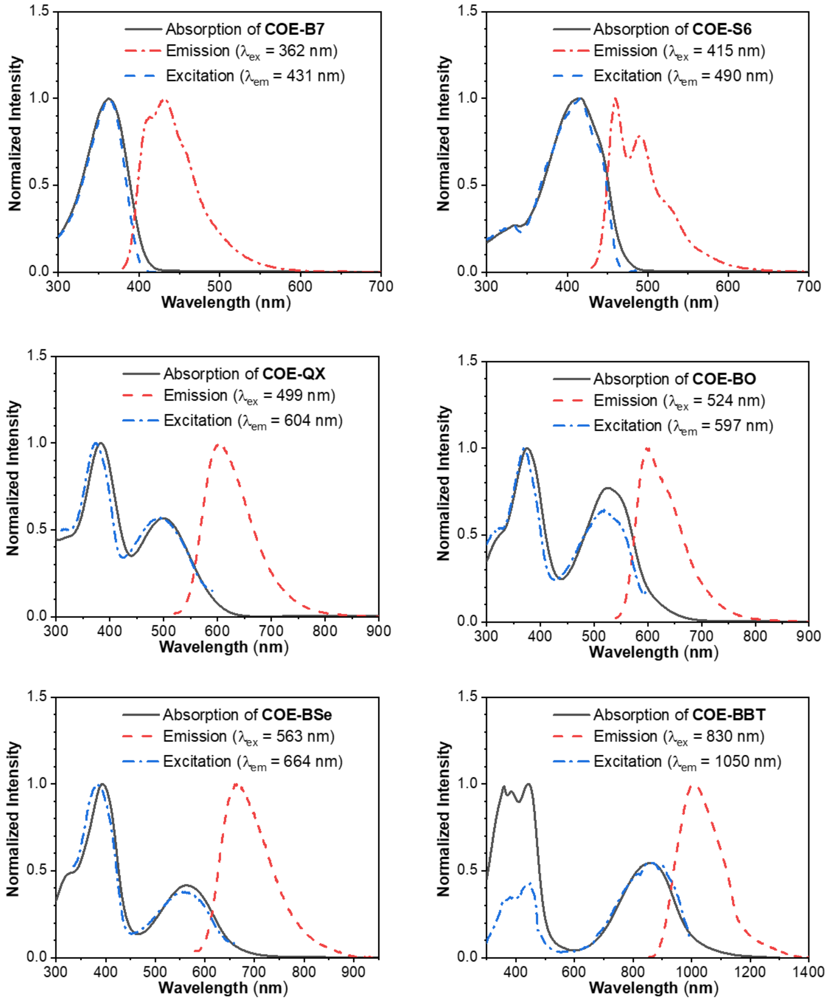

Fig. S4. Normalized optical spectra. Normalized whole absorption spectra of each COE in PBS, and normalized emission and excitation spectra of each COE after addition of 1 mM SUVs in PBS. In the absorption measurements, the COE concentration employed is 20 μM. In the emission and excitation measurements, the concentration for **COE-B7**, **COE-S6** and **COE-Ben** is 1 μM; for **COE-QX**, **COE-BO**, **COE-BT** and **COE-BSe** is 2 μM; for **COE-BBT** is 5 μM. The excitation wavelengths (λ_{ex}) for the emission measurements and emission detecting wavelengths (λ_{em}) are labeled in each figure. Specifically, these experiments here involved preheating mixtures of COEs and SUVs at 60 °C for 30 min to facilitate membrane intercalation.

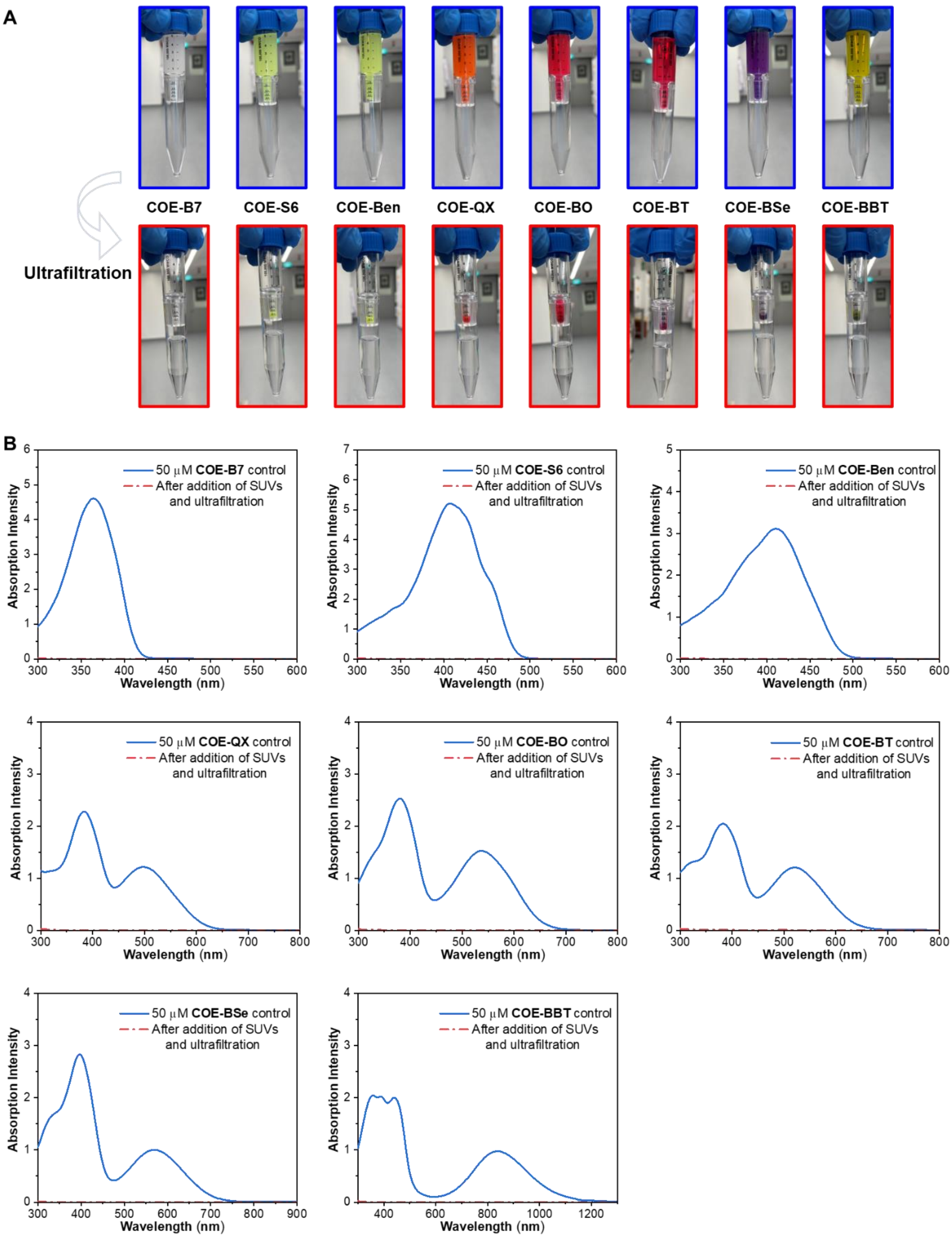

Fig. S5. Ultrafiltration experiments for COE-stained liposomes. (**A**) Photographs of the mixture of 50 µM COEs and 5 mM SUVs in PBS before and after ultrafiltration using 100K MWCO protein concentrator tubes (Pierce™, Thermo Scientific™) at 4000 relative centrifugal force. The lipid components of SUVs are POPC:POPG in a molar ratio of 85:15. The mixture of

COE-BT and SUVs in PBS was incubated at 60 °C for half hour before ultrafiltration to facilitate the membrane intercalation. (**B**) Absorption spectra of the filter liquor after ultrafiltration. 50 μM neat COE in PBS was used as the control.

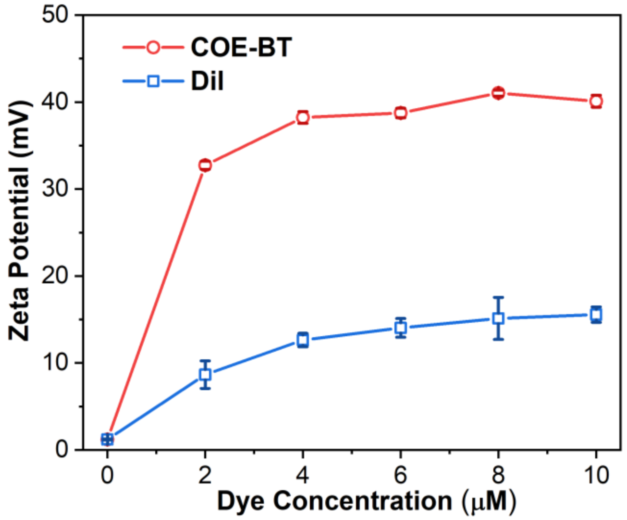

Fig. S6. Zeta potential measurements. Zeta potential curves for 1 mM POPC only SUVs stained by different concentrations of **COE-BT** or **DiI** in DI water.

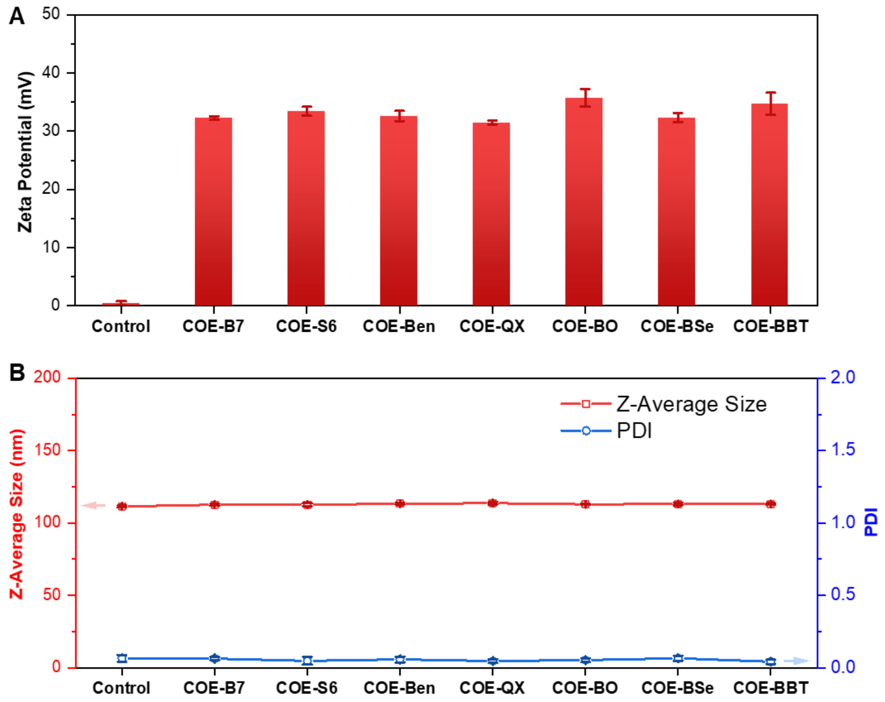

Fig. S7. Effects of COE treatments on liposomes. (**A**) Zeta potential measurements for 1 mM POPC only SUVs stained by 5 µM of different COEs in DI water. (**B**) DLS measured Z-Average size and PDI for 1 mM POPC only SUVs stained by 5 µM of different COEs in DI water.

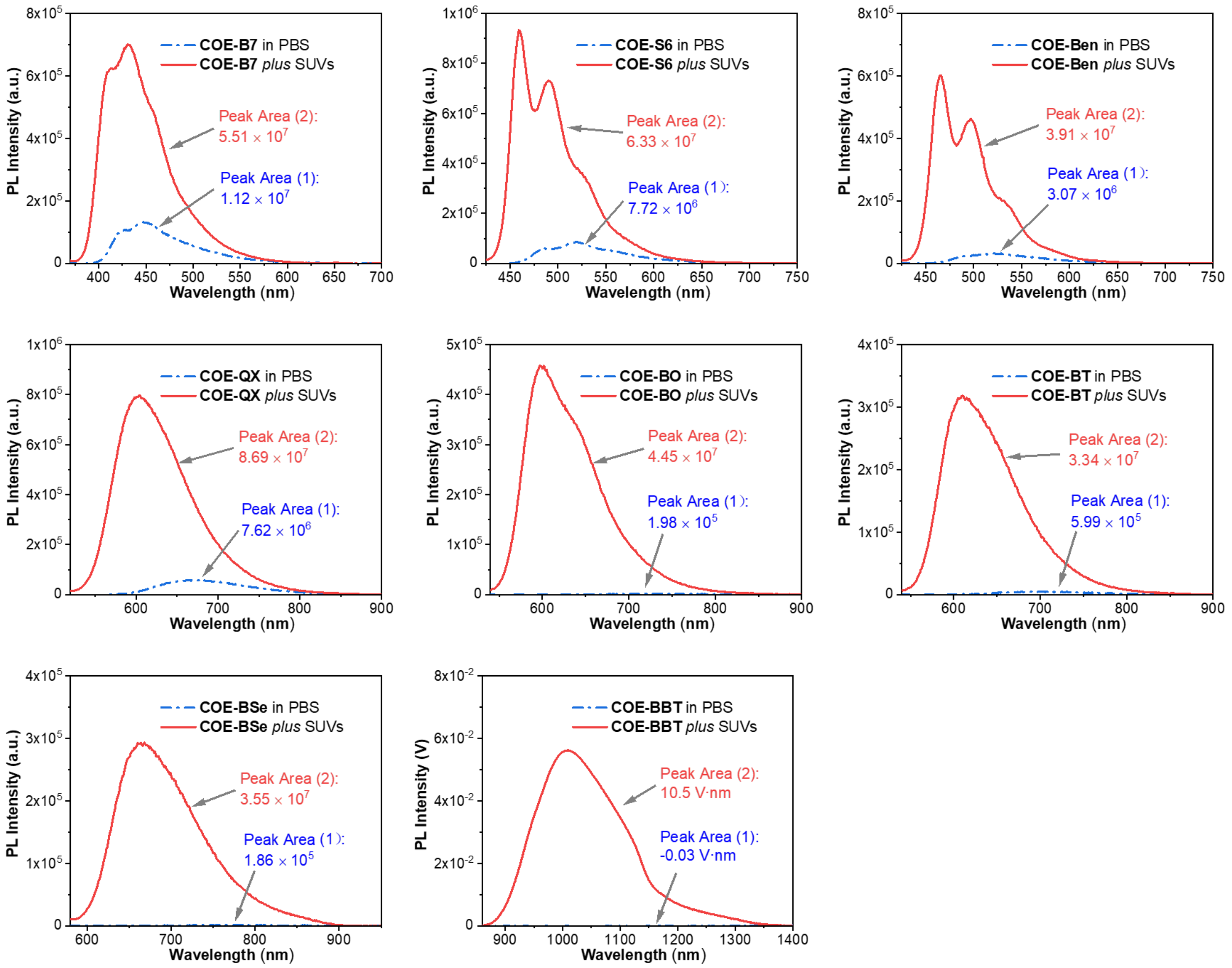

Fig. S8. Emission enhancement after membrane intercalation. Photoluminescence (PL) spectra of each COE without or with 1 mM SUVs addition in PBS. For the COE concentrations, **COE-B7**, **COE-S6** and **COE-Ben** are 1 µM, **COE-QX**, **COE-BO**, **COE-BT** and **COE-BSe** are 2 µM, **COE-BBT** is 5 µM. Within each figure, the COE concentration and measurement conditions are the same. To ensure effective membrane intercalation, the COE and SUV mixture was incubated at 60 °C for half hour.

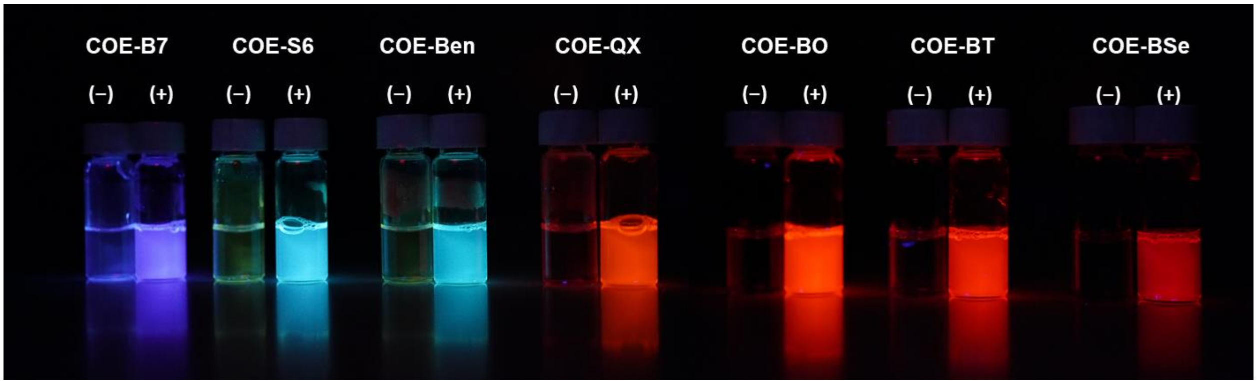

Fig. S9. Naked-eye detection. Photograph of COEs without (–) or with (+) 1 mM SUV treatment in PBS under UV-light (365 nm) exposure using a handheld UV lamp (UVP® UVLS-24 EL, 4 Watt). The concentration for **COE-B7** is 0.25 µM; for **COE-S6** and **COE-Ben** is 0.5 µM; for **COE-QX**, **COE-BO** and **COE-BT** is 1 µM; for **COE-BSe** is 5 µM. The camera (SONY, Alpha 7R) parameters are set to 8000 for ISO value, F2.8 for aperture, and 1/20 for exposure time.

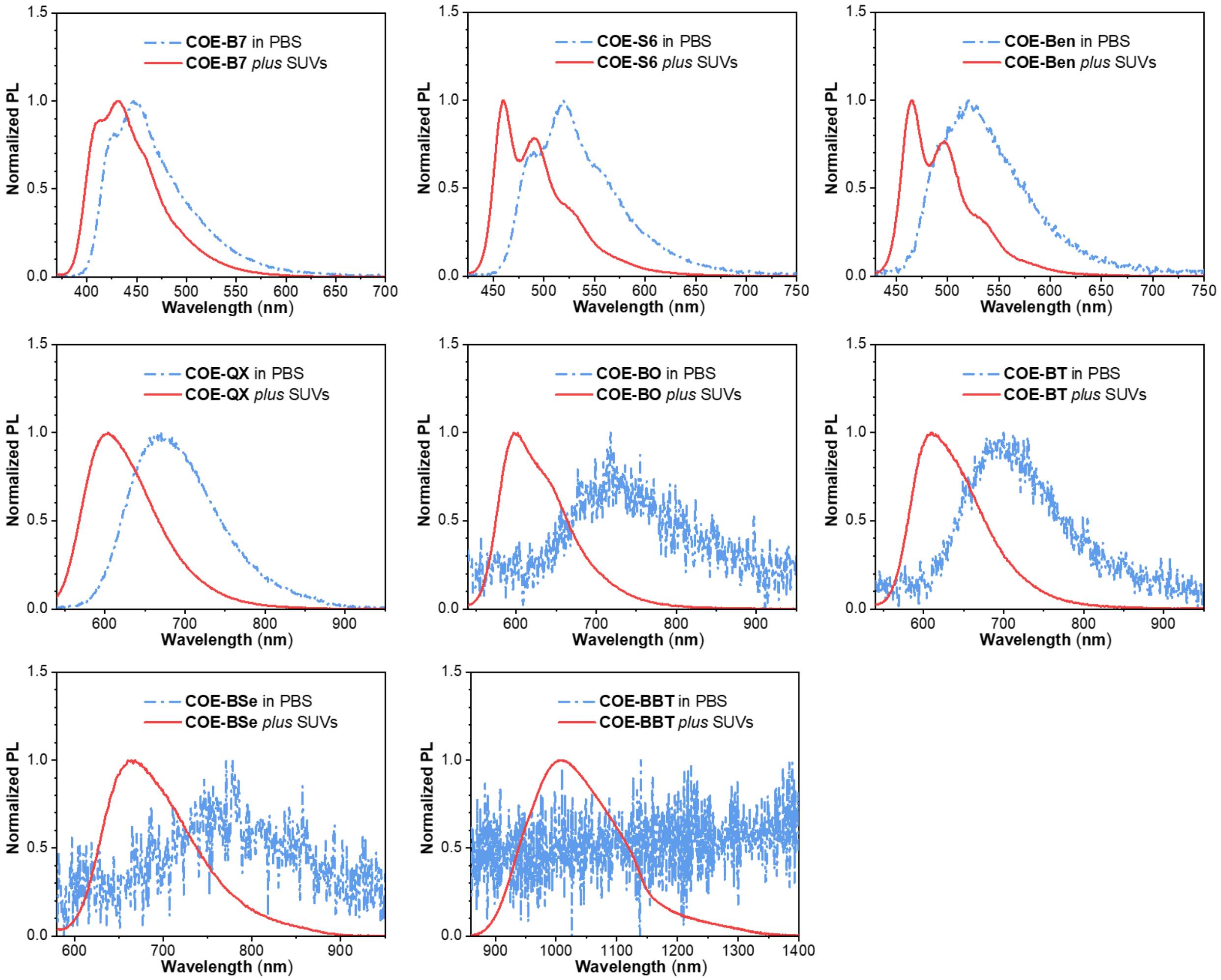

Fig. S10. Normalized PL spectra. Normalized photoluminescence (PL) spectra of COEs without or with 1 mM SUV addition in PBS. For the testing COE concentrations, **COE-B7**, **COE-S6** and **COE-Ben** are 1 µM, **COE-QX**, **COE-BO**, **COE-BT** and **COE-BSe** are 2 µM, **COE-BBT** is 5 µM. Within each figure, the COE concentration and measurement conditions are the same. To ensure effective membrane intercalation, the COE and SUV mixture was incubated at 60 °C for half hour.

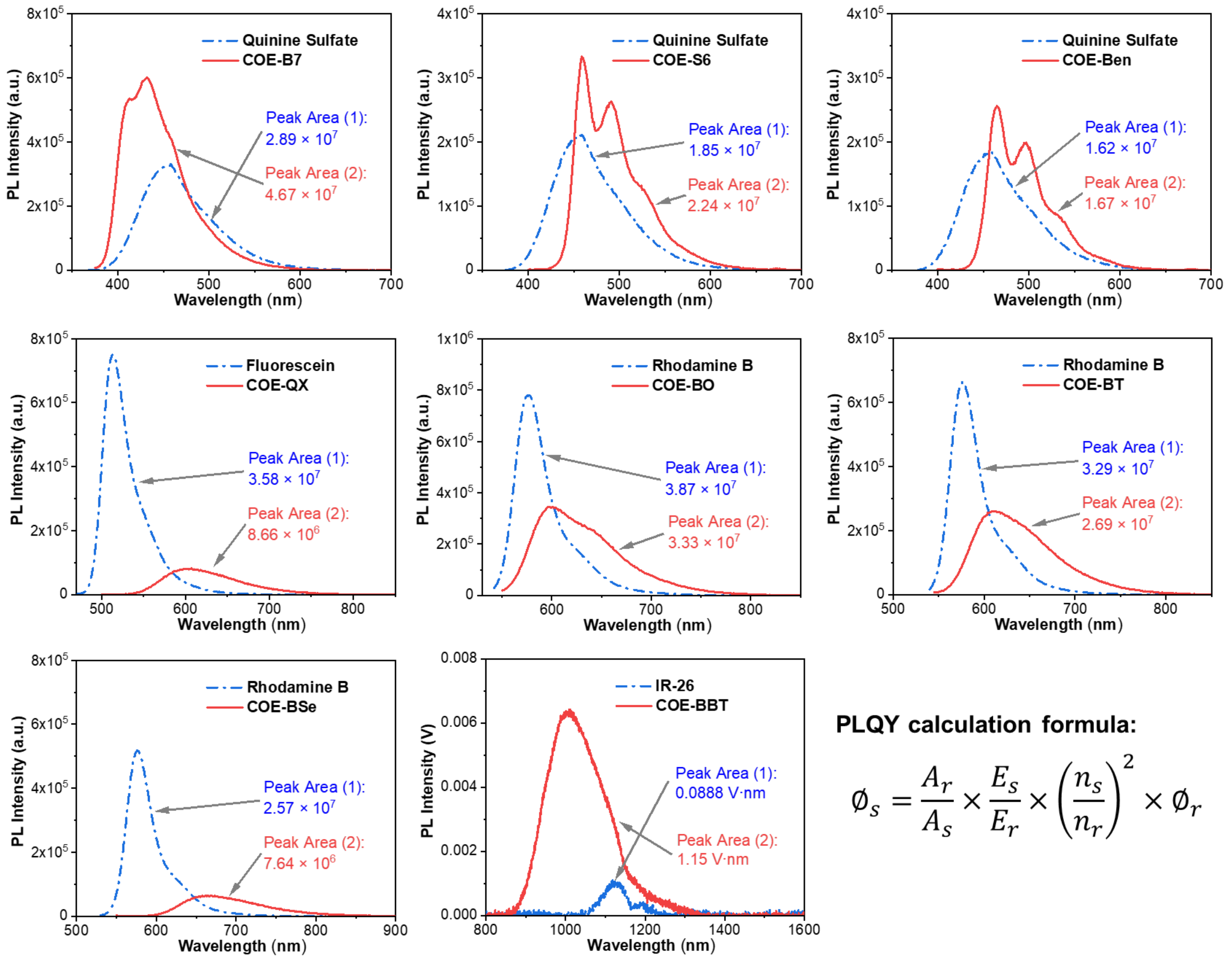

Fig. S11. PLQY measurements. Photoluminescence quantum yield (PLQY) measurements for different COEs after treated by 1 mM SUVs in PBS. The concentration of **COE-B7**, **COE-S6** and **COE-Ben** is 1 µM, the concentration of **COE-QX**, **COE-BO**, **COE-BT** and **COE-BSe** is 2 µM, the concentration of **COE-BBT** is 5 µM. To ensure effective membrane intercalation, the COE and SUV mixtures were incubated at 60 °C for half hour. The reference dye for **COE-B7**, **COE-S6** and **COE-Ben** is Quinine Sulfate in 0.1 M H_2SO_4 aqueous solution, whose quantum yield is 58%.(*45*) The reference dye for **COE-QX** is Fluorescein in 0.1 M NaOH aqueous solution, whose quantum yield is 95%. The reference dye for **COE-BO**, **COE-BT** and **COE-BSe** is Rhodamine B in water, whose quantum yield is 31%. The reference dye for **COE-BBT** is IR-26 in 1,2-dichloroethane, whose quantum yield is 0.05%.(*46*) The refractive index values for water and 1,2-dichloroethane is 1.333 and 1.4448, respectively. In each figure, the COE sample and reference dye were excited using the wavelength at which they have the same absorbance. In the formula that used to calculate the PLQY: Φ_s and Φ_r are the fluorescence quantum yields of testing sample and reference, respectively; A_s and A_r are the absorbance values of testing sample and reference, respectively; E_s and E_r are the integrated fluorescence intensity of testing sample and reference, respectively; n_s and n_r are the refractive index values of the solvents of testing sample and reference, respectively. The emission peak area was integrated using OriginPro 2021b.

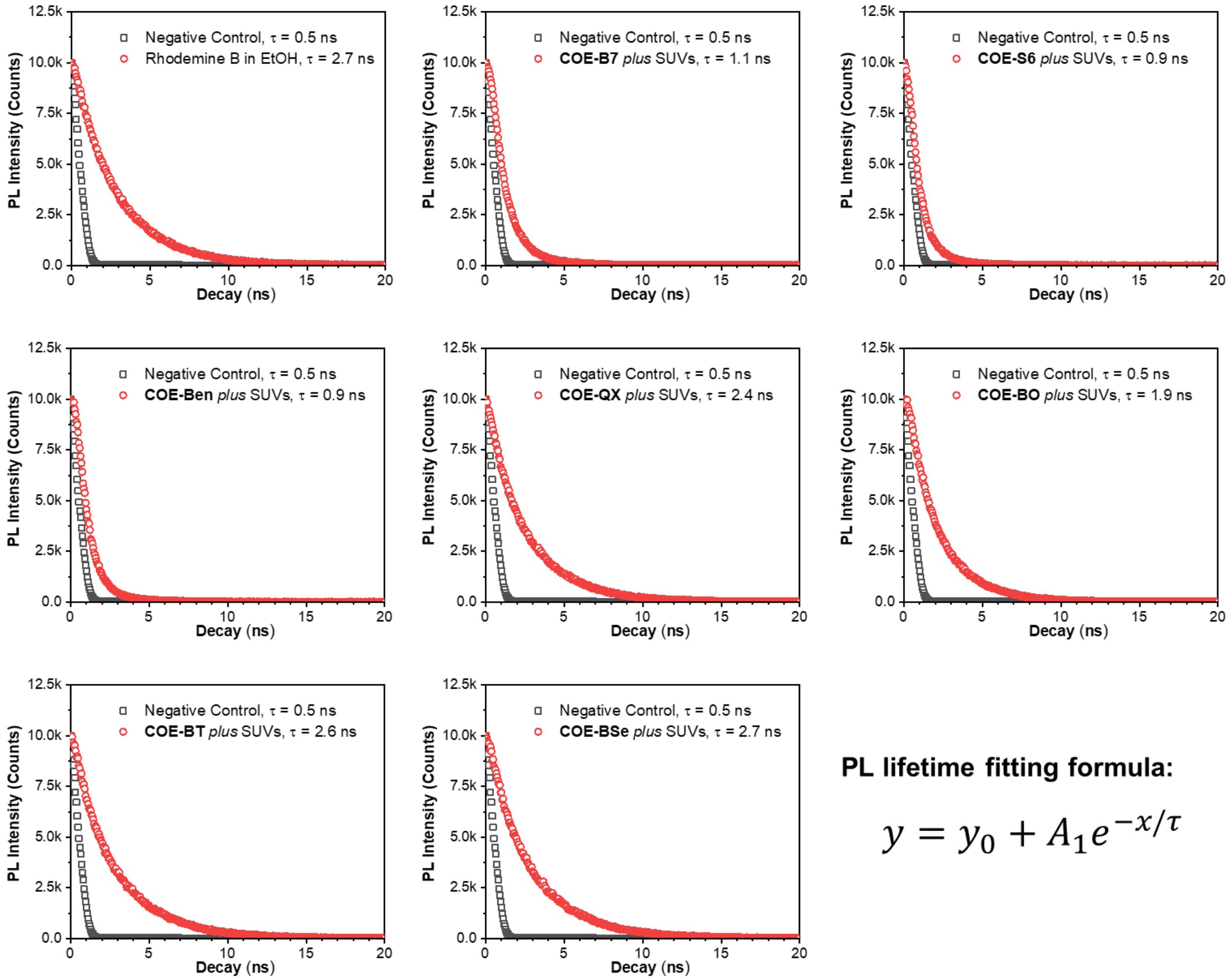

Fig. S12. PL lifetime measurements. PL lifetime measurements for different COEs after membrane intercalation in PBS. Blank PBS solution was used as the negative control. 2 μM Rhodamine B in water was used as the positive control by excitation using a 402 nm LED light source (DeltaDiode™ DD-405L) and collecting the emission at 570 nm (PL lifetime is 2.7 ns). The decay curve fitting was processed using OriginPro 2021b. In these experiments, the COE concentrations in PBS are 1 μM for **COE-B7**, **COE-S6** and **COE-Ben**, 2 μM for **COE-QX**, **COE-BO**, **COE-BT** and **COE-BSe**. The concentration of SUVs, composed of POPC:POPC in a molar ratio of 85:15, is 1 mM. All the samples above were excited using the 402 nm LED light source. The emission was detected at 431 nm for **COE-B7**, 490 nm for **COE-S6**, 497 nm for **COE-Ben**, 604 nm for **COE-QX**, 597 nm for **COE-BO**, 610 nm for **COE-BT**, 664 nm for **COE-BSe**.

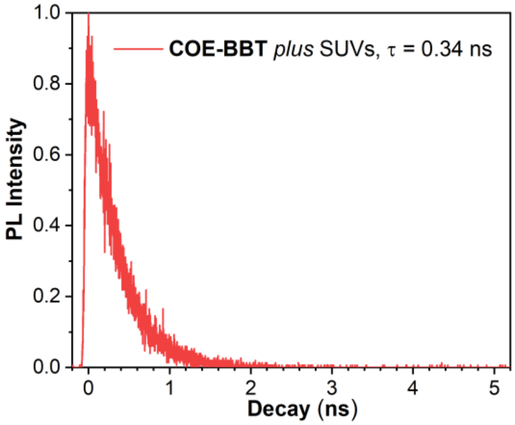

Fig. S13. PL lifetime measurement for COE-BBT. PL lifetime measurements for the mixture of 5 μM **COE-BBT** and 0.5 mg mL^{-1} SUVs (composed by POPC:POPC in a molar ratio of 85:15) in PBS. The sample was excited at 805 nm and the emission was collected at 980 nm.

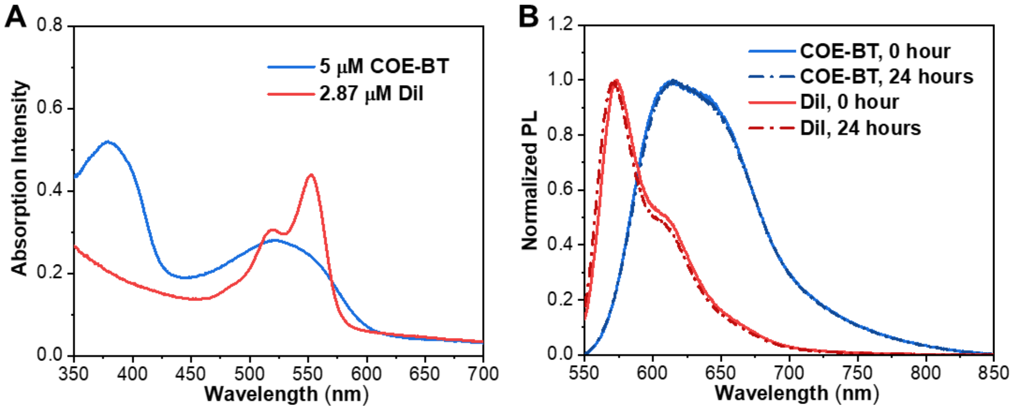

Fig. S14. Photostability measurements. Photostability evaluation for **COE-BT** and lipophilic carbocyanine dyes **DiI** with SUV addition under continuous exposure of 5 mW 532 nm laser over 24 hours. (**A**) Absorption spectra of dye with SUVs in PBS; (**B**) Normalized PL spectra before and after the 24-hour laser exposure.

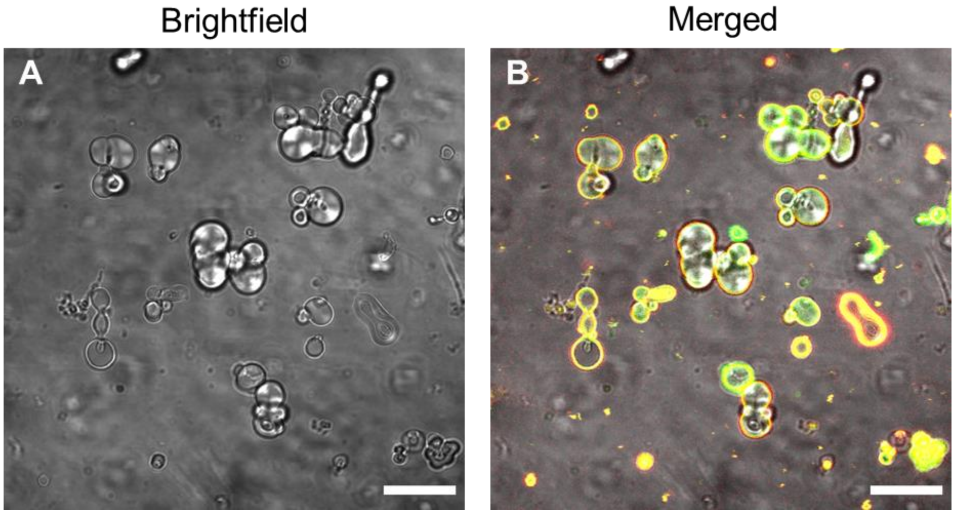

Fig. S15. Confocal microscopy measurements. Confocal micrographs of LMVs (large multilamellar vesicles) after stained by **COE-Ben** and **FM 4-64** in PBS. (**A**) Brightfield channel, (**B**) merged brightfield and fluorescent channels. The 6.25 mg mL^{-1} LMVs were stained by 15 µM **COE-Ben** and 15 µM **FM 4-64** in PBS for 30 minutes, and then diluted 5 times using PBS before confocal imaging. The **COE-Ben** fluorescent channel was observed by excitation at 405 nm and collecting the emission in the range of 450−490 nm (represented in green), and the **FM 4–64** fluorescent channel was observed by excitation at 561 nm and collecting the emission in the range of 640−700 nm (represented in red). The yellow patterns indicate the colocalization of **COE-Ben** and **FM 4-64**. The scale bars are 20 µm.

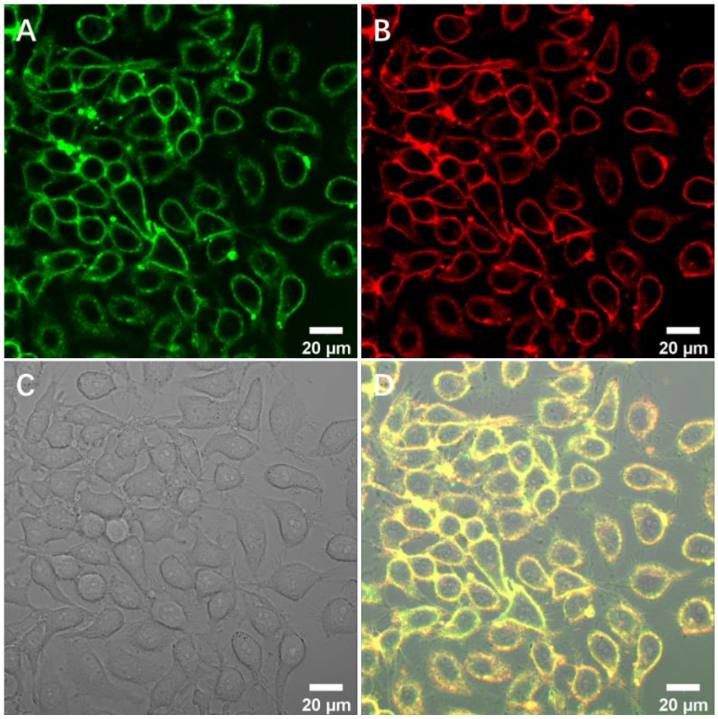

Fig. S16. Confocal micrographs of HepG2 cells. The cells were stained by 4 µM **COE-S6** and 4 µM commercially available membrane dye **FM 4-64**. (**A**) **COE-S6** channel, (**B**) **FM 4-64** channel, (**C**) brightfield channel, (**D**) merged channel. The **COE-S6** fluorescent channel was observed by excitation at 405 nm and collecting the emission in the range of 450−490 nm (green), and the **FM 4–64** fluorescent channel was observed by excitation at 561 nm and collecting the emission in the range of 640−700 nm (red). The scale bars are 20 µm.

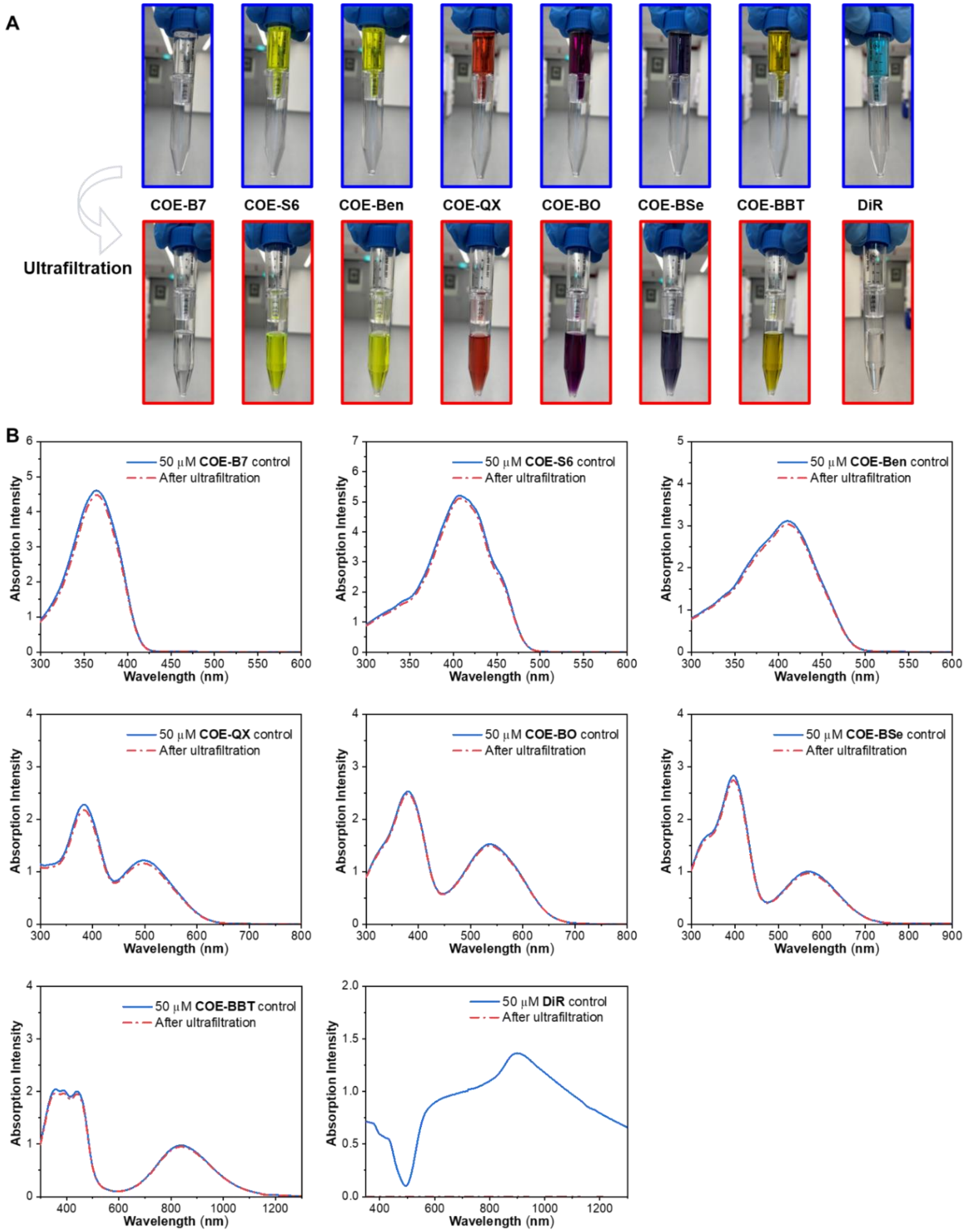

Fig. S17. Absence of nanoaggregate in COE aqueous solutions. (**A**) Photographs and (**B**) absorption spectra of 50 µM COEs or **DiR** solutions in PBS before and after ultrafiltration using 100K MWCO protein concentrator tubes (Pierce™, Thermo Scientific™) at 4000 relative centrifugal force. The slight reduction in the absorption spectra for the COE samples is probably due to the unspecific binding between the positively charged COEs and polyethersulfone-based ultrafiltration membrane.

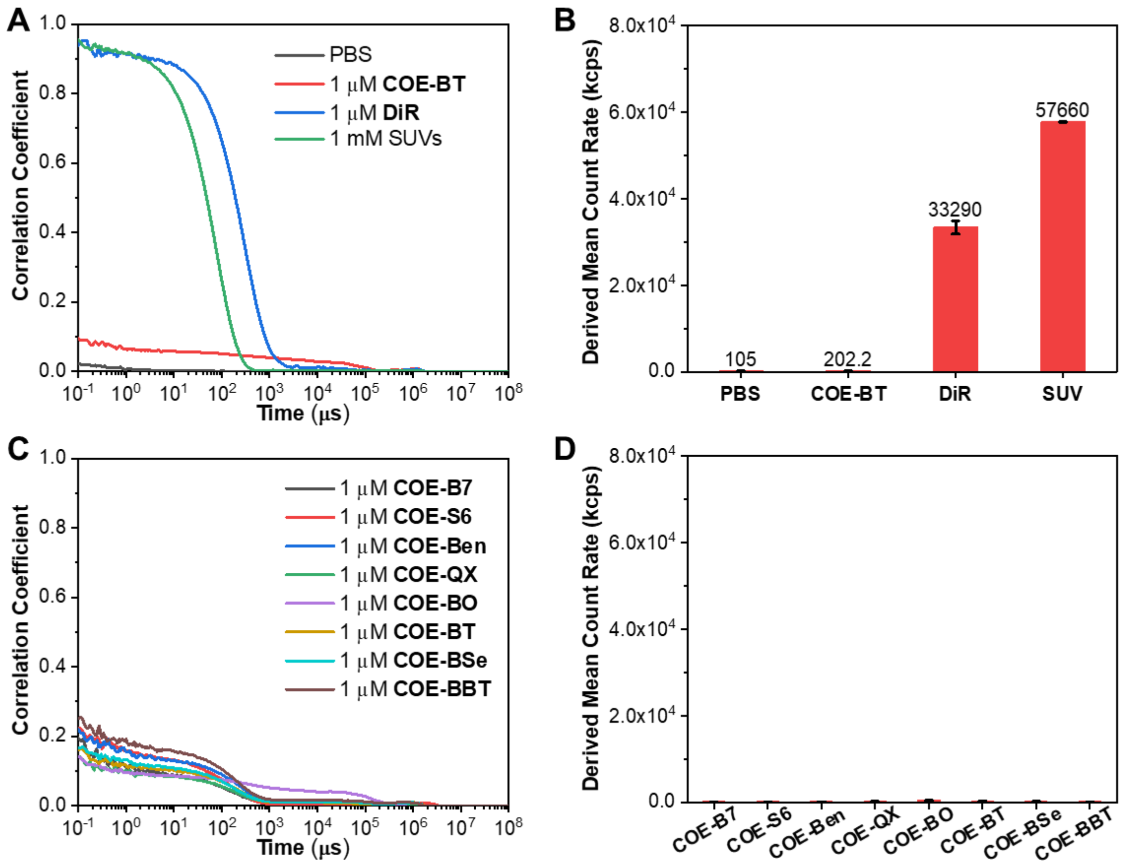

Fig. S18. DLS measurements. (**A**) Correlation coefficient curves of neat PBS, 1 µM **COE-BT** or 1 µM **DiR** or 1 mM SUVs in PBS as measured by dynamic light scattering (DLS). (**B**) DLS measured derived mean count rate of neat PBS, or 1 µM **COE-BT**, or 1 µM **DiR**, or 1 mM SUVs in PBS; the experiments were performed with five replicates. (**C**) Correlation coefficient curves of 1 µM other COEs in PBS as measured by DLS. (**D**) DLS measured derived mean count rate of 1 µM other COEs in PBS; the experiments were performed with five replicates.

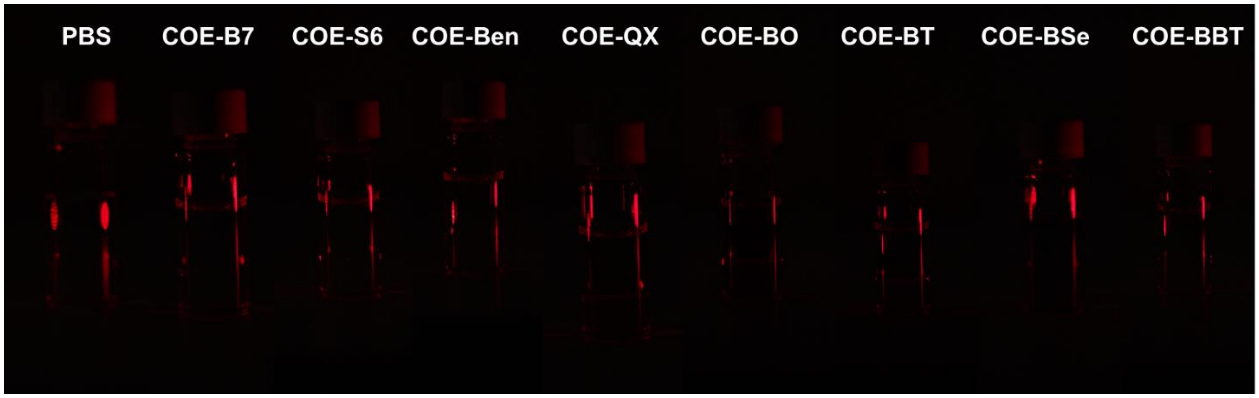

Fig. S19. Tyndall effect measurements. Photographs of Tyndall effects of neat PBS or 10 µM COEs in PBS after being illuminated using a red laser pointer.

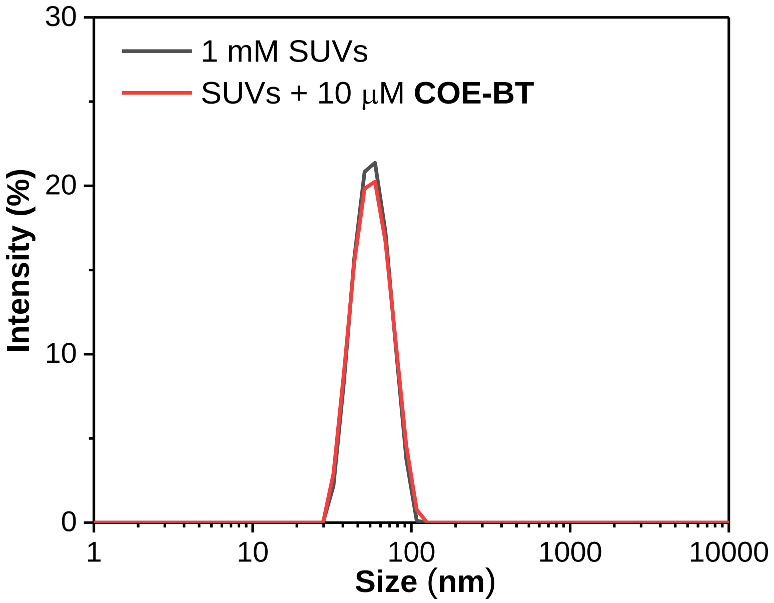

Fig. S20. Liposome size stability after COE treatment. Dynamic light scattering (DLS) measured size distribution curves by intensity of 1 mM SUVs without or with 10 μM **COE-BT** treatment in PBS. Different from other samples, the SUVs in the DLS measurements were obtained by extruding using a 30 nm membrane. The Z-average size and PdI (polydispersity index) values are 53.58 ± 0.53 nm and 0.058 ± 0.019 for sample "1 mM SUVs"; 54.00 ± 0.49 nm and 0.055 ± 0.027 for sample "SUVs + 10 μM **COE-BT**".

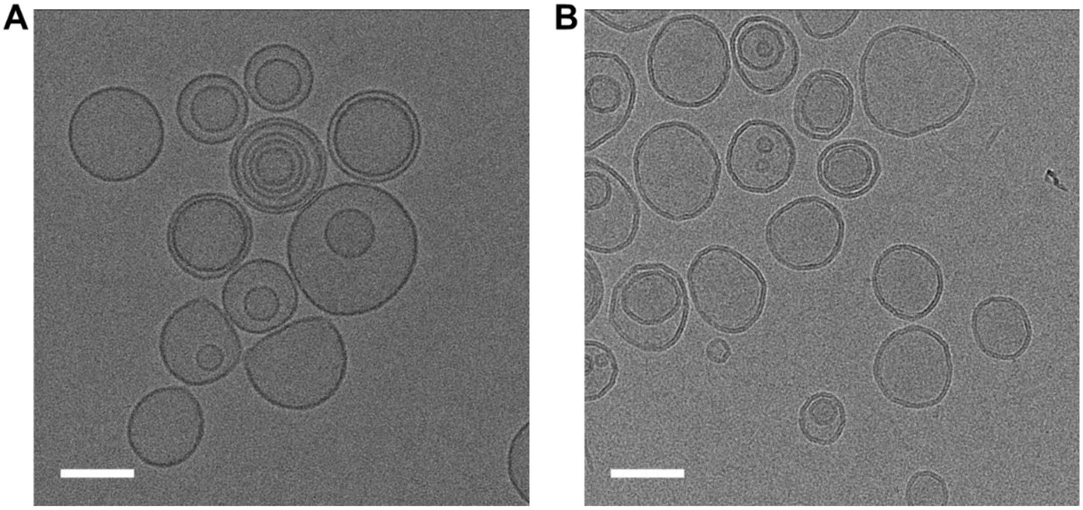

Fig. S21. Evaluating the impacts of COE on the morphology of SUV liposomes. Cryo-TEM images of 0.5 mg m L^{-1} POPC:POPG=85:15 SUV liposomes (**A**) without or (**B**) with 2 μM **COE-BT** incubation for 1 hour at room temperature. These SUVs were obtained by extruded using 200 nm membrane. Scale bars are 100 nm.

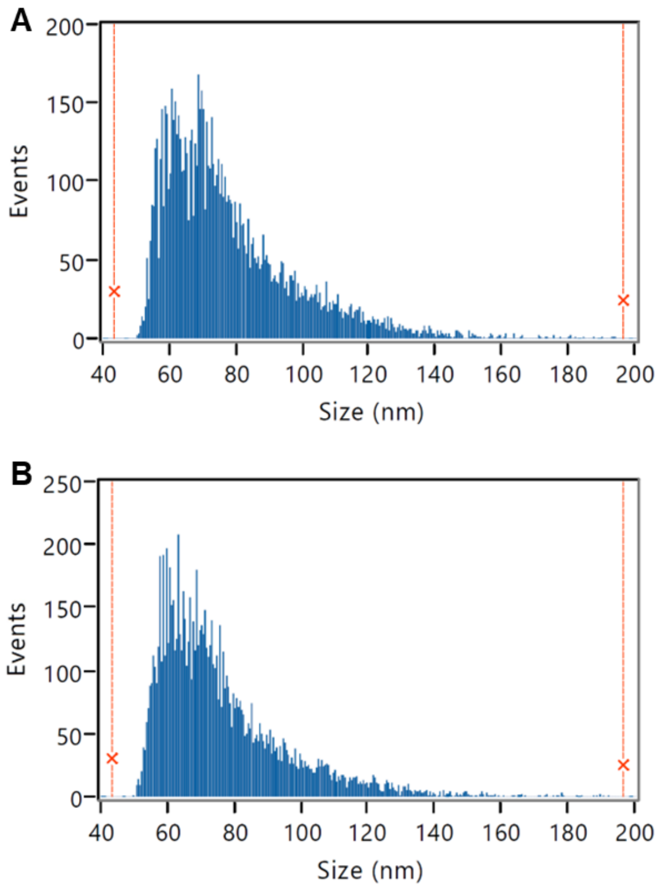

Fig. S22. Evaluating the impacts of COE on the size distribution of small EVs. Size distribution of SW480 small EVs in PBS (**A**) without or (**B**) with the addition of 1 μM **COE-BT**. Data were collected on NanoAnalyzer flow cytometer (NanoFCM).

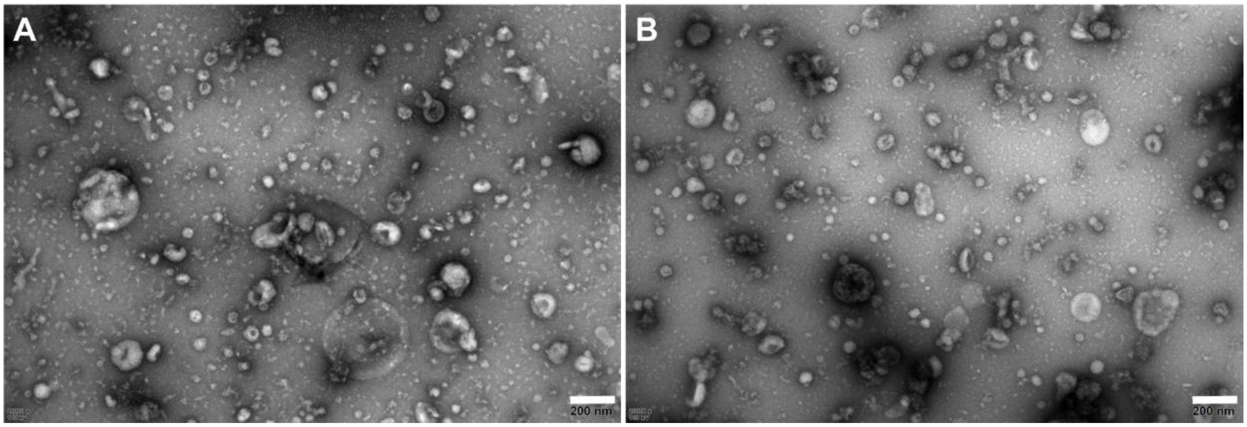

Fig. S23. Evaluating the impacts of COE on the morphology of small EVs. TEM images of 0.5 mg mL^{-1} PC-3 small EVs (**A**) without or (**B**) with 10 μM **COE-BT** treatment. Data were collected using Jeol JEM-1400 plus TEM. Typical cup-shaped morphology was observed due to the dehydration of small EVs during sample preparation. Scale bars are 200 nm.

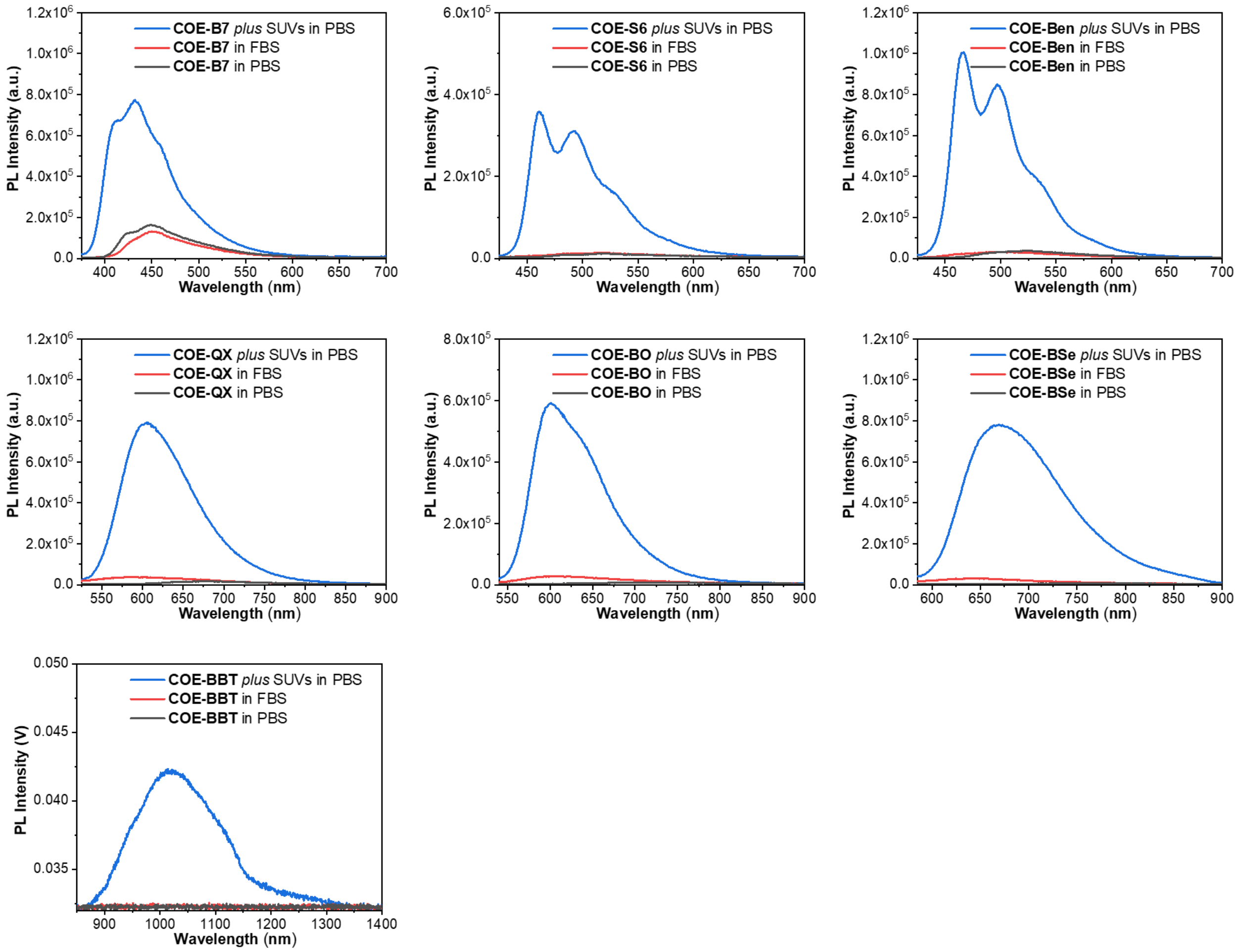

Fig. S24. Fluorescence response specificity of COEs. PL spectra of different COEs with 1 mg mL^{-1} SUVs treatment in PBS, or in FBS (fetal bovine serum), or in neat PBS. For the testing COE concentrations, **COE-B7**, **COE-S6** and **COE-Ben** are 1 μM, **COE-QX**, **COE-BO** and **COE-BSe** are 2 μM, **COE-BBT** is 5 μM. Within each figure, the COE concentration and measurement conditions are the same. The experiments were performed at room temperature.

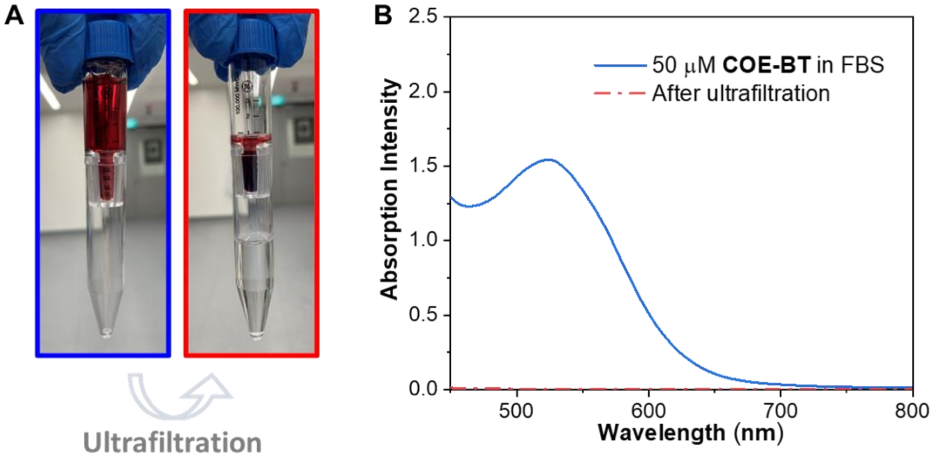

Fig. S25. Protein binding experiment. (**A**) Photographs of 50 μM **COE-BT** in FBS (fetal bovine serum) before and after ultrafiltration using 100K MWCO protein concentrator tubes (Pierce™, Thermo Scientific™) at 4000 relative centrifugal force. (**B**) Absorption spectra of the filter liquor after ultrafiltration. 50 μM **COE-BT** in PBS was used as the control.

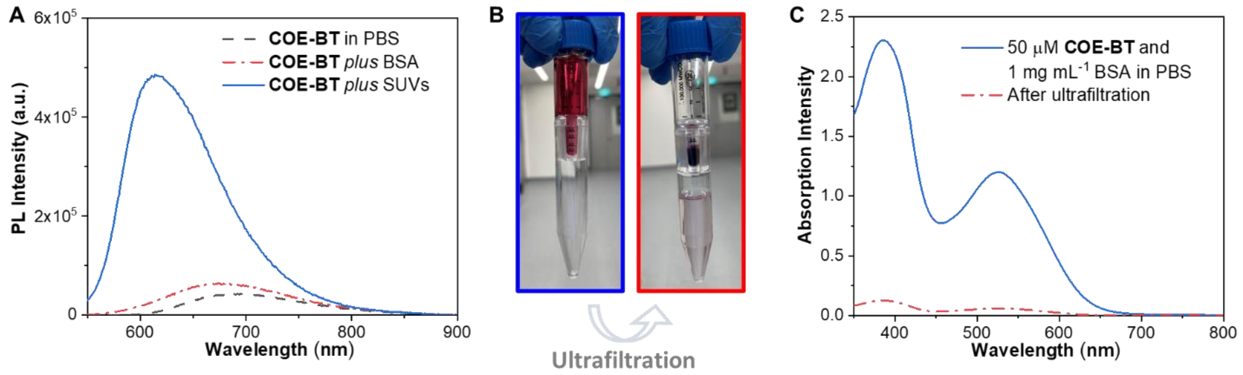

Fig. S26. Fluorescence response specificity of COE-BT. (**A**) PL spectra of 2 μM **COE-BT** in PBS, or with 1 mg mL^{-1} BSA (bovine serum albumin) in PBS, or with 1 mg mL^{-1} SUVs treatment in PBS. (**B**) Photographs of the mixture of 50 μM **COE-BT** and 1 mg mL^{-1} BSA in PBS before and after ultrafiltration using 100K MWCO protein concentrator tubes (Pierce™, Thermo Scientific™) at 4000 relative centrifugal force. (**C**) Absorption spectra of filter liquor after ultrafiltration. 50 μM **COE-BT** in PBS was used as the control.

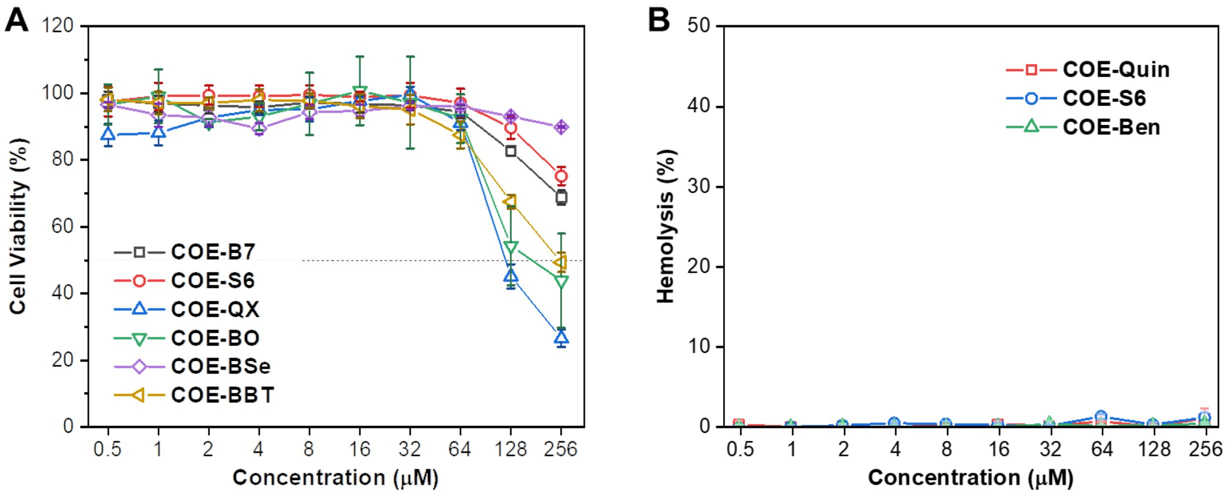

Fig. S27. *In vitro* toxicity studies. (**A**) Cytotoxicity measurements against A549 cells. The IC_{50} values for all six COEs are higher than 100 μM. (**B**) Hemolysis measurements against bovine erythrocytes in PBS.

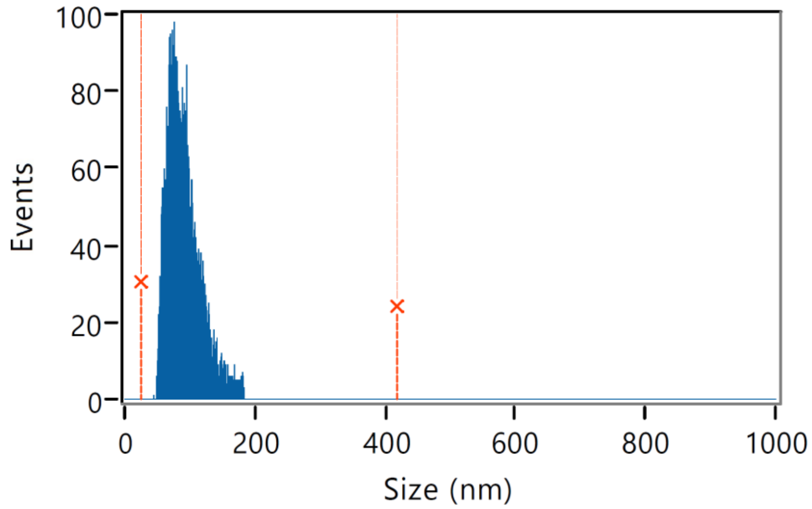

Fig. S28. Quality analysis for PC-3 "exosome" standards (ab239689). Size distribution of PC-3 small EVs (purchased from Abcam) was analyzed by NanoAnalyzer flow cytometer (NanoFCM) in PBS.

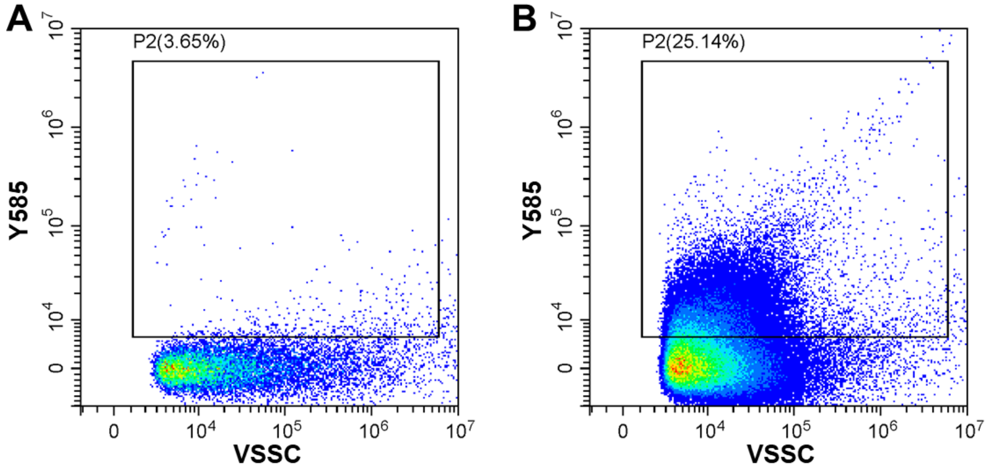

Fig. S29. Quality analysis for PC-3 "exosome" standards using surface markers. (**A**) Flow cytometry measurements for a cocktail of CD9-PE, CD63-PE, and CD81-PE antibodies as negative control. (**B**) 50 μL of 10 μg mL^{-1} PC-3 small EVs in PBS was mixed with 1 μL of each CD9-PE (clone HI9a), CD63-PE (clone H5C6), and CD81-PE (clone 5A6) that were purchased from Biolegend, and incubated at room temperature for 1 hour. These mixtures were diluted 100 times before flow cytometry experiments. The fluorescence of PE was detected by excitation at 561 nm and collecting emission at 585 nm (Y585 channel). The size was detected by the violet side scatter (VSSC) channel.

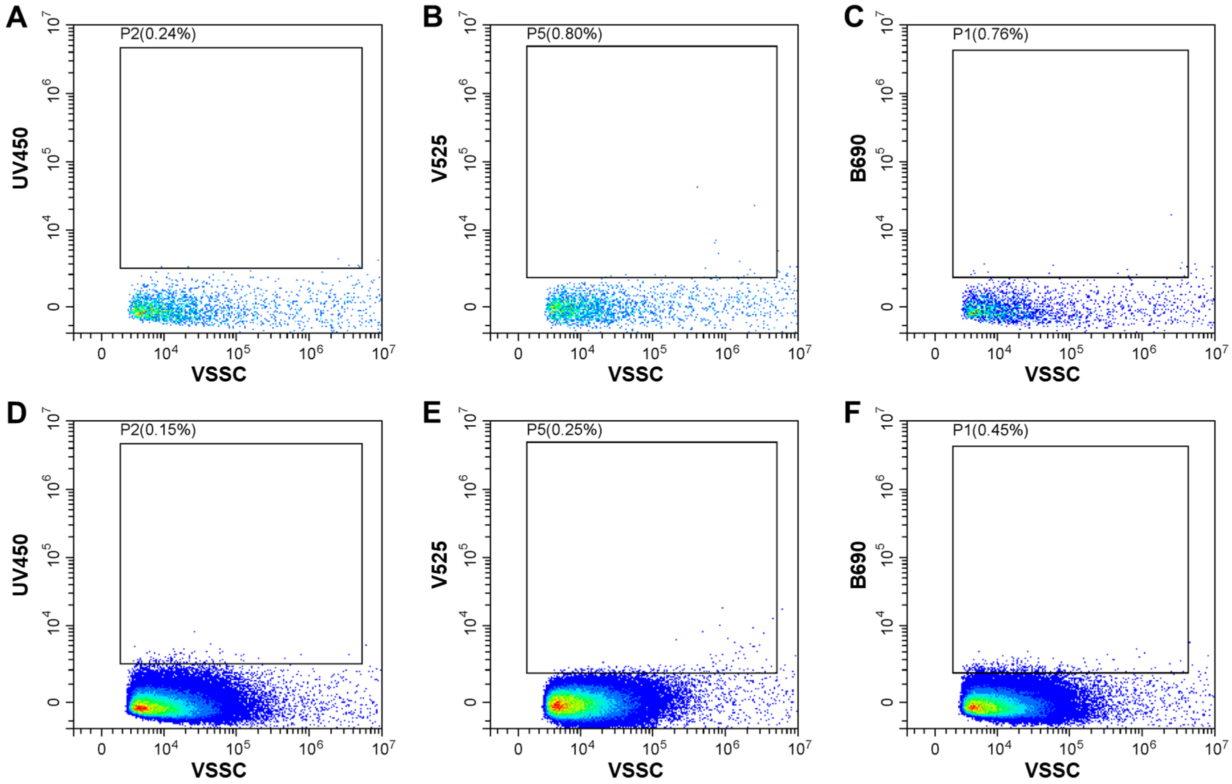

Fig. S30. Flow cytometry measurements for blank samples. Flow cytometry measurements for (**A-C**) neat PBS and (**D-F**) unstained PC3 small EVs in PBS. Data were collected on CytoFLEX LX flow cytometer (Beckman Coulter). The UV450 channel was detected by excitation at 355 nm and collecting emission at 450 nm. The V525 channel was detected by excitation at 405 nm and collecting emission at 525 nm. The B690 channel was detected by excitation at 488 nm and collecting emission at 690 nm. The size was detected by the violet side scatter (VSSC) channel. Data collection was terminated by controlling sample volume (10.0 µL).

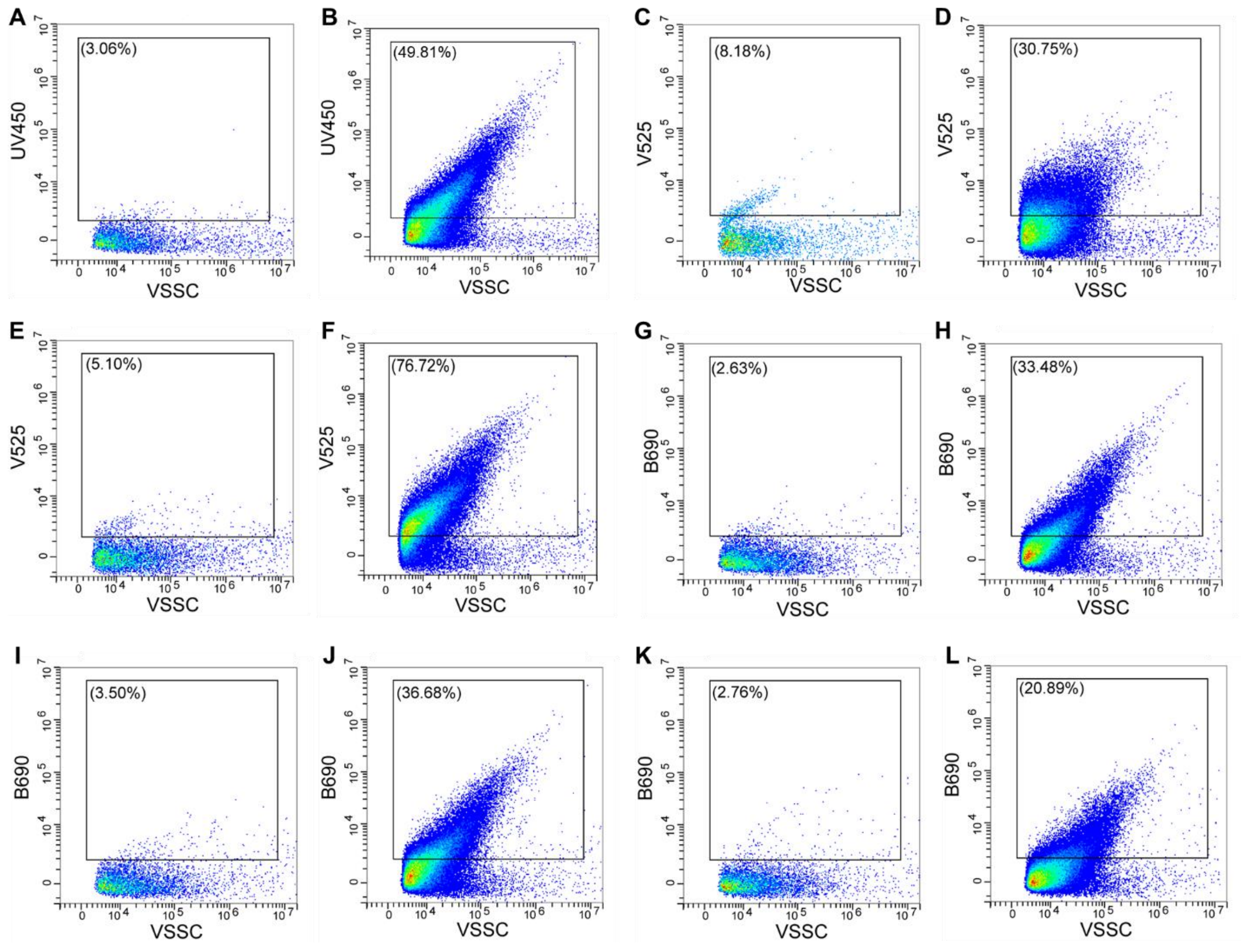

Fig. S31. Flow cytometry measurements for COE-stained small EVs. Flow cytometry measurements for COE controls and COE-stained PC3 small EVs in PBS. Data were collected on CytoFLEX LX flow cytometer (Beckman Coulter). (**A**) 0.5 µM **COE-B7** control; (**B**) 0.5 µM **COE-B7** stained small EVs; (**C**) 1 µM **COE-S6** control; (**D**) 1 µM **COE-S6** stained small EVs; (**E**) 0.5 µM **COE-Ben** control; (**E**) 0.5 µM **COE-Ben** stained small EVs; (**G**) 1 µM **COE-QX** control; (**H**) 1 µM **COE-QX** stained small EVs; (**I**) 1 µM **COE-BO** control; (**J**) 1 µM **COE-BO** stained small EVs; (**K**) 2.5 µM **COE-BSe** control; (**L**) 2.5 µM **COE-BSe** stained small EVs. The small EV mixture and controls were diluted 100 times using PBS before measurements. See procedure details in the Measurement section. The fluorescence of **COE-B7** was detected by excitation at 355 nm and collecting emission at 450 nm (UV450 channel). The fluorescence of **COE-S6** and **COE-Ben** was detected by excitation at 405 nm and collecting emission at 525 nm (V525 channel). The fluorescence of **COE-QX**, **COE-BO** and **COE-BSe** was detected by excitation at 488 nm and collecting emission at 690 nm (B690 channel). The size was detected by the violet side scatter (VSSC) channel. Data collection was terminated by controlling sample volume (10.0 µL).

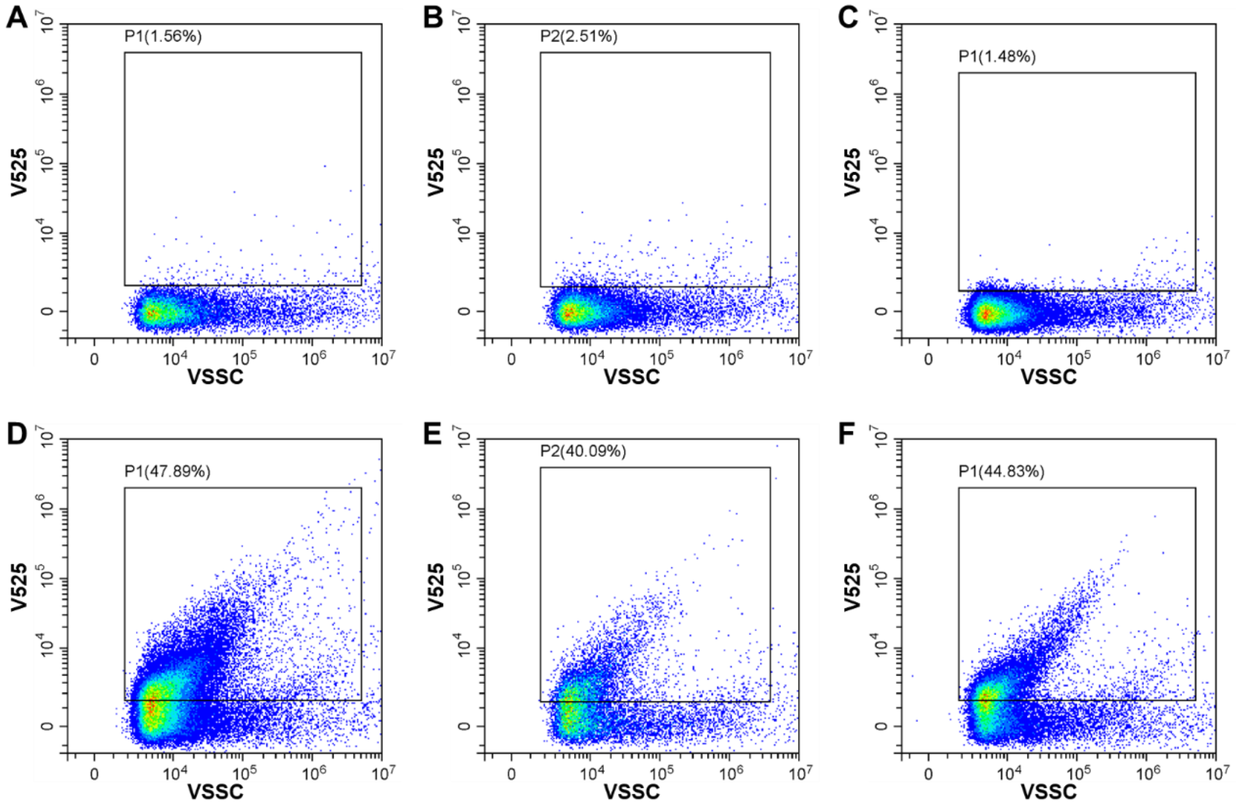

Fig. S32. Flow cytometry measurements for EVs isolated using different methods. Flow cytometry measurements for EVs (**A**-**C**) without or (**D**-**F**) with staining using 0.5 μM **COE-Ben** in PBS. (**A**, **D**) EVs secreted by A549 cells and isolated using ultracentrifugation, (**B**, **E**) EVs secreted by A549 cells and isolated using size-exclusion chromatography (SEC), (**C**, **F**) EVs secreted by HEK 293T cells and isolated using SEC. IZON qEV10 70 size exclusion columns were employed in SEC purifications. Data were collected on CytoFLEX LX flow cytometer (Beckman Coulter). The V525 channel was detected by excitation at 405 nm and collecting emission at 525 nm. The size was detected by the violet side scatter (VSSC) channel.

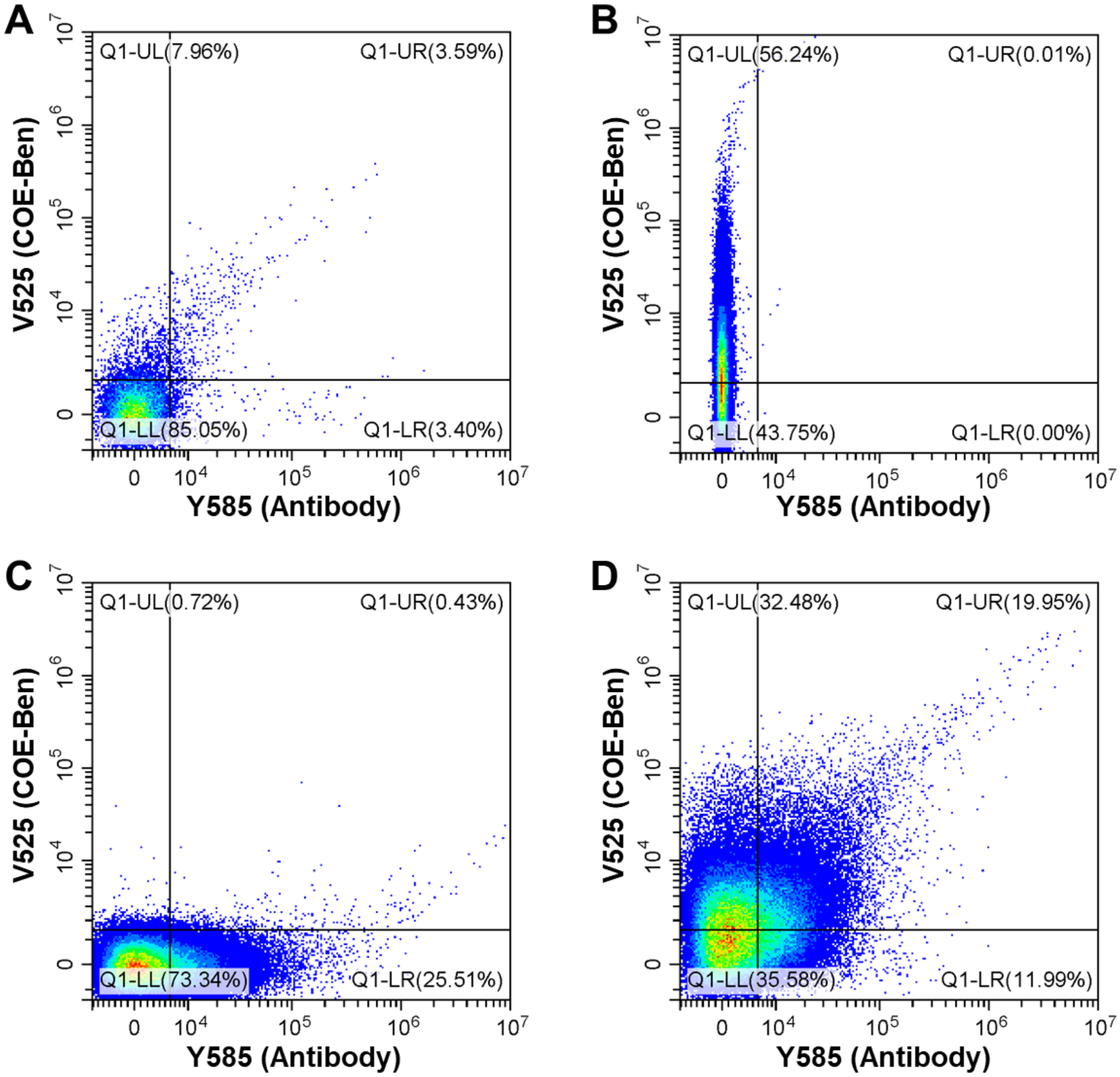

Fig. S33. Evaluating the impacts of COE on the binding of Antibody markers to small EVs. Flow cytometry measurements for (**A**) the mixture of **COE-Ben** and a cocktail of CD9-PE, CD63-PE, and CD81-PE antibodies to check the background; PC-3 small EVs stained by (**B**) **COE-Ben** or (**C**) the antibody cocktail. (**D**) PC-3 small EVs stained by the mixture of **COE-Ben** and antibody cocktail. 1 μL of each CD9-PE, CD63-PE, and CD81-PE (purchased from Biolegend), were mixed with 50 μL of 10 μg mL^{-1} PC-3 small EVs in PBS and incubated at room temperature for 1 hour then 5 μL of 5 μM **COE-Ben** was added and incubated at room temperature for 1 hour. These mixtures were diluted 100 times before flow cytometry experiments. The fluorescence of **COE-Ben** was detected by excitation at 405 nm and collecting emission at 525 nm (V525 channel). The fluorescence of PE (phycoerythrin) was detected by excitation at 561 nm and collecting emission at 585 nm (Y585 channel).

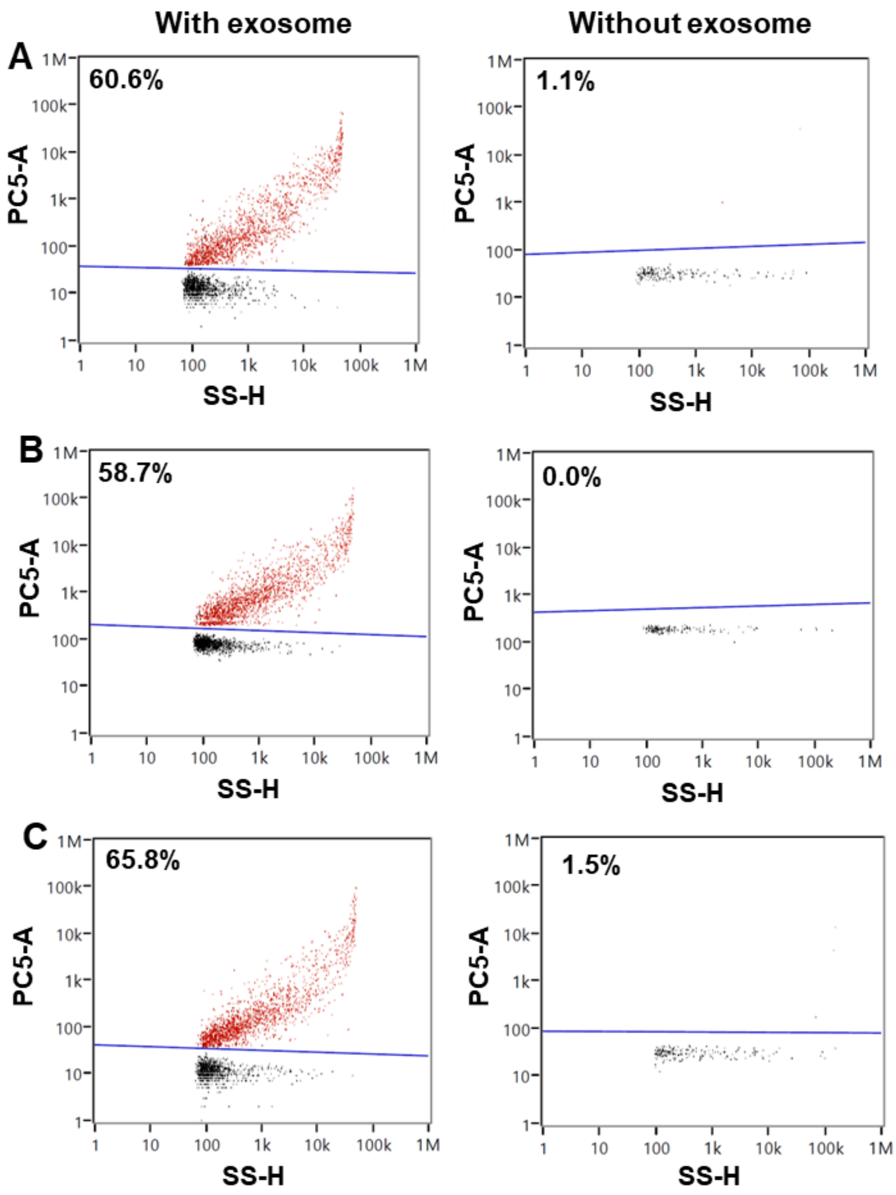

Fig. S34. Nano-flow cytometry measurements for COE-stained small EVs. Flow cytometry measurements for COE controls and COE-stained SW480 small EVs in PBS. Data were collected on NanoAnalyzer flow cytometer (NanoFCM). (**A**) 1 µM **COE-BO** with or without small EVs; (**B**) 1 µM **COE-QX** with or without small EVs; (**C**) 1 µM **COE-BSe** with or without small EVs. The small EV mixture and controls were diluted using PBS before measurements. See procedure details in the Measurement section. The fluorescence was detected by excitation at 488 nm and collecting emission at 670 nm (PC5 channel). The size was detected by the side scatter (SS) channel.

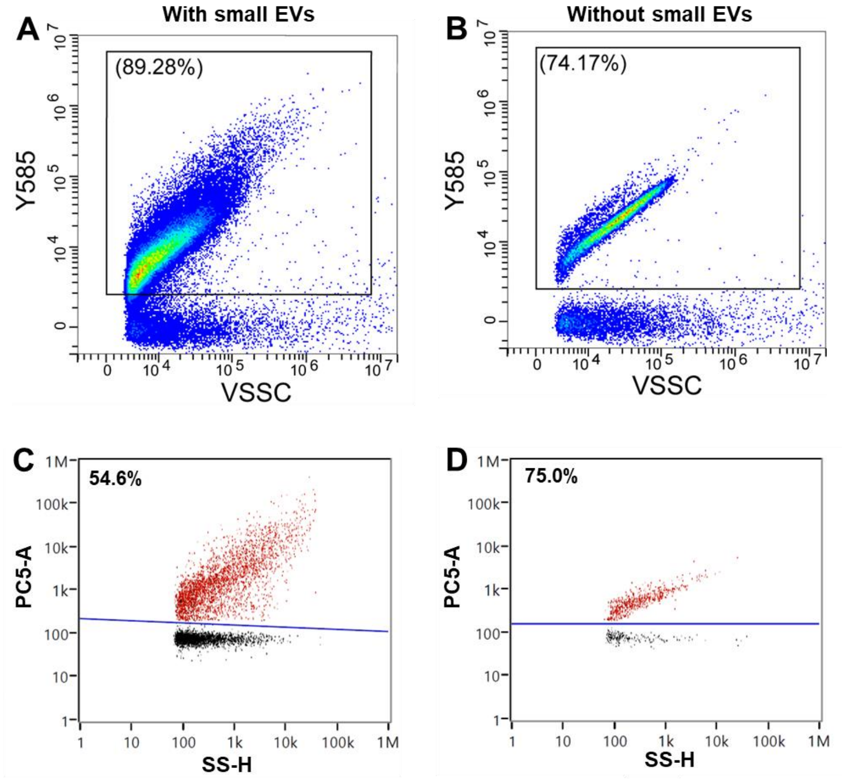

Fig. S35. Flow cytometry measurements of small EV labeled by PKH-26. Flow cytometry measurements for dye controls and stained small EVs in PBS. Data were collected on (**A**-**B**) CytoFLEX LX (Beckman Coulter) based on PC3 small EVs, or (**C**-**D**) NanoAnalyzer (NanoFCM) based on SW480 small EVs. (**A**) and (**C**) 1 µM **PKH-26** stained small EVs, (**B**) and (**D**) 1 µM **PKH-26** control. The small EV mixture and controls were diluted 100 times using PBS before measurements. The excitation and emission wavelengths are 561/585 nm for the Y585 channel and 488/670 nm for the PC5 channel, respectively. The size was detected by either violet side scatter (VSSC), or side scatter (SS) channel.

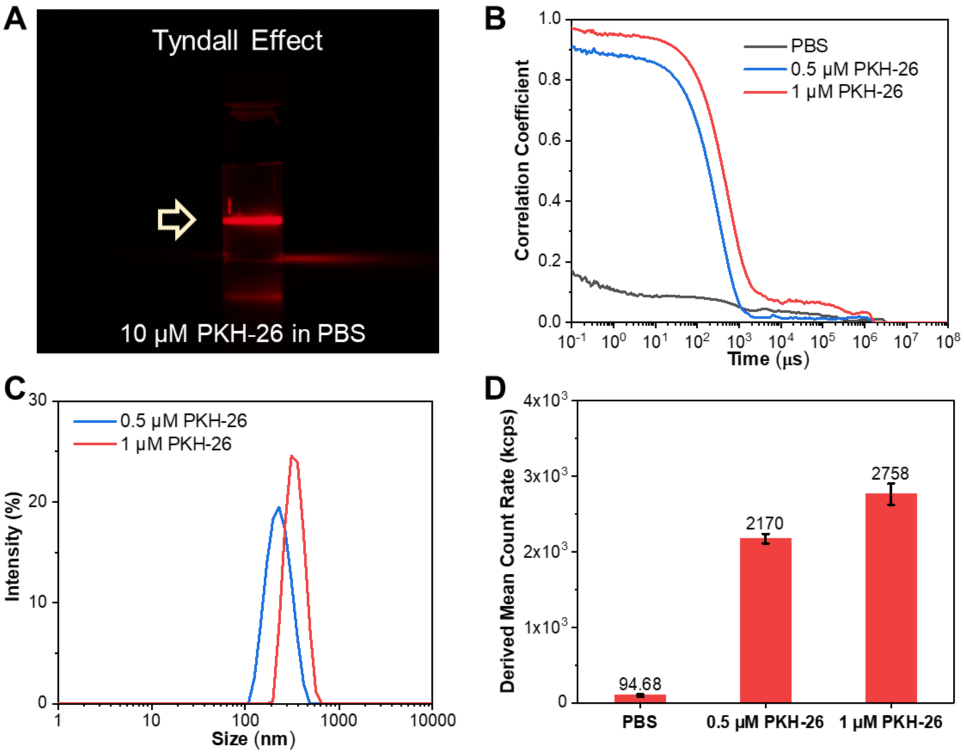

Fig. S36. Evaluating the aggregation for PKH-26 in aqueous solution. (**A**) Photographs of Tyndall effect of 10 μM **PKH-26** in PBS after being illuminated using a red laser pointer. (**B**) Correlation coefficient curves of 0.5 or 1 μM **PKH-26** in PBS as measured by DLS. (**C**) DLS measured size distribution curves by intensity of 0.5 or 1 μM other **PKH-26** in PBS. (**D**) DLS measured derived mean count rate of 0.5 or 1 μM other **PKH-26** in PBS; the DLS experiments were performed with five replicates.

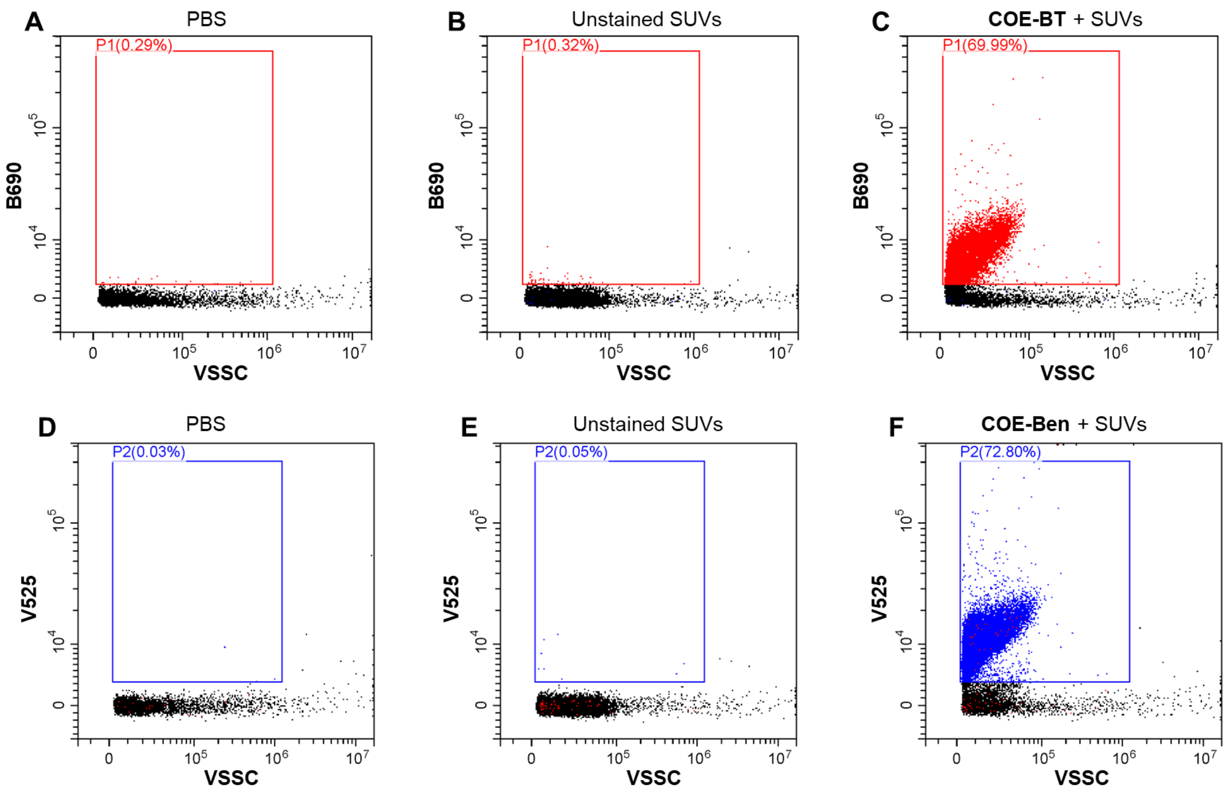

Fig. S37. Flow cytometry measurements of model liposomes. Flow cytometry measurements for (**A**) neat PBS, (**B**) unstained SUVs, (**C**) SUVs stained by **COE-BT**, (**D**) neat PBS, (**E**) unstained SUVs, and (**F**) SUVs stained by **COE-Ben** in PBS. Data were collected on CytoFLEX LX flow cytometer (Beckman Coulter). The fluorescence of **COE-BT** was detected by excitation at 488 nm and collecting emission at 690 nm (B690 channel). The fluorescence of **COE-Ben** was detected by excitation at 405 nm and collecting emission at 525 nm (V525 channel). The size was detected by the violet side scatter (VSSC) channel.

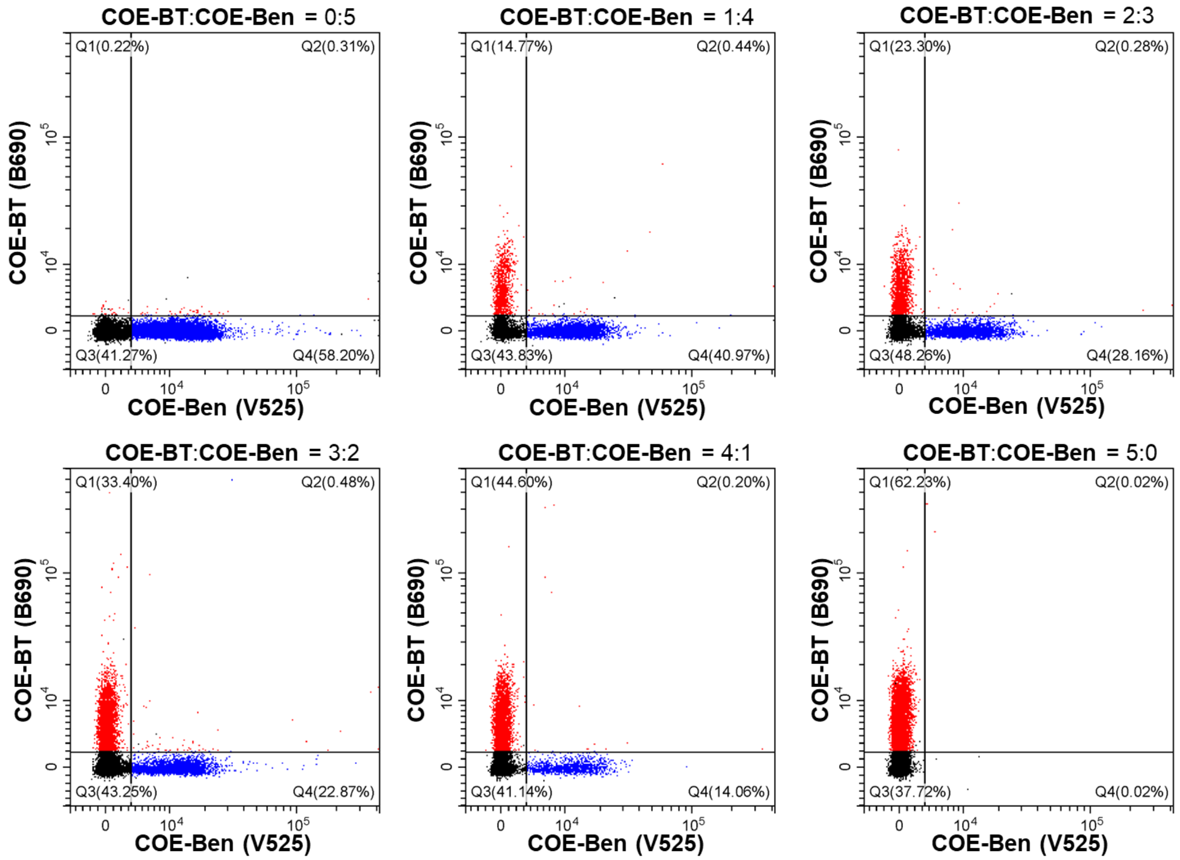

Fig. S38. Binding stability of COE after membrane intercalation. Flow cytometry measurements for mixtures of **COE-BT**-stained and **COE-Ben**-stained SUVs with different ratios in PBS after mixing for 24 hours. Data were collected on CytoFLEX LX flow cytometer (Beckman Coulter). The fluorescence of **COE-BT** was detected by excitation at 488 nm and collecting emission at 690 nm (B690 channel). The fluorescence of **COE-Ben** was detected by excitation at 405 nm and collecting emission at 525 nm (V525 channel). The size was detected by the violet side scatter (VSSC) channel. Around 50,000 events were shown in each two-dimensional dot plot.

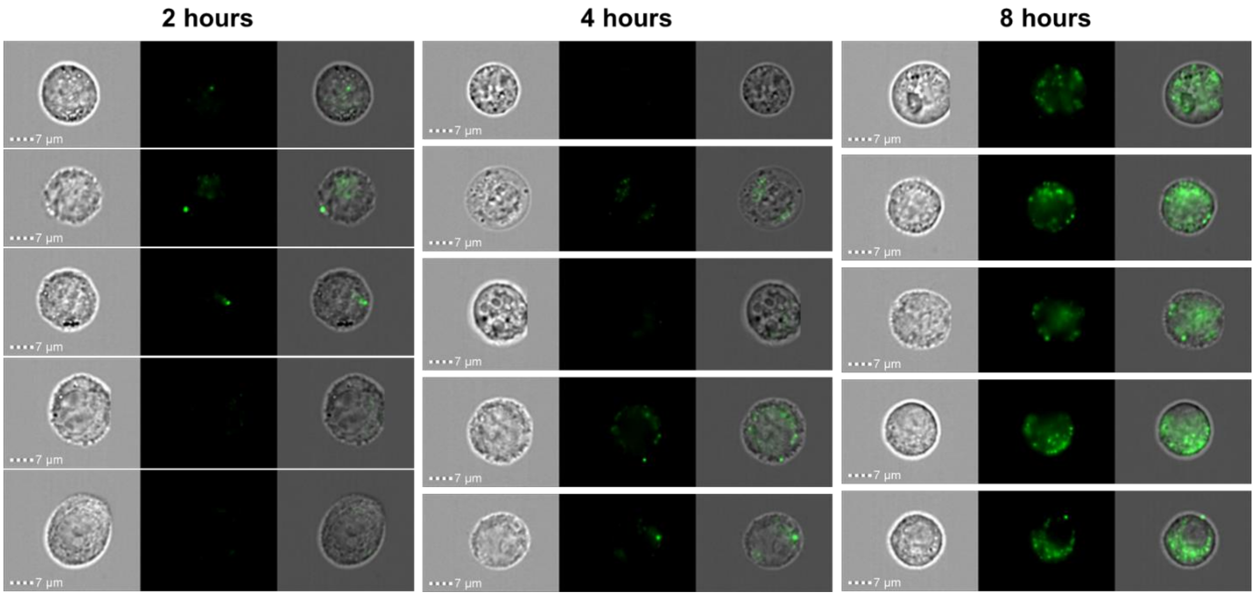

Fig. S39. Imaging flow cytometry images for EV tracking experiments. Additional imaging flow cytometry images of A549 cells stained by the **COE-Ben**-stained small EVs for different treatment times. The fluorescent **COE-Ben** channel was excited using 405 nm, and the emission was collected in the range of 505−560 nm.

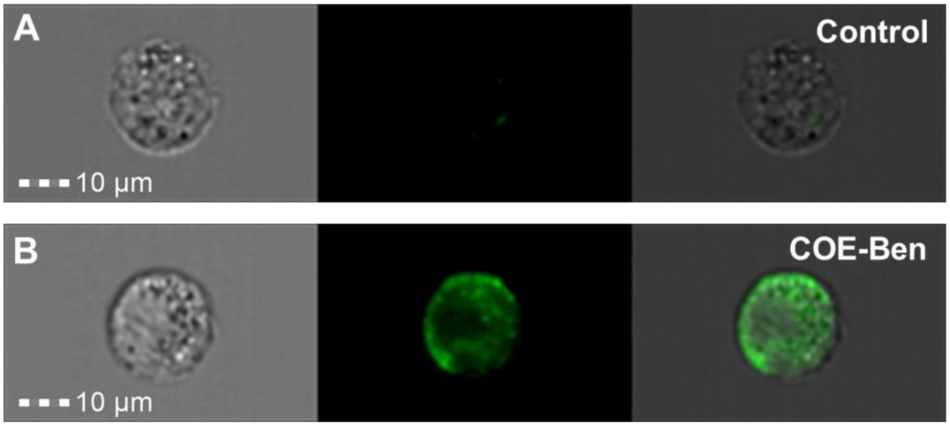

Fig. S40. Imaging flow cytometry measurements. Representative imaging flow cytometry images of A549 cells (**A**) without or (**B**) with directly stained by 2.5 μM **COE-Ben** for 30 minutes.

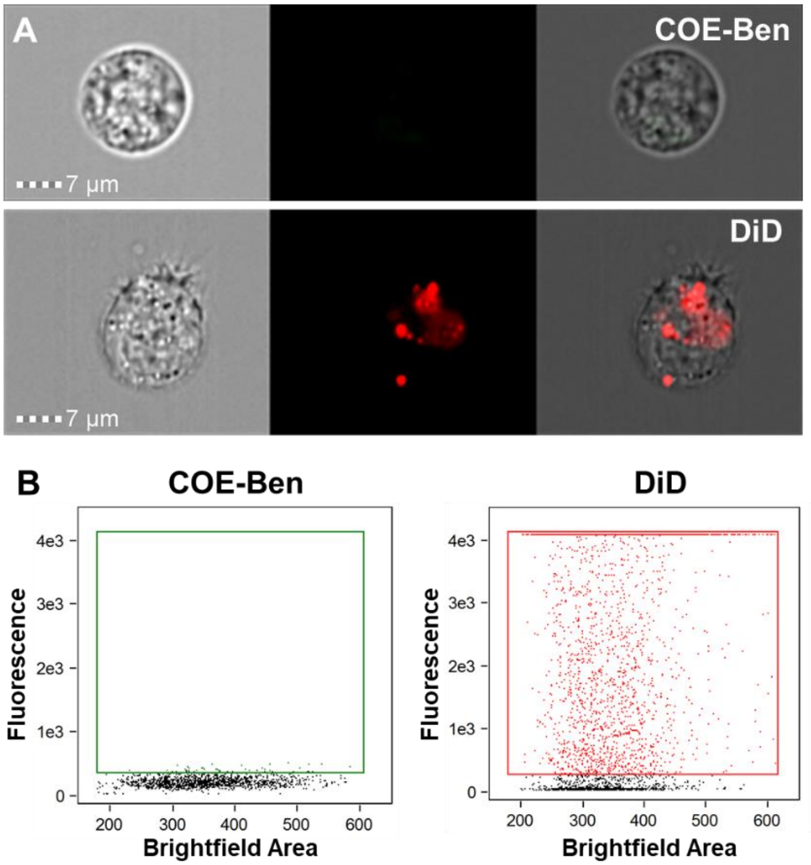

Fig. S41. Background signal analysis for small EV tracking experiments. (**A**) Representative imaging flow cytometry images of A549 cells after being stained by the residual **COE-Ben** or **DiD** dye after washing using ultrafiltration, and (**B**) their corresponding flow cytometric analysis. In the imaging flow cytometry, the **COE-Ben** channel was excited using 405 nm and the emission was collected in the range of 505−560 nm, the **DiD** channel was excited using 638 nm and the emission was collected in the range of 642−745 nm, the brightfield area of the whole cell was employed as the indication of size.

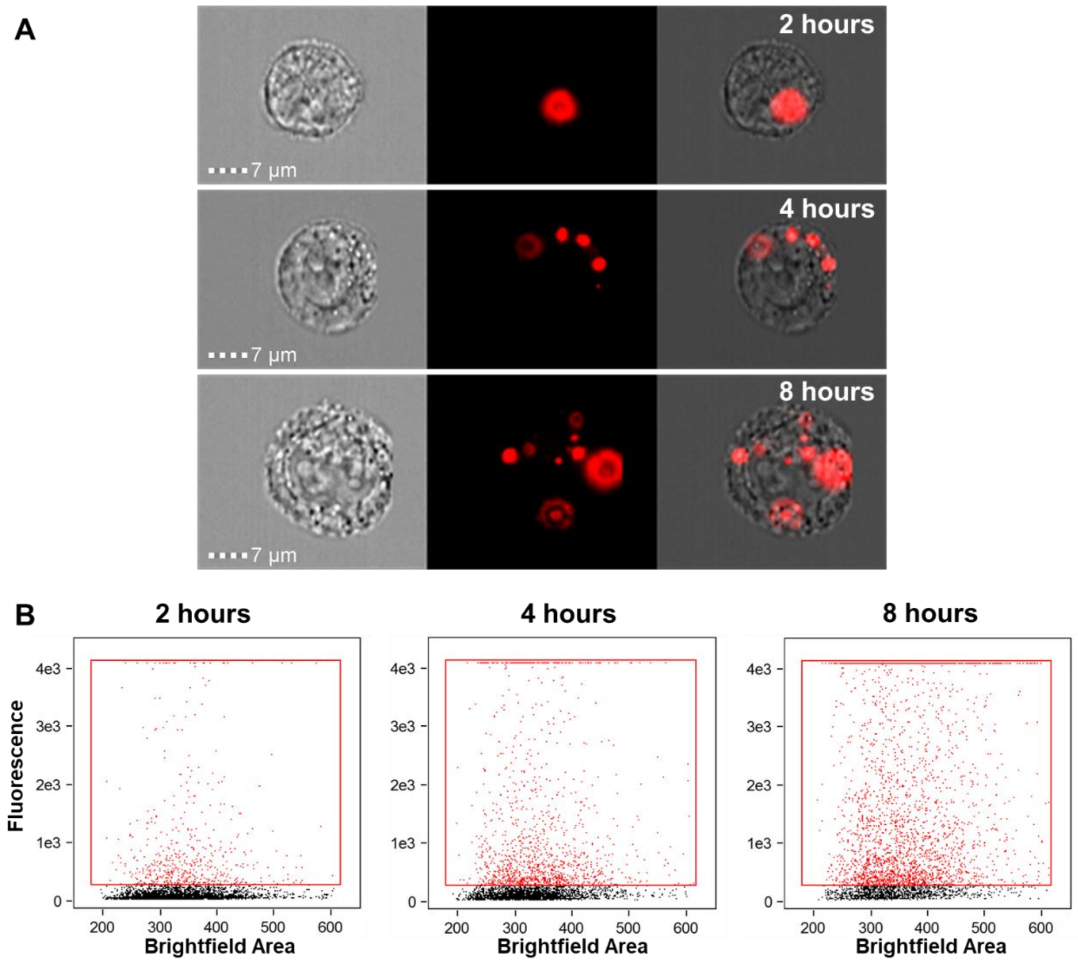

Fig. S42. Tracking of DiD-stained small EVs uptake into mammalian cells. (**A**) Representative imaging flow cytometry images of A549 cells stained by the **DiD**-stained exosomes for different times, and (**B**) their corresponding flow cytometric analysis. The **DiD** channel was excited using 638 nm laser and the emission was collected in the range of 642-745 nm. The brightfield area of the whole cell was employed as the indication of size.

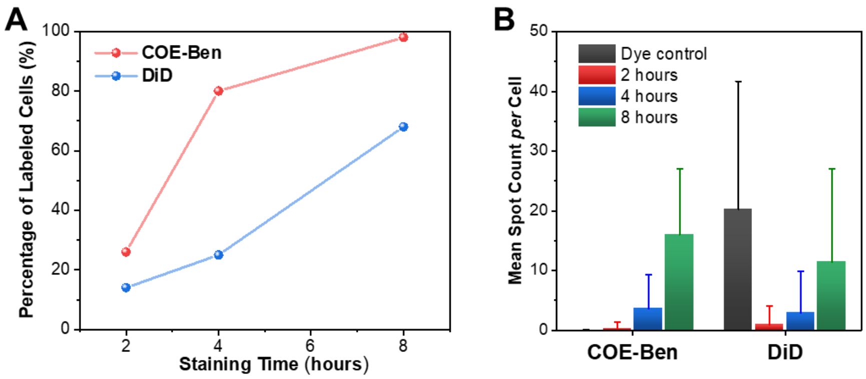

Fig. S43. Comparison between COE-Ben and DiD in the small EV tracking experiments. (**A**) Percentage of cells that were stained by the **COE-Ben-** or **DiD**-stained small EVs at different times. The data were obtained from flow cytometric analysis. (**B**) Mean spot count of **COE-Ben** or **DiD** probe in each cell.

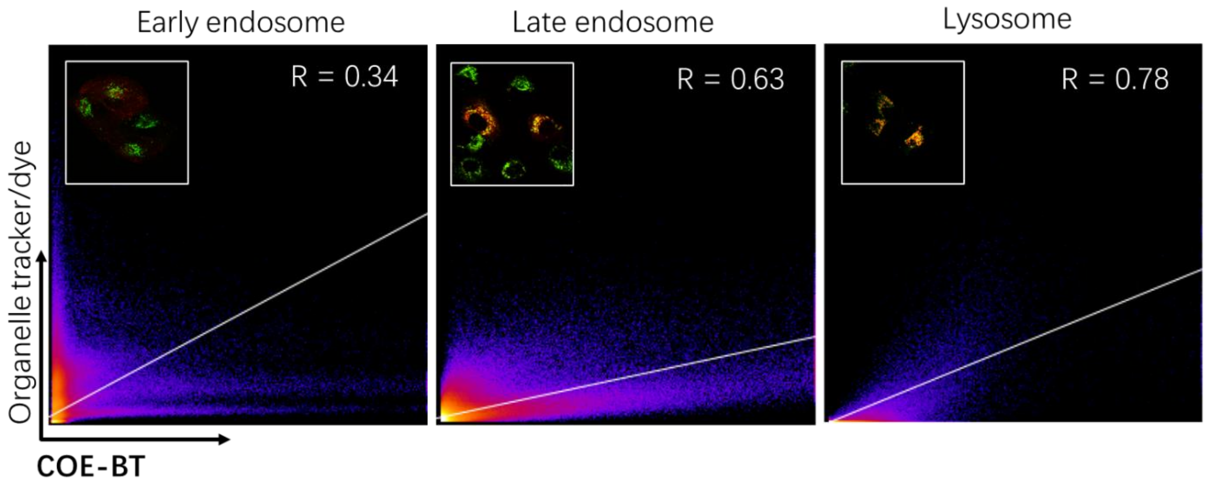

Fig. S44. Confocal microscopy colocalization analysis. Colocalization analysis between **COE-BT** with early or late endosome-specific GFP (green fluorescent protein) or lysosome-specific LysoTracker Green by calculation the Pearson's correlation coefficient (R) using ImageJ.

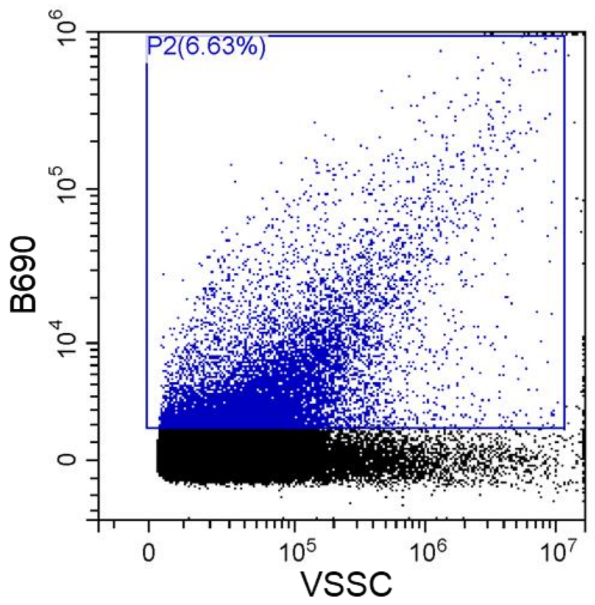

Fig. S45. Direct production of COE-labeled small EVs. Flow cytometry analysis of EVs secreted by **COE-BT**-stained A549 cells after the second 24-hour incubation. The excitation and emission wavelengths are 488/690 nm for the B690 channel. The size was detected by the violet side scatter (VSSC) channel.

Supplementary Measurements

2.1 Agents & Instruments

The lipids 1-palmitoyl-2-oleoyl-glycero-3-phosphocholine (POPC) and 1-palmitoyl-2-oleoyl-sn-glycero-3-phospho-(1'-rac-glycerol) sodium (POPG) were purchased from Avanti Polar Lipids. The liposomes were prepared using an Avanti Mini Extruder. PBS (phosphate-buffered saline, pH = 7.0 − 7.2), DMEM (Dulbecco's modified Eagle medium) and FBS (fetal bovine serum) were purchased Cytiva HyClone™. Bovine serum albumin was purchased from Sigma-Aldrich. Lyophilized PC-3 small EVs derived from cell culture were purchased from Abcam (ab239689). A fresh vial of 100 µg was reconstituted in 1 mL of MiliQ water. The small EV samples were diluted to 10 µg mL^{-1} using PBS, aliquoted into 100 µL in separate Eppendorf tubes, and stored at –80 °C before use.

UV–Vis-NIR absorption spectra were recorded on a Shimadzu UV-3600i plus spectrophotometer. Photoluminescence (PL) spectra were measured on a Horiba Fluorolog-3 fluorescence spectrometer equipped with an R13456 PMT detector for UV-Visible emission and an InGaAs detector (DSS-IGA020L, LN2 cooled) for NIR emission. The micrographs were obtained using the Olympus FV3000 confocal laser scanning microscope or the Leica THUNDER imager fluorescence microscope. Dynamic light scattering (DLS) measurements were performed using the Zetasizer Ultra (Malvern Panalytical). Flow cytometry data were collected using CytoFLEX LX (Beckman Coulter) and NanoAnalyzer (NanoFCM) instruments. Imaging flow cytometry data were collected using the Amnis $ImageStream^{X}$ Mk II Imaging Flow Cytometer.

2.2 Liposome preparation

POPC and POPG in chloroform solution were mixed in a glass test tube to a molar ratio of 85:15 and dried under a gentle stream of argon. The dried lipid was further desiccated in a vacuum overnight to obtain a thin lipid film. To prepare the small unilamellar vesicles (SUVs), rehydration of the dried film was carried out by adding PBS buffer (phosphate-buffered saline) to a concentration of 5 mg mL^{-1}, followed by incubation at 45 °C for 2 hours under constant stirring at ≈300 rpm. Then, the vesicles were extruded using a 100 nm membrane at 45 °C for 21 times to obtain SUV samples. For the SUVs for flow cytometry measurements, the vesicles were extruded using a 200 nm membrane.

To prepare the larger multilamellar vesicles (LMVs) for the confocal microscopy measurements, rehydration of the dried film was carried out by adding PBS to a concentration of 12.5 mg mL^{-1}, followed by incubation at 45 °C for 2 hours under constant stirring at ≈300 rpm. To reduce the experimental error, the 12.5 mg mL^{-1} SUVs solution and dye solution (30 µM **COE-Ben** and 30 µM **FM 4-64**) in PBS were mixed by half-and-half dilution. The stained LMVs were diluted 5 times using PBS before confocal imaging (Olympus FV3000).
All the liposomes were stored at 4 °C before staining and use.

2.3 Cytotoxicity assay

A549 cells were routinely maintained in DMEM supplemented with 10% FBS, i.e. the cell culture medium (CCM). Stock solutions of COE were prepared by dissolving the solids in PBS to achieve 2 mM stock concentrations. The working COE solution at 256 µM was further prepared by diluting the stock solution in CCM. To test for cytotoxicity, 1000 cells were seeded into 96-well black plates with clear optical bottom, and left overnight in the 5% CO_2 chamber at 37 °C to adhere. The next day, different concentrations of COE solutions were achieved by two-fold serial dilution in CCM (concentrations ranged from 0.5 – 256 µM), and then used to replace the spent

CCM in the wells. The cells were then left to incubate with the additives for 24 hours before cell viability was measured either using the CellTiter-Glo® (**COE-B7**, **COE-S6**, **COE-Ben**, **COE-BBT**, **DiR**) or CCK-8 (**COE-BT**, **COE-BO**, **COE-QX**, **COE-BSe**) according to the manufacturer's protocol. Luminscence (CellTiter-Glo® assay) and absorbance (CCK-8 assay) measurements were recorded using the TECAN plate reader (Spark®). The cytotoxicity assay was done in triplicates.

2.4 Hemolysis assay

Bovine whole blood was purchased from Innovative Research, USA. 2 mL blood was mixed with 10 mL PBS and centrifuged at 1,000 rpm for 5 min. Red blood cell pellets were collected and subsequently washed with PBS three times, and then diluted using PBS to a concentration of 2% (v/v). Only **COE-B7**, **COE-S6** and **COE-Ben** were selected in this study, due to they have insignificant absorption interference at 540 nm. Each COE was dissolved in PBS and two folds serial diluted in a 96-well microplate. 100 µL red blood cell suspension was mixed with 100 µL COE solution in each well and incubated for 1 hour at 37 °C under shaking (200 rpm). The microplate was centrifuged at 1,000 rpm for 10 min. 150 µL aliquots of the supernatant were transferred to a new 96-well microplate. Hemolytic activity was calculated by measuring absorbance at 540 nm using the Multimode Microplate Reader (Spark®, Tecan). Triton X-100 (0.1% in PBS) which is able to lyse red blood cells completely was used as a positive control, while bovine erythrocytes in PBS was used as a negative control. The hemolysis percentage was calculated using the following formula:

$$Hemolysis(\%) = \frac{O_c - O_b}{O_t - O_b} \times 100\%$$

where O_c is the absorbance of COE-treated sample, O_b is the absorbance of negative control and O_t is the absorbance of positive control. The hemolysis assay was done in four replicates.

2.5 Flow cytometry analysis on COE-labelled liposomes

Liposomes, comprising 15% POPG and 85% POPC, were prepared as 5 mg/mL (or 6.564 mM) stock in PBS as described before. The liposomes were extruded through 200 nm membrane filters and further characterized by DLS to be approximately 140 nm in size. Separate aliquots of the stock liposomes were then diluted to 1 mM and treated with 20 µM **COE-Ben** and 40 µM **COE-BT** in PBS respectively, under gentle heating to 60°C for 30 min. The labelled liposomes were then stored at 4 °C before being used for flow cytometry experiments.

To demonstrate the stability of the dyes in the liposomes after intercalation, two separate working solutions of **COE-Ben** labelled liposomes and **COE-BT** liposomes were first normalized to the same particle concentration of 10^7 particles per mL. These two working solutions were then mixed in varying ratios of 0:5, 1:4, 2:3, 3:2, 4:1 and 5:0 and the mixtures were allowed to incubate at room temperature for 1 hour. The undiluted mixtures were then analyzed on the CytoFLEX LX (Beckman Coulter) where detection is triggered on the violet side scatter (VSSC >4000). With reference to unstained liposomes, gating was done on both the V525 channel (Ex405/Em525) and B690 channel (Ex488/Em690) for **COE-Ben** labelled liposomes and **COE-BT** labelled liposomes respectively. The percentage of events in the dual-positive region of the dot plot was used to analyze for liposomes that may contain both **COE-Ben** and **COE-BT** dyes, suggesting the transfer of dyes from one population to another. Further, the samples were left to incubate at room temperature overnight (24 hour) before doing the same flow cytometry analysis to check for the

stability of the dyes and the extent of cross-over spillage of the dyes. The experiment was carried out in duplicates.

2.6 Colocalization studies for COE and organelle trackers

A549 cells were seeded in the 8-well chamber slides (ibidi, µ-slide) at a cell density of 5×10^4 cells mL^{-1} (final volume: 200 µL). The cells were left to incubate in the 5% CO_2 chamber at 37°C for 1 hour to adhere before **COE-BT** was introduced to the cell culture medium (CCM) at a final concentration of 2 µM. To track the location of the early and late endosomes, cells were also simultaneously treated with 12 µL of the CellLight™ Early or Late Endosomes-GFP reagent (BacMam 2.0, Invitrogen™) such that the cells were either transfected with the construct to produce Rab5a (for early endosome) or Rab7a (for late endosome) that has been fused with green fluorescent protein (GFP). Cells were then further left to incubate overnight. To track the location of the lysosomes, 100 nM of LysoTracker® Green DND-26 was prepared in fresh media and used to replace the spent CCM. The cells were incubated for 1 hour at 37 °C before imaging. Fluorescent images were obtained via confocal microscopy (FV3000, Olympus); **COE-BT** channel: 561 nm for excitation and 570–620 nm for emission acquisition; GFP/LysoTracker Green channel: 488 nm for excitation and 500–540 nm for emission acquisition. The acquired images were then processed and analyzed on ImageJ and the Coloc2 built-in feature was used to estimate the Pearson’s correlation coefficient that informs the degree of colocalization between the COE channel and the organelle tracker channel.

2.7 Cryo-TEM measurements

Tedpella ultrathin carbon film on Lacey carbon support film and 400-mesh copper grids were glow-discharged in air for 20 seconds. 4 µL of freshly prepared A549 EVs, A549 EVs endogenously labeled with **COE-BT**, 0.5 mg mL^{-1} liposomes (POPC:POPG = 85:15 in molar ratio) or 0.5mg mL^{-1} liposomes stained with 2 uM **COE-BT** added to the grid. Samples were quickly blotted using FEI vitrobot Mark IV. The blotting condition settings are “blot time = 1 s, blot force = 1, wait time = 1 s, temperature = 4 °C, humidity = 100%”. Samples were transferred and then stored in liquid nitrogen. Images were taken using a FEI Tecnai Arctica 200 kV cryo-TEM with a Falcon 3EC Camera at 53,000× Magnification with 1 second exposure time.

2.8 Conventional TEM measurements for small EVs

A 300-mesh copper grid with carbon-coated formvar film was placed on sealing film (Parafilm). A drop (about 30 µL) small EV (0.5 mg mL^{-1}, PC-3 “exosome” standards ab239689 purchased from Abcam) sample in PBS, with or without the treatment of 10 µM **COE-BT**, was pipetted onto the grid. After incubation for 10 minutes, excess liquid was removed from the grid edge by blotting using the tip of filter paper. The grid was moved onto filter paper for 1 minute for further drying. Then, the dried grid was placed on sealing film (Parafilm) again and a drop of 3% uranyl acetate (about 30 µL) was placed for 90 seconds, followed by blotting to remove excess liquid using the tip of filter paper. The grip was moved and dried onto filter paper for 3 hours before observation using the Jeol JEM-1400 plus TEM.

Synthesis Details

The compounds **COE-S6** and **COE-BBT**, and important intermediate (*E*)-trimethyl(5-(4-(3,4,5-tris((6-bromohexyl)oxy)styryl)phenyl)thiophen-2-yl)stannane (compound **1**) were synthesized according to previous literatures.(*5*, *8*) The 4,7-dibromobenzo[c][1,2,5]selenadiazole (compound **2**) and 4,7-dibromobenzo[c][1,2,5]oxadiazole (compound **6**) were purchased from Shenzhen Derthon. Bis(triphenylphosphine)palladium(II) dichloride ($Pd(PPh_3)_2Cl_2$), tetrakis(triphenylphosphine)palladium(0) ($Pd(PPh_3)_4$), 4,7-dibromobenzo[c][1,2,5]thiadiazole (compound **4**), 5,8-dibromoquinoxaline (compound **8**), dry tetrahydrofuran (THF), dry toluene, potassium *tert*-butoxide, diisobutylaluminium hydride solution in THF (1 M), pyridinium chlorochromate, *n*-butyllithium solution in cyclohexane (2 M), tributyl(thiophen-2-yl)stannane (compound **16**), and trimethyltin chloride solution in hexane (1 M) were purchased from Sigma-Aldrich. The reactants ethyl 3,4,5-trihydroxybenzoate (compound **10**), 1-bromo-6-chlorohexane, diethyl (4-bromobenzyl)phosphonate (compound **14**), 1,4-dibromobenzene (compound **19**), 4,4"-dibromo-1,1':4',1"-terphenyl (compound **22**) and trimethylamine solution in methanol (3.2 M) or THF (2 M) were purchased from TCI chemicals. The chloroform, Na_2SO_4, and solvents for the chromatography were purchased from Fisher. These chemicals were used directly without further purification. The ^{1}H NMR and ^{13}C NMR spectra were measured on Bruker AV 400 or AV 500 spectrometers in deuterated chloroform or dimethyl sulfoxide (DMSO). Chemical shifts were reported as δ value (ppm) relative to an internal tetramethylsilane (TMS) standard or the solvent peak.

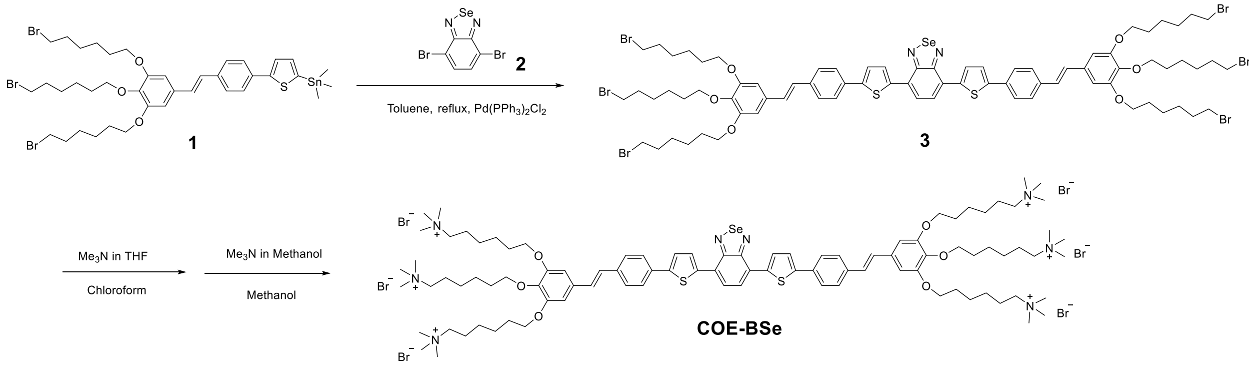

Fig. S46. Synthetic pathway. The synthetical route for **COE-BSe**.

4,7-Bis(5-(4-((*E*)-3,4,5-tris((6-bromohexyl)oxy)styryl)phenyl)thiophen-2-yl)benzo[c][1,2,5]selenadiazole (compound **3**):
(*E*)-trimethyl(5-(4-(3,4,5-tris((6-bromohexyl)oxy)styryl)phenyl)thiophen-2-yl)stannane (compound **1**) (1.29 g, 1.34 mmol), 4,7-dibromobenzo[c][1,2,5]selenadiazole (compound **2**) (0.152 g, 0.446 mmol), and catalyst $Pd(PPh_3)_2Cl_2$ (12.5 mg, 0.0178 mmol) were added to a round flask under the protection of argon atmosphere. After purged using argon, 10 mL dry toluene was added to the reaction mixture, which was then heated at 110 °C for 16 hours with stirring. After

being cooled to room temperature, the reaction mixture was poured into water, and extracted with chloroform. The organic phase was dried over Na_2SO_4 and then the organic solvent was removed by rotary evaporator. The crude product was purified with silica gel column chromatography using hexane : dichloromethane = 1 : 10 as eluent. The product was further dissolved in chloroform and precipitated using methanol, and the precipitation were collected by filtration and washed using methanol. After being dried in vacuum, the product was obtained as dark purple solid (0.548 g, 69 % yield). ^{1}H NMR (500 MHz, Chloroform-*d*, 328K) δ 8.03 (d, *J* = 3.9 Hz, 2H), 7.81 (s, 2H), 7.69 (d, *J* = 8.5 Hz, 4H), 7.52 (d, *J* = 8.4 Hz, 4H), 7.41 (d, *J* = 3.9 Hz, 2H), 7.04 (d, *J* = 16.1 Hz, 2H), 6.98 (d, *J* = 16.2 Hz, 2H), 6.74 (s, 4H), 4.09 – 4.03 (m, 8H), 4.03 – 3.98 (m, 4H), 3.49 – 3.39 (m, 12H), 1.99 – 1.75 (m, 24H), 1.62 – 1.50 (m, 12H). ^{13}C NMR (126 MHz, $CDCl_3$, 328K) δ 158.65, 153.72, 146.11, 139.53, 139.22, 137.38, 133.81, 133.13, 129.47, 129.02, 127.78, 127.75, 127.33, 126.41, 125.93, 124.19, 106.32, 73.68, 69.63, 33.98, 33.85, 33.30, 33.18, 30.58, 29.78, 28.52, 28.36, 25.77.

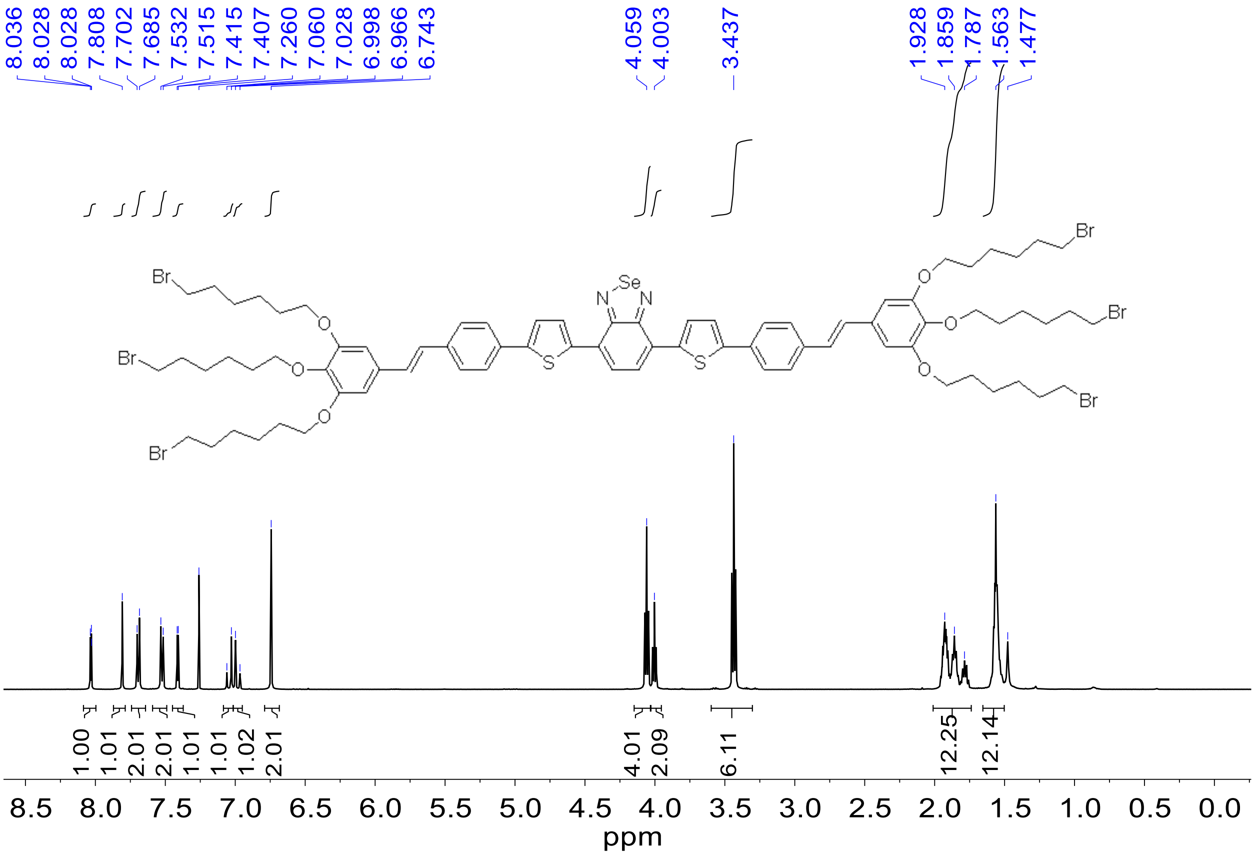

Fig. S47. Structure characterization by NMR. ^{1}H NMR spectrum of compound **3** in deuterated chloroform.

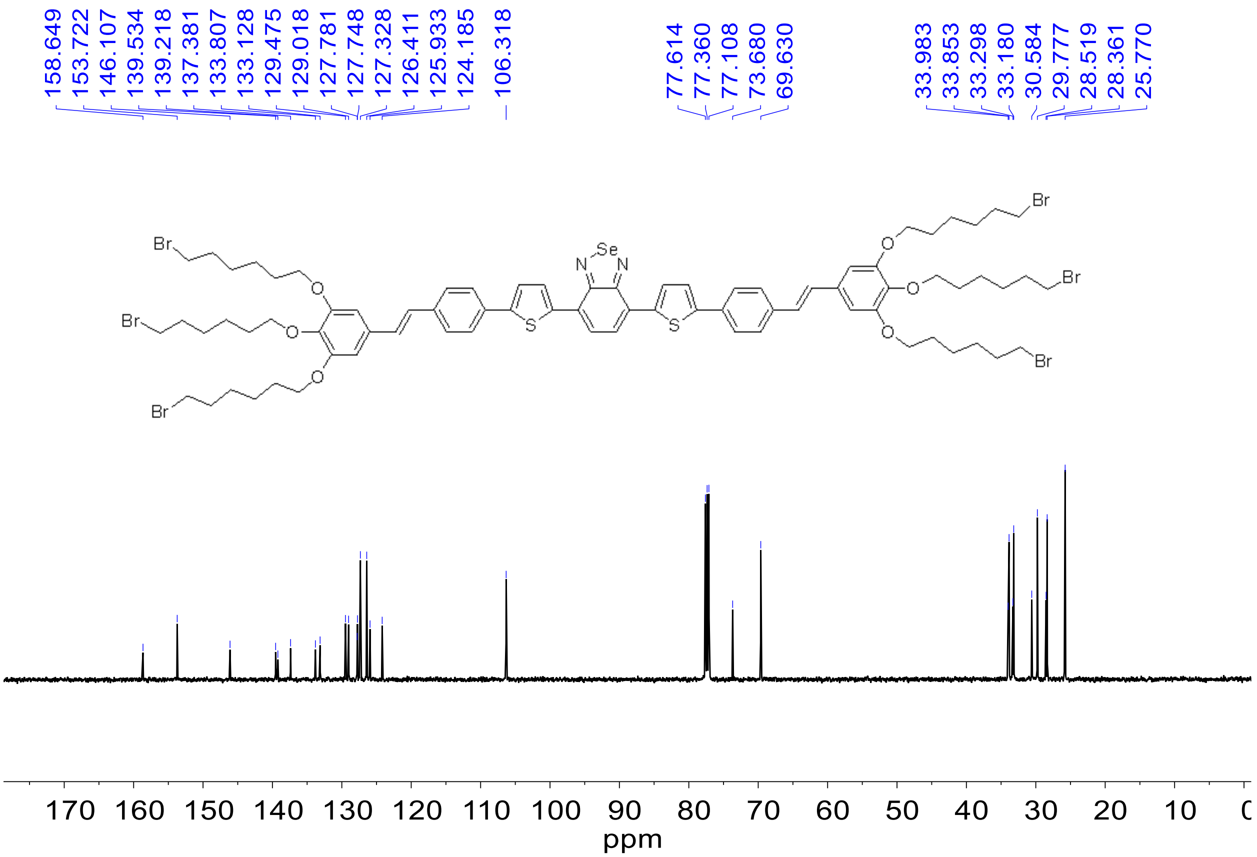

Fig. S48. Structure characterization by NMR. ^{13}C NMR spectrum of compound **3** in deuterated chloroform.

Compound **COE-BSe**:

A single-neck round flask was charged with compound **3** (284 mg, 0.160 mmol) and chloroform (20 mL). After the compound **3** dissolved, 5 mL trimethylamine solution in THF (2 M) was added into the reaction mixture and stirred at 55 °C for 16 h. After the reaction, the crude product was precipitated, which was then filtered and washed using chloroform. Then, the solid precipitation was re-dissolved using 20 mL methanol. 5 mL trimethylamine solution in methanol (3.2 M) was added into the reaction mixture and stirred at 55 °C for another 16 hours. The solvent was removed via rotary evaporation and the solid was dried in vacuum. The final product was obtained as dark purple solid (308 mg, 90 % yield). ^{1}H NMR (500 MHz, DMSO-d_6, 353K) δ 8.15 (d, J = 3.9 Hz, 2H), 8.05 (s, 2H), 7.78 (d, J = 8.1 Hz, 4H), 7.72 – 7.64 (m, 6H), 7.21 (br, 4H), 6.94 (s, 4H), 4.12 – 4.05 (m, 8H), 3.98 – 3.92 (m, 4H), 3.42 – 3.32 (m, 12H), 3.11 (s, 54H), 1.85 – 1.69 (m, 24H), 1.60 – 1.50 (m, 12H), 1.47 – 1.34 (m, 12H). ^{13}C NMR (126 MHz, DMSO, 353K) δ 157.59, 153.29, 145.61, 139.01, 138.56, 137.45, 133.26, 133.05, 129.58, 129.15, 127.65, 127.59, 127.01, 126.13, 125.79, 124.78, 106.54, 73.03, 69.28, 66.23, 53.05, 30.04, 29.26, 26.24, 26.10, 25.58, 25.53, 22.69.

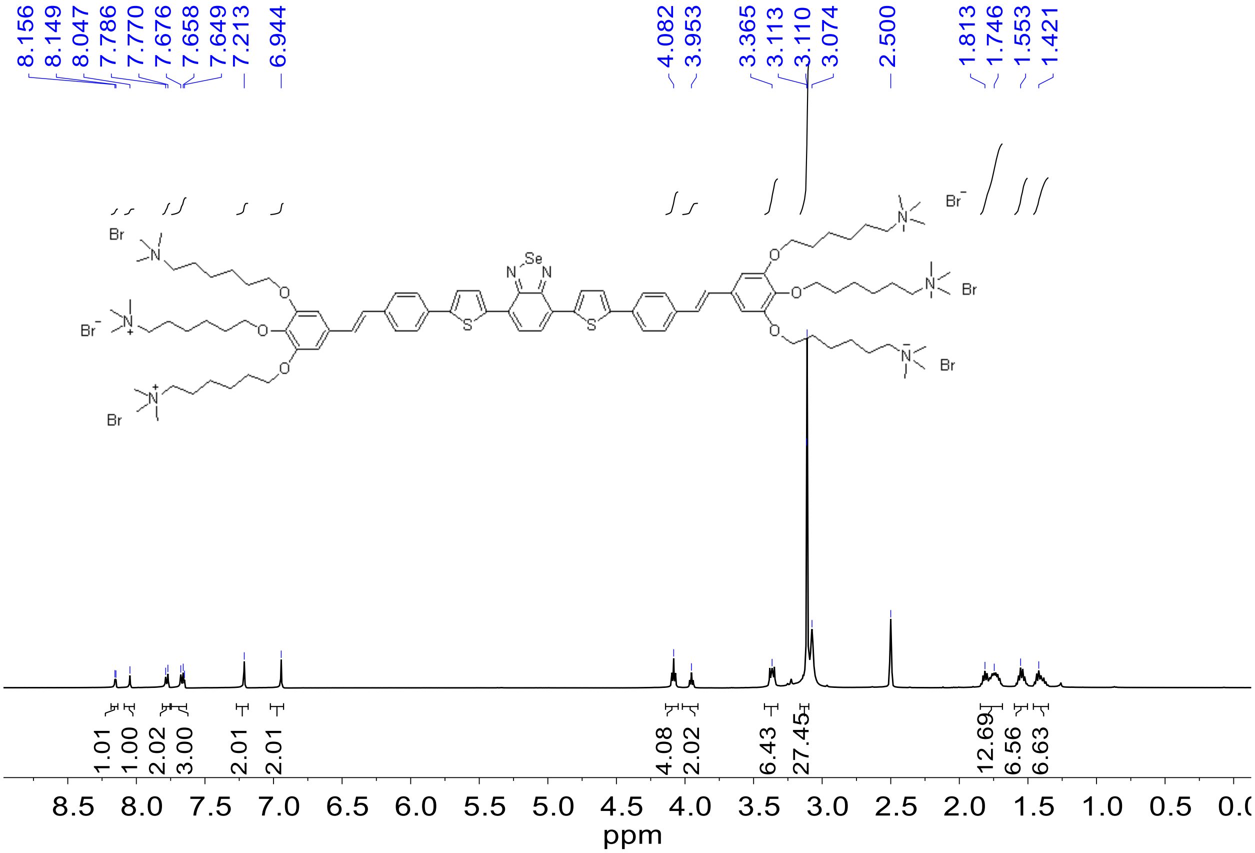

Fig. S49. Structure characterization by NMR. ^{1}H NMR spectrum of compound **COE-BSe** in deuterated DMSO.

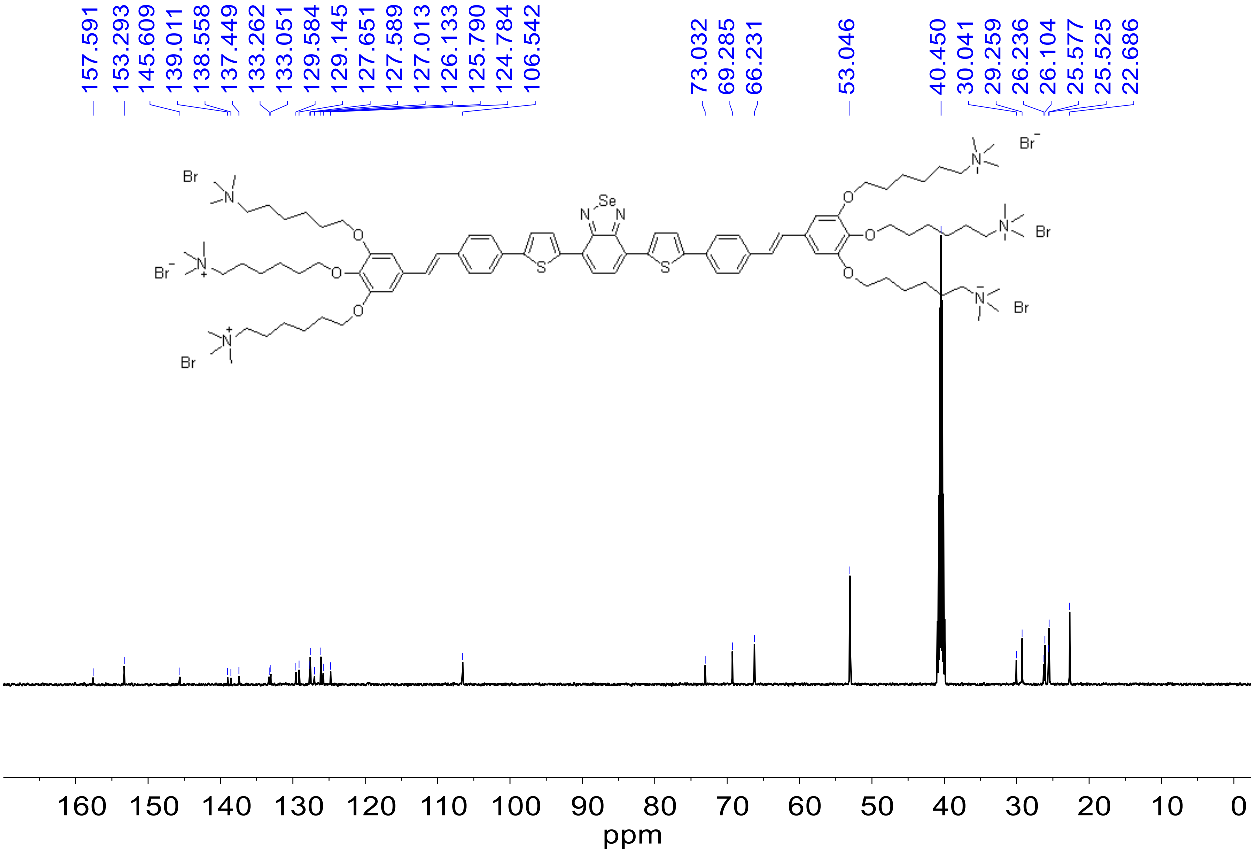

Fig. S50. Structure characterization by NMR. ^{13}C NMR spectrum of compound **COE-BSe** in deuterated DMSO.

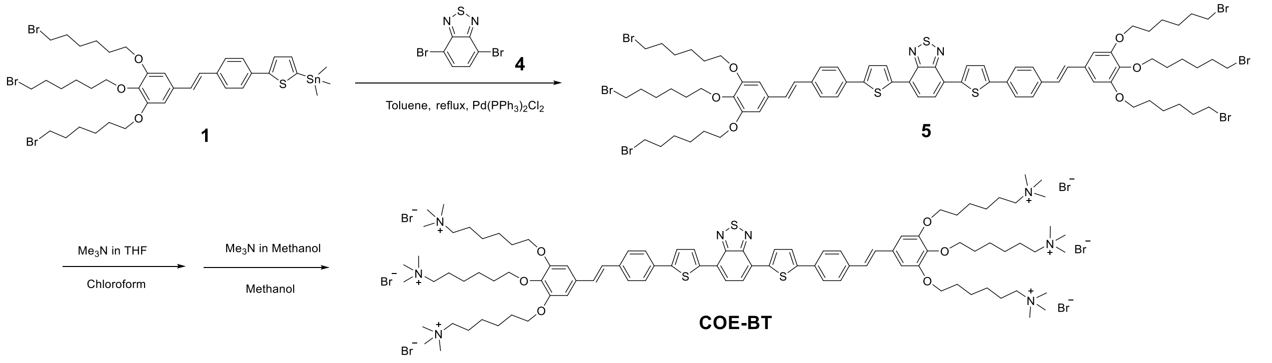

Fig. S51. Synthetic pathway. The synthetical route for **COE-BT**.

4,7-Bis(5-(4-((*E*)-3,4,5-tris((6-bromohexyl)oxy)styryl)phenyl)thiophen-2-yl)benzo[c][1,2,5]thiadiazole (compound **5**):

This compound was synthesized according to a similar procedure as described for compound **3** above, but the reactant 4,7-dibromobenzo[c][1,2,5]selenadiazole (compound **2**) was replaced to 4,7-dibromobenzo[c][1,2,5]thiadiazole (compound **4**). The final product was obtained as a dark purple solid (640 mg, 73 % yield). ^{1}H NMR (500 MHz, Chloroform-*d*, 298K) δ 8.12 (d, *J* = 3.9 Hz, 2H), 7.89 (s, 2H), 7.69 (d, *J* = 8.4 Hz, 4H), 7.53 (d, *J* = 8.5 Hz, 4H), 7.43 (d, *J* = 3.8 Hz, 2H), 7.05 (d, *J* = 16.2 Hz, 2H), 6.99 (d, *J* = 16.2 Hz, 2H), 6.73 (s, 4H), 4.08 – 4.02 (m, 8H), 4.00 – 3.96 (m, 4H), 3.50 – 3.39 (m, 12H), 1.97 – 1.74 (m, 24H), 1.60 – 1.47 (m, 24H). ^{13}C NMR (126 MHz, $CDCl_3$, 298K) δ 153.55, 152.90, 145.65, 138.96, 138.57, 137.22, 133.48, 132.96, 129.31, 129.03, 127.54, 127.26, 126.30, 126.06, 125.64, 124.36, 105.60, 73.60, 69.26, 34.28, 34.15, 33.21, 33.09, 30.50, 29.64, 28.46, 28.29, 25.72, 25.71.

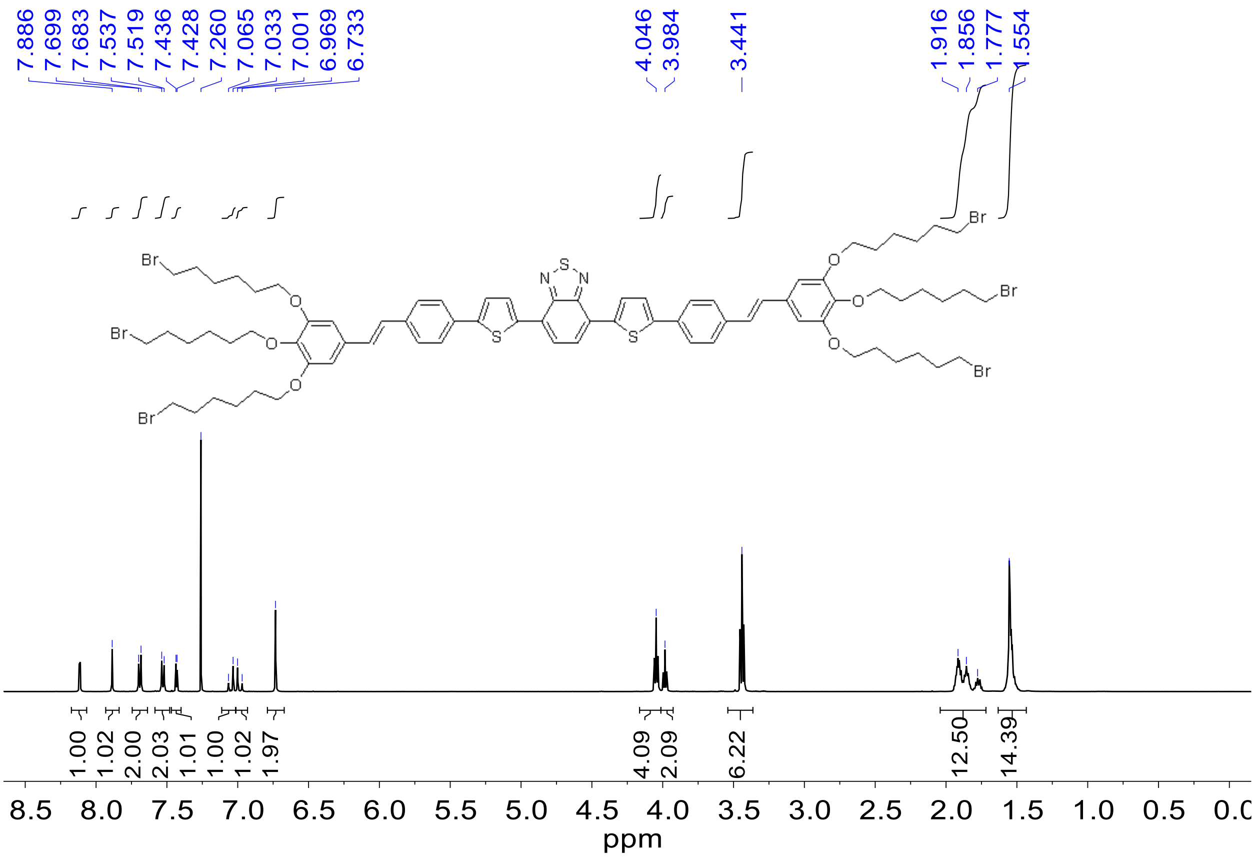

Fig. S52. Structure characterization by NMR. ^{1}H NMR spectrum of compound **5** in deuterated chloroform.

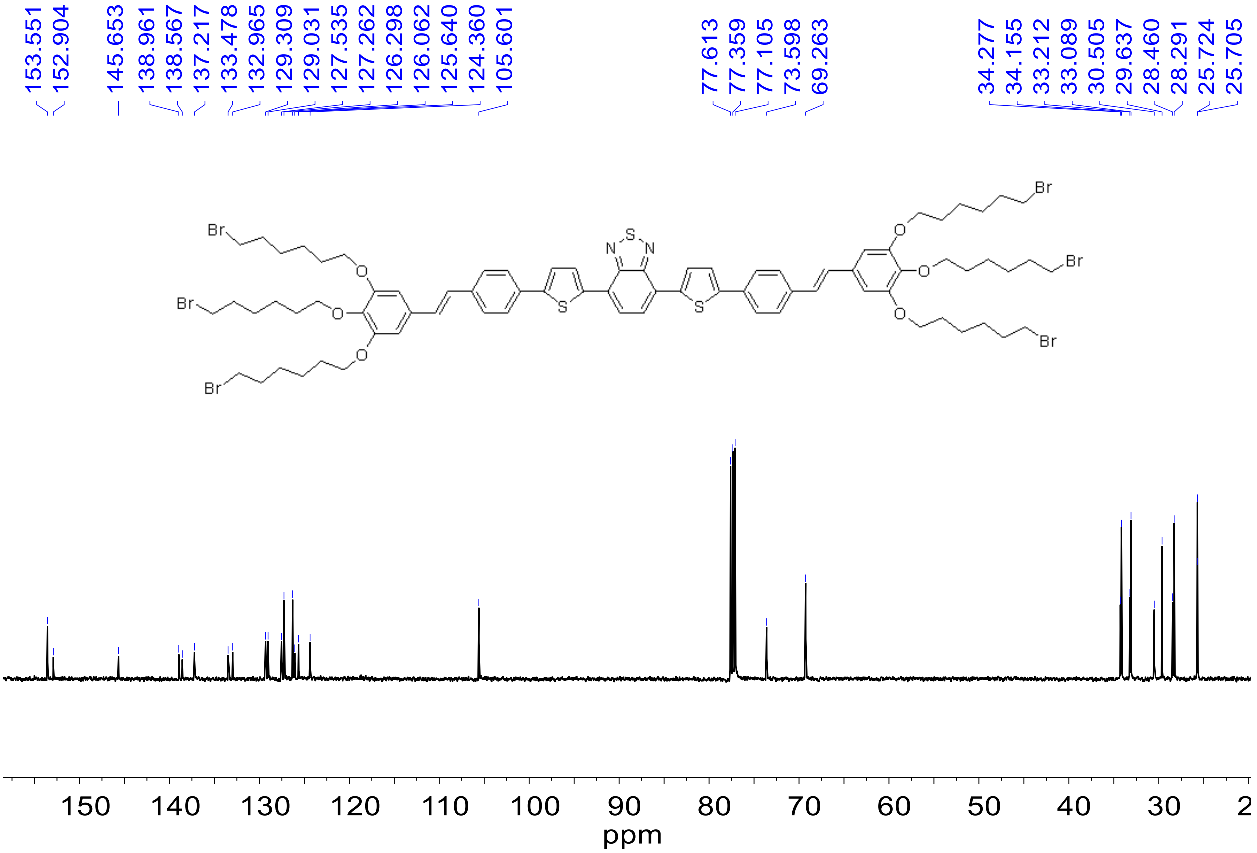

Fig. S53. Structure characterization by NMR. ^{13}C NMR spectrum of compound **5** in deuterated chloroform.

Compound **COE-BT**:

To obtain this compound, compound **5** was used for a similar quaternization reaction as described above for compound **COE-BSe**. The product **COE-BT** was afforded as a dark purple solid (221 mg, 89 % yield). ^{1}H NMR (500 MHz, DMSO-d_6, 298K) δ 8.29 – 8.19 (m, 4H), 7.81 (d, J = 8.0 Hz, 4H), 7.75 (d, J = 3.9 Hz, 2H), 7.68 (d, J = 8.2 Hz, 4H), 7.27 (br, 4H), 6.96 (s, 4H), 4.09 – 4.01 (m, 8H), 3.95 – 3.85 (m, 4H), 3.34 – 3.27 (m, 12H), 3.09 (s, 54H), 1.84 – 1.62 (m, 24H), 1.58 – 1.45 (m, 12H), 1.42 – 1.27 (m, 12H). ^{13}C NMR (126 MHz, DMSO, 298K) δ 153.57, 152.56, 145.89, 138.53, 138.02, 137.88, 133.42, 133.28, 129.93, 129.76, 128.01, 127.88, 126.56, 126.49, 125.68, 125.60, 106.00, 73.27, 69.11, 66.13, 60.82, 53.09, 30.40, 29.56, 26.54, 26.42, 26.02, 25.97, 23.01.

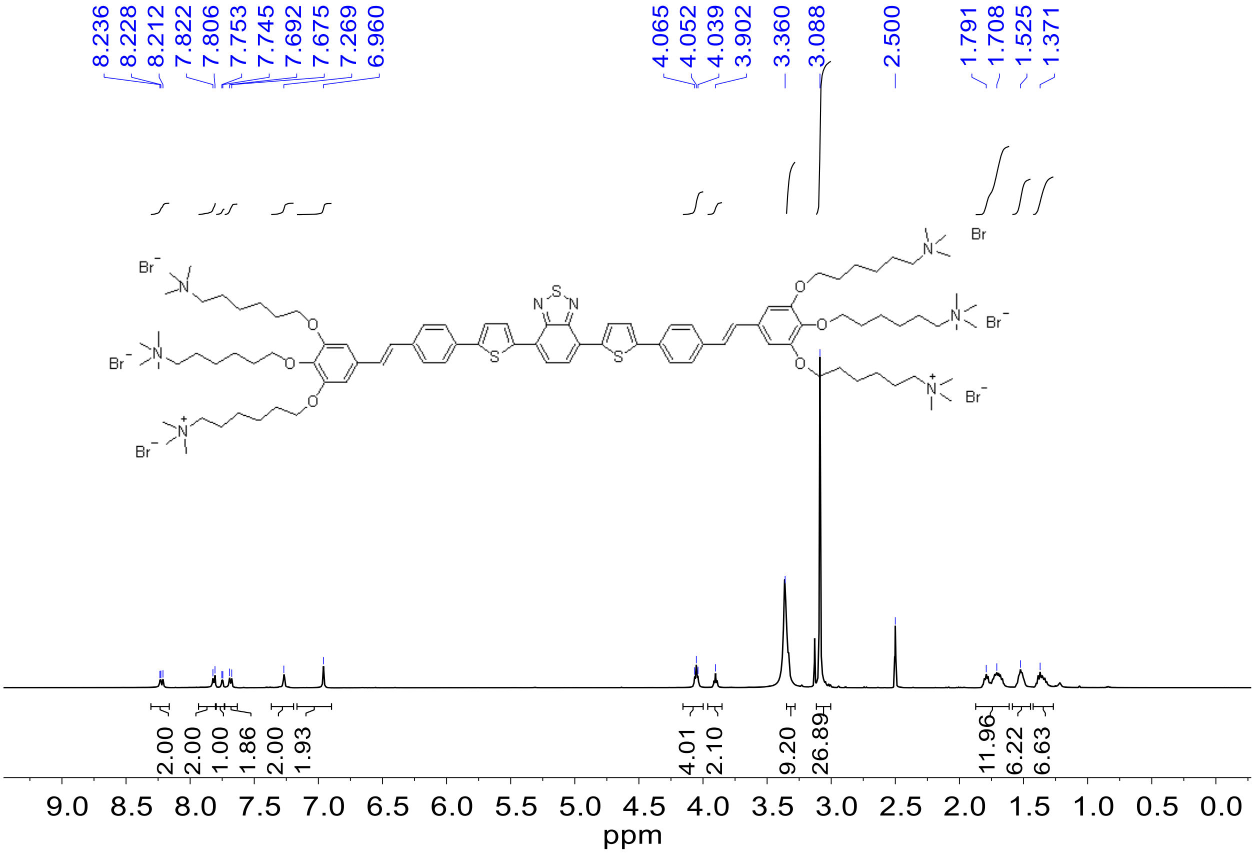

Fig. S54. Structure characterization by NMR. ^{1}H NMR spectrum of compound **COE-BT** in deuterated DMSO.

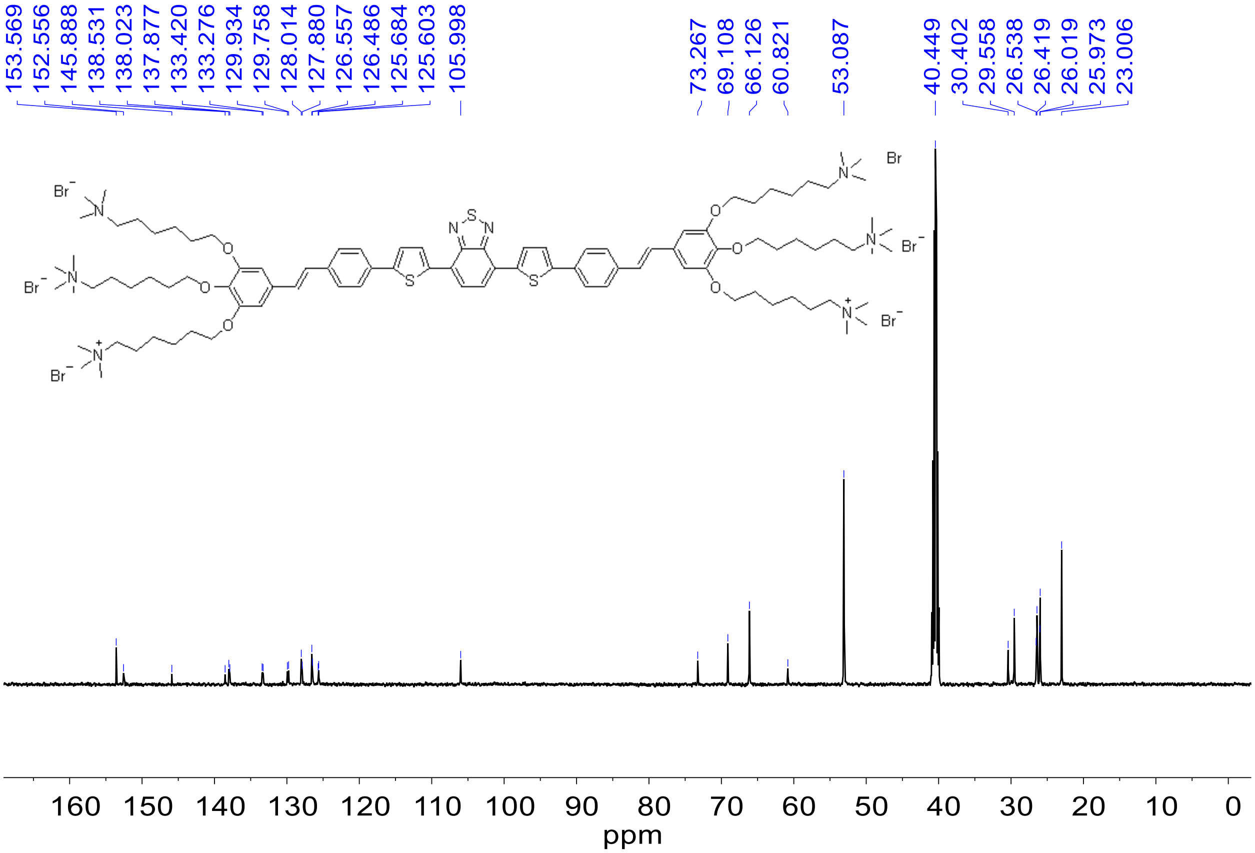

Fig. S55. Structure characterization by NMR. ^{13}C NMR spectrum of compound **COE-BT** in deuterated DMSO.

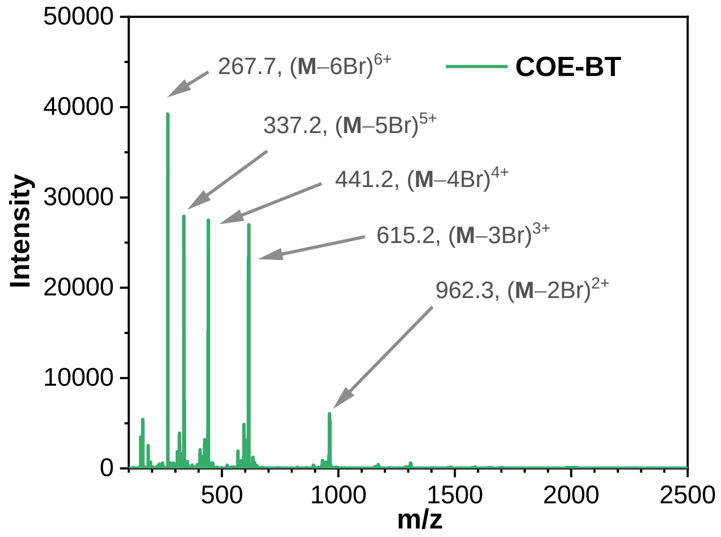

Fig. S56. Structure characterization by MS. Mass spectrum (MS) of **COE-BT** using time-of-flight mass spectrometry with electrospray ionization in the positive-ion mode (TOF MS ES+).

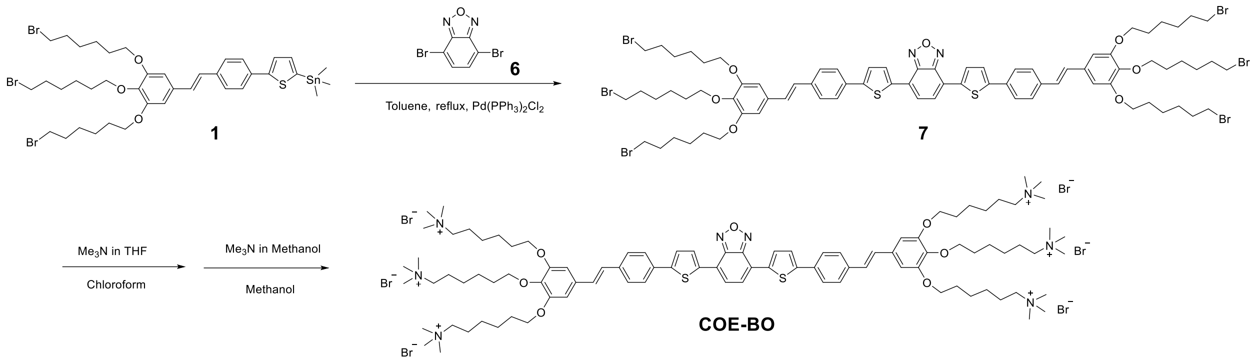

Fig. S57. Synthetic pathway. The synthetical route for **COE-BO**.

4,7-Bis(5-(4-((*E*)-3,4,5-tris((6-bromohexyl)oxy)styryl)phenyl)thiophen-2-yl)benzo[c][1,2,5]oxadiazole (compound **7**):

This compound was synthesized according to a similar procedure as described for compound **3** above, but the reactant 4,7-dibromobenzo[c][1,2,5]selenadiazole (compound **2**) was replaced to 4,7-dibromobenzo[c][1,2,5]oxadiazole (compound **6**). The final product was obtained as a dark purple solid (461 mg, 66 % yield). ^{1}H NMR (500 MHz, Chloroform-*d*, 328K) δ 8.09 (d, *J* = 3.9 Hz, 2H), 7.66 (d, *J* = 8.3 Hz, 4H), 7.60 (s, 2H), 7.52 (d, *J* = 8.2 Hz, 4H), 7.41 (d, *J* = 3.9 Hz, 2H), 7.05 (d, *J* = 16.1 Hz, 2H), 6.97 (d, *J* = 16.2 Hz, 2H), 6.74 (s, 4H), 4.10 – 3.96 (m, 12H), 3.49 – 3.39 (m, 12H), 1.97 – 1.75 (m, 24H), 1.62 – 1.50 (m, 24H). ^{13}C NMR (126 MHz, $CDCl_3$, 328K) δ 153.75, 148.30, 146.19, 139.31, 137.83, 137.57, 133.24, 133.03, 130.37, 129.79, 127.58, 127.39, 126.51, 126.32, 124.90, 122.33, 106.36, 73.69, 69.64, 33.98, 33.85, 33.30, 33.18, 30.59, 29.78, 28.52, 28.36, 25.77.

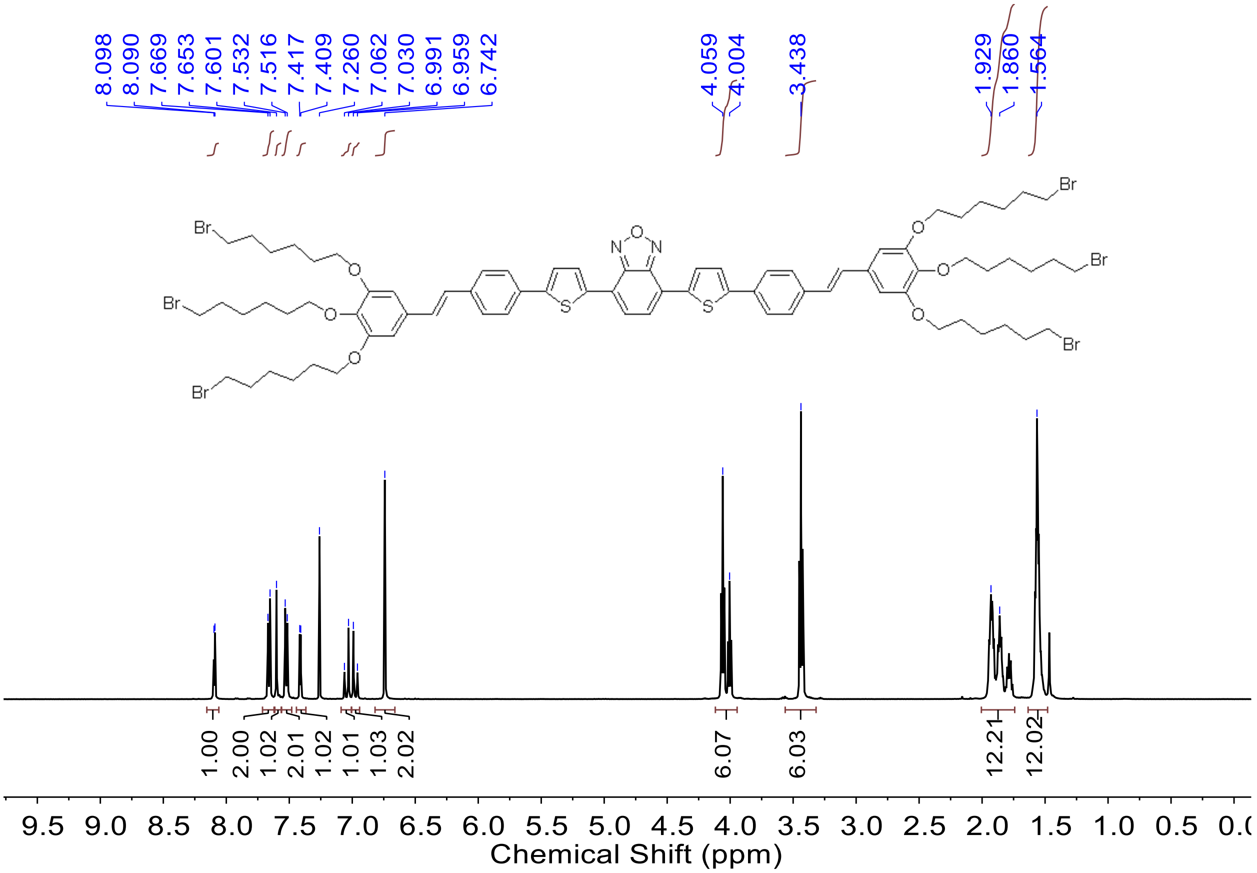

Fig. S58. Structure characterization by NMR. ^{1}H NMR spectrum of compound **7** in deuterated chloroform.

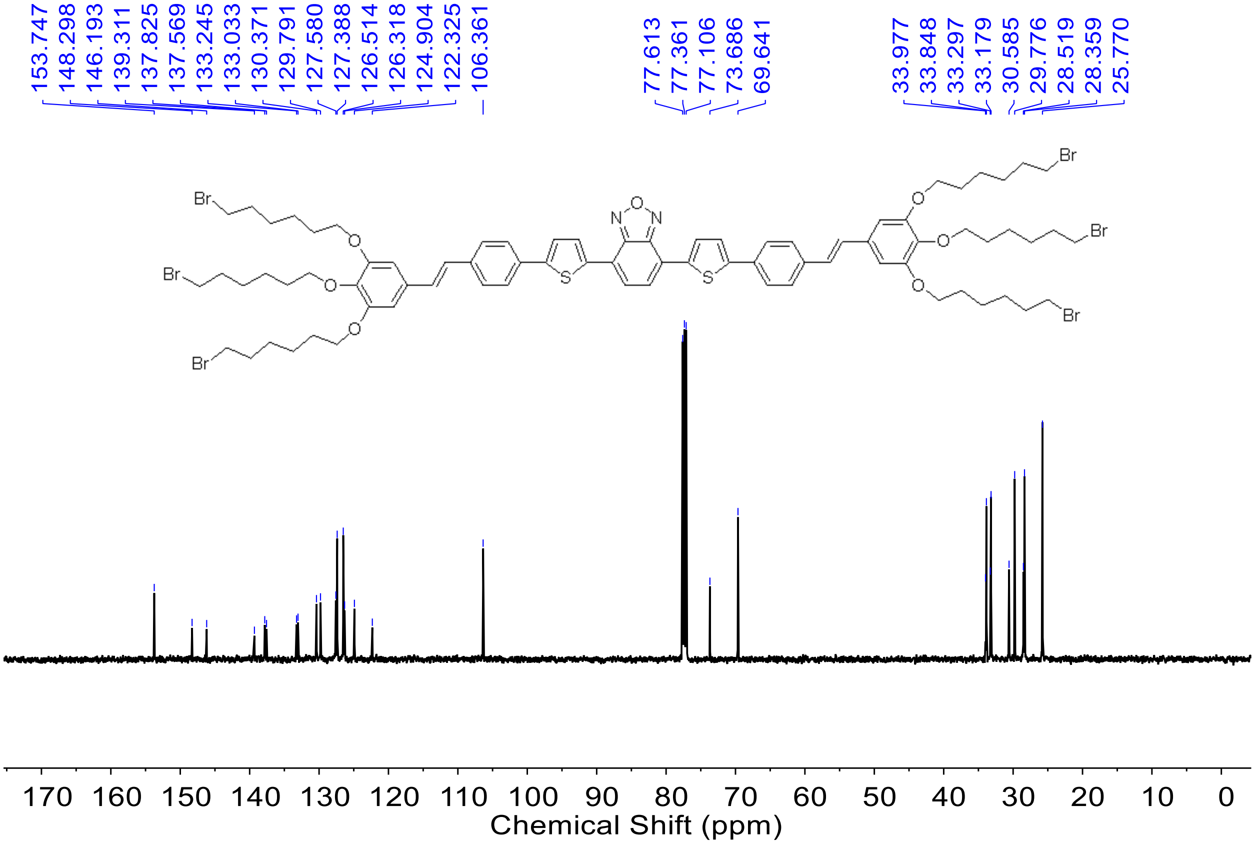

Fig. S59. Structure characterization by NMR. ^{13}C NMR spectrum of compound **7** in deuterated chloroform.

Compound **COE-BO**:

To obtain this compound, compound **7** was used for a similar quaternization reaction as described above for compound **COE-BSe**. The product **COE-BO** was afforded as a dark purple solid (367 mg, 92 % yield). ^{1}H NMR (500 MHz, DMSO-d_6, 353K) δ 8.11 (d, J = 3.9 Hz, 2H), 7.96 (s, 2H), 7.79 (d, J = 8.4 Hz, 4H), 7.71 (d, J = 3.9 Hz, 2H), 7.68 (d, J = 8.6 Hz, 4H), 7.23 (s, 4H), 6.95 (s, 4H), 4.13 – 4.04 (m, 8H), 3.98 – 3.92 (m, 4H), 3.42 – 3.33 (m, 12H), 3.12 (s, 54H), 1.86 – 1.67 (m, 24H), 1.56 (s, 12H), 1.46 – 1.35 (m, 12H). ^{13}C NMR (126 MHz, DMSO, 353K) δ 153.31, 148.11, 146.04, 138.63, 137.97, 136.87, 133.01, 132.65, 130.15, 129.92, 127.80, 127.65, 127.56, 126.39, 125.69, 121.28, 106.61, 79.70, 73.05, 69.30, 66.23, 53.05, 30.04, 29.26, 26.24, 26.10, 25.58, 25.53, 22.69.

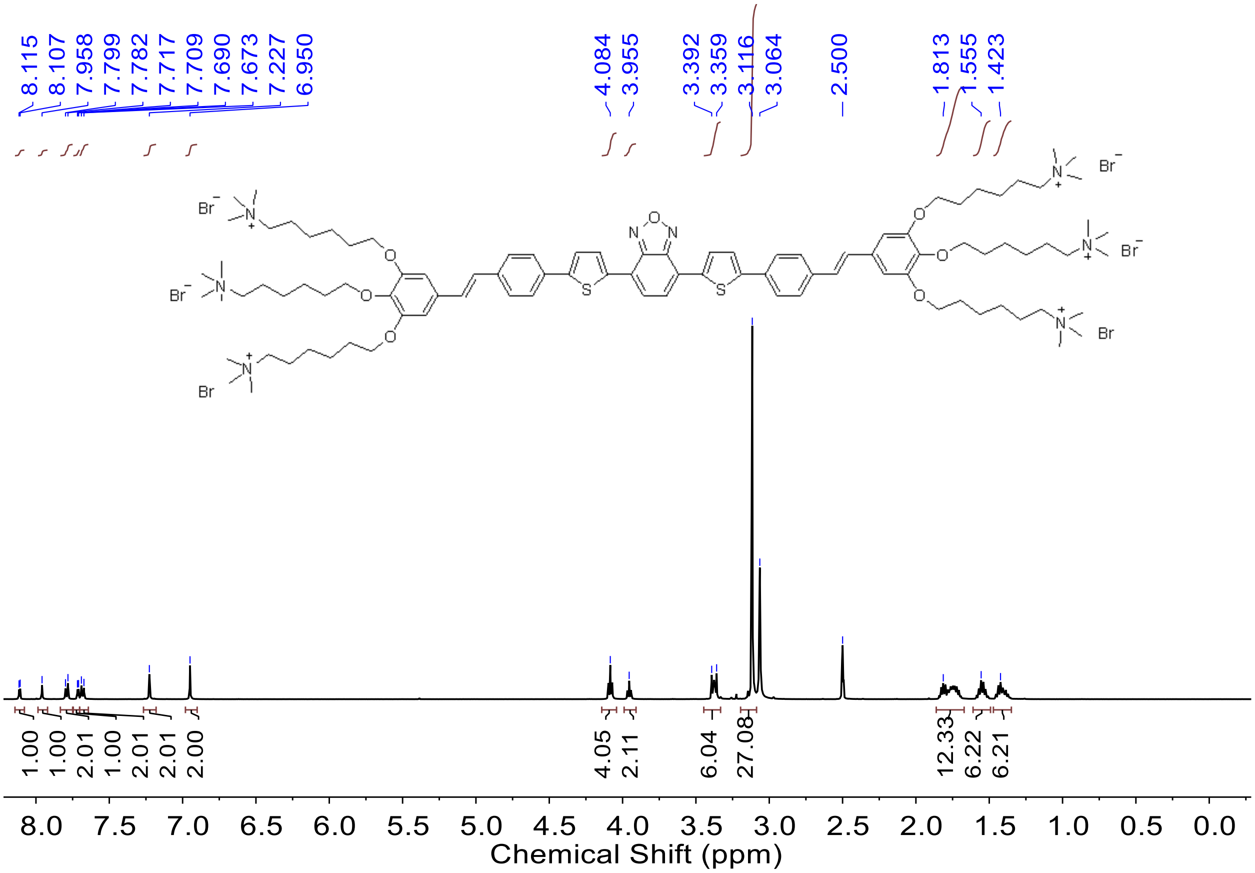

Fig. S60. Structure characterization by NMR. ^{1}H NMR spectrum of compound **COE-BO** in deuterated DMSO.

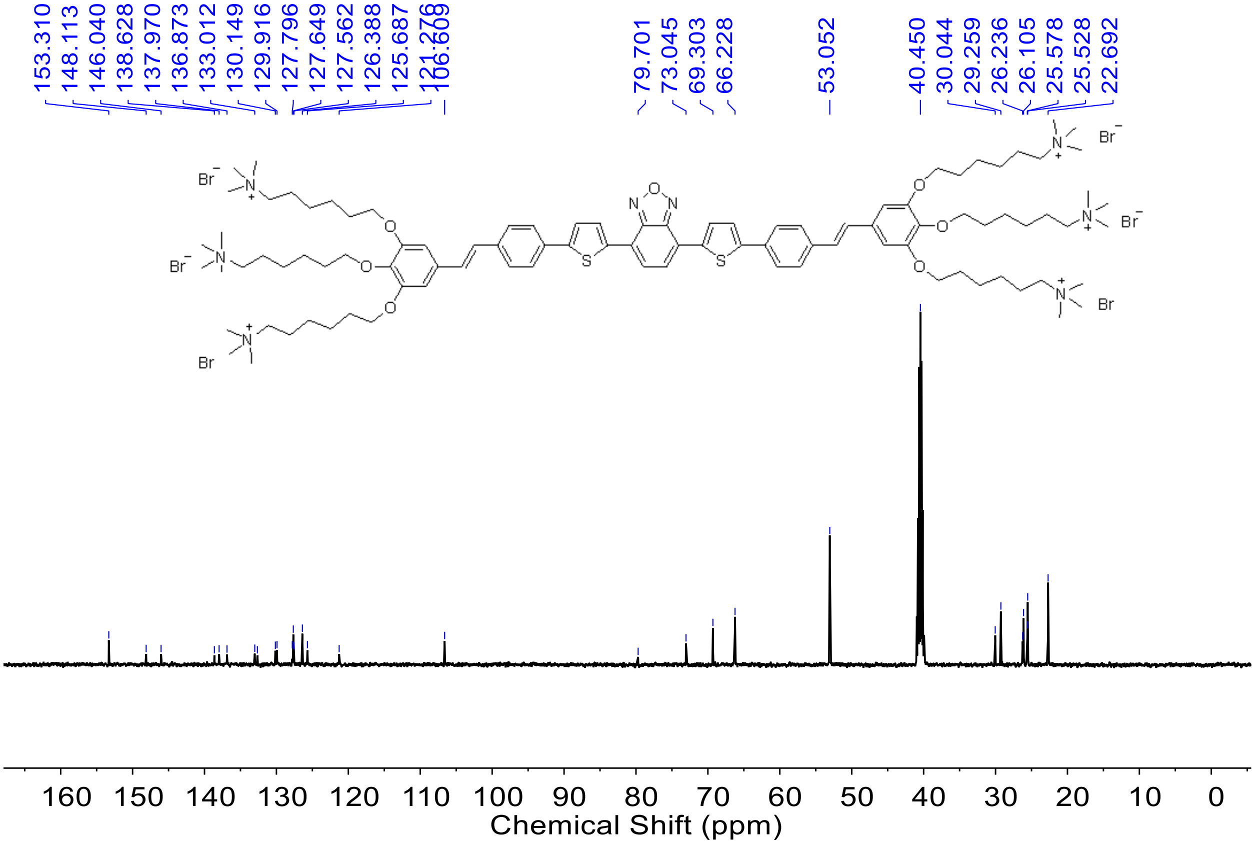

Fig. S61. Structure characterization by NMR. ^{13}C NMR spectrum of compound **COE-BO** in deuterated DMSO.

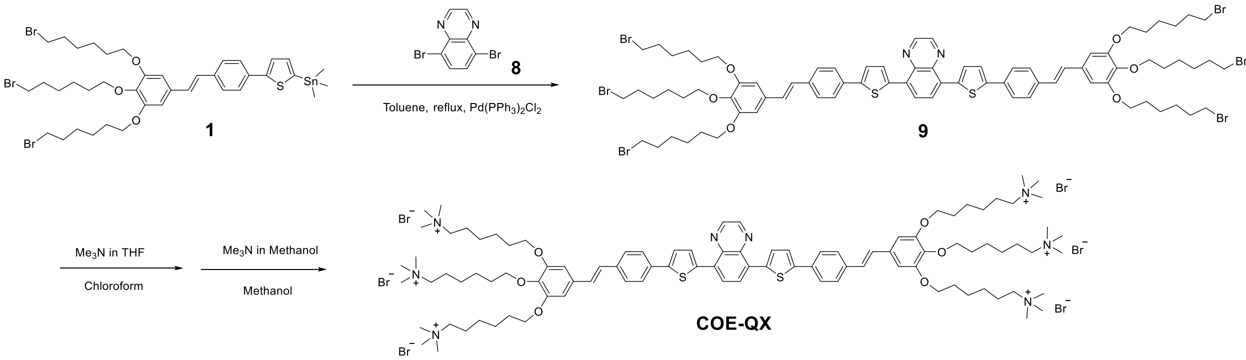

Fig. S62. Synthetic pathway. The synthetical route for **COE-QX**.

5,8-Bis(5-(4-((*E*)-3,4,5-tris((6-bromohexyl)oxy)styryl)phenyl)thiophen-2-yl)quinoxaline (compound **9**):

This compound was synthesized according to a similar procedure as described for compound **3** above, but the reactant 4,7-dibromobenzo[c][1,2,5]selenadiazole (compound **2**) was replaced to 5,8-dibromoquinoxaline (compound **8**). The final product was obtained as a deep red solid (519 mg, 61 % yield). ^{1}H NMR (500 MHz, Chloroform-*d*, 328K) δ 9.01 (s, 2H), 8.16 (s, 2H), 7.85 (d, *J* = 3.9 Hz, 2H), 7.72 (d, *J* = 8.3 Hz, 4H), 7.53 (d, *J* = 8.5 Hz, 4H), 7.42 (d, *J* = 4.0 Hz, 2H), 7.05 (d, *J* = 16.2 Hz, 2H), 6.99 (d, *J* = 16.1 Hz, 2H), 6.75 (s, 4H), 4.12 – 3.96 (m, 12H), 3.51 – 3.36 (m, 12H), 1.99 – 1.74 (m, 24H), 1.62 – 1.50 (m, 24H). ^{13}C NMR (126 MHz, $CDCl_3$, 328K) δ 153.73, 147.31, 143.78, 140.40, 139.20, 138.46, 137.22, 134.15, 133.18, 132.43, 129.35, 128.66, 127.85, 127.68, 127.32, 126.43, 123.50, 106.32, 77.61, 77.36, 77.11, 73.68, 69.63, 33.98, 33.85, 33.30, 33.18, 30.58, 29.77, 28.52, 28.36, 25.77.

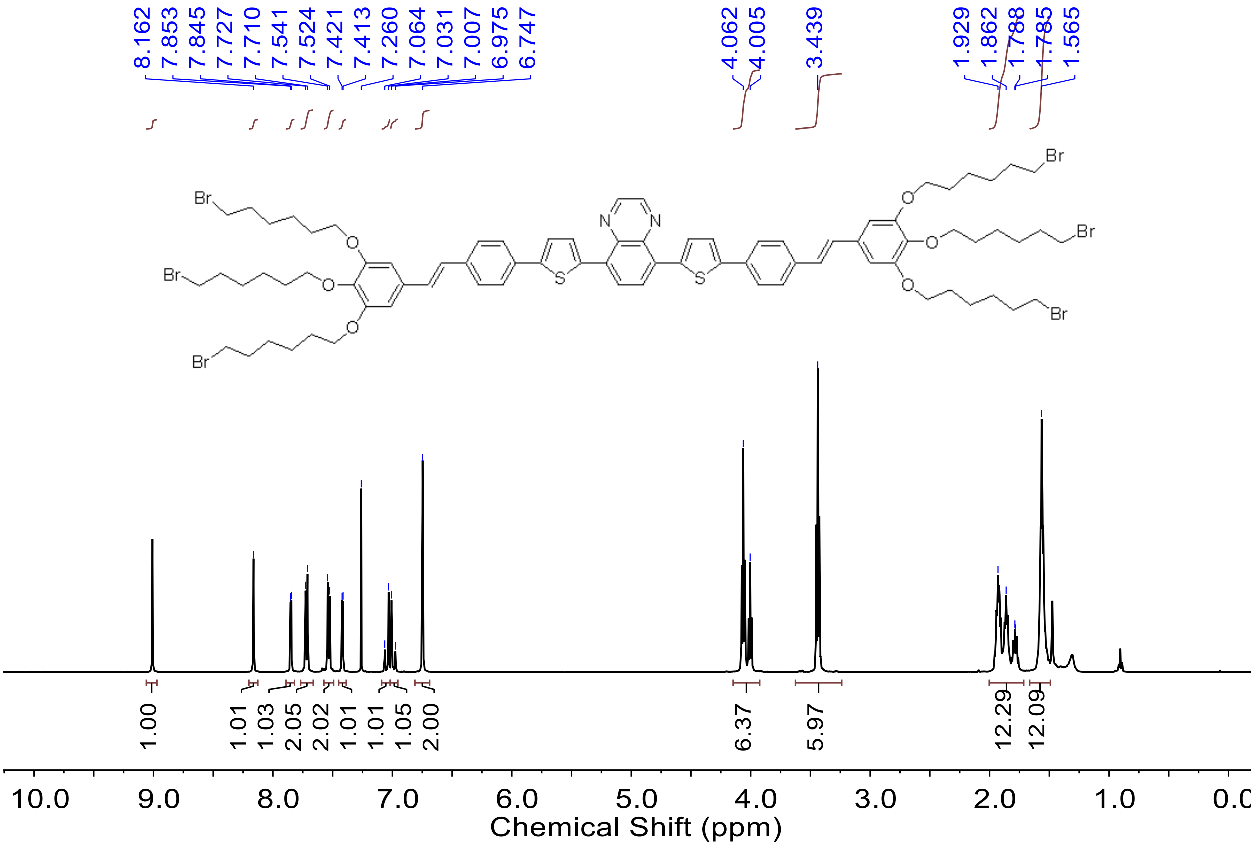

Fig. S63. Structure characterization by NMR. ^{1}H NMR spectrum of compound **9** in deuterated chloroform.

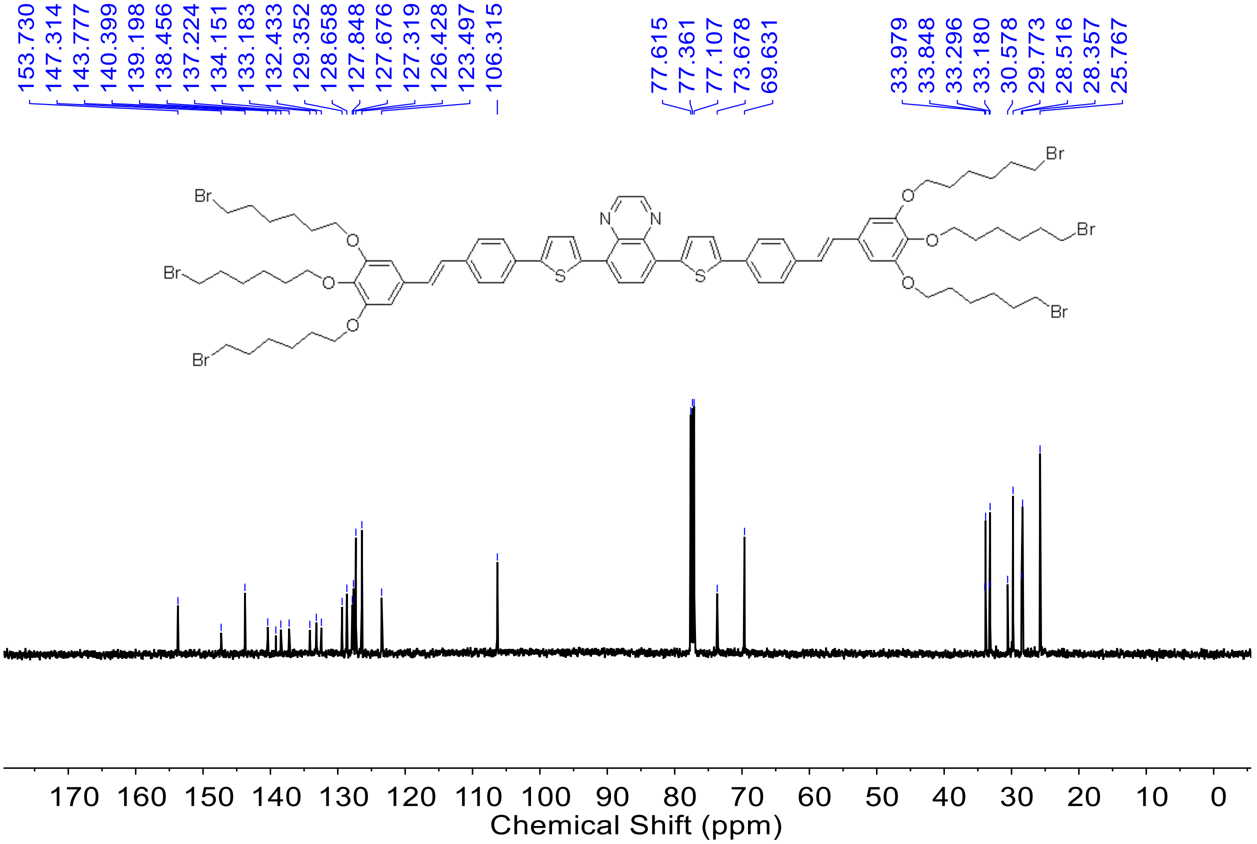

Fig. S64. Structure characterization by NMR. ^{13}C NMR spectrum of compound **9** in deuterated chloroform.

Compound **COE-QX**:

To obtain this compound, compound **9** was used for a similar quaternization reaction as described above for compound **COE-BSe**. The product **COE-QX** was afforded as a deep red solid (349 mg, 94 % yield). ^{1}H NMR (500 MHz, DMSO-*d*$_6$, 353K) δ 9.17 (s, 2H), 8.41 (s, 2H), 8.05 (d, *J* = 3.8 Hz, 2H), 7.79 (d, *J* = 8.4 Hz, 4H), 7.67 (d, *J* = 8.4 Hz, 4H), 7.63 (d, *J* = 3.6 Hz, 2H), 7.22 (s, 2H), 6.95 (s, 4H), 4.16 – 4.02 (m, 8H), 3.99 – 3.91 (m, 4H), 3.43 – 3.34 (m, 12H), 3.12 (s, 54H), 1.87 – 1.67 (m, 24H), 1.62 – 1.51 (m, 12H), 1.47 – 1.35 (m, 12H). ^{13}C NMR (126 MHz, DMSO, 373K) δ 153.28, 146.67, 144.66, 139.32, 138.78, 137.71, 137.33, 133.49, 133.03, 131.57, 129.48, 129.15, 127.71, 127.50, 126.11, 124.01, 106.79, 73.03, 69.42, 66.37, 29.98, 29.22, 26.19, 26.05, 25.50, 25.44, 22.65.

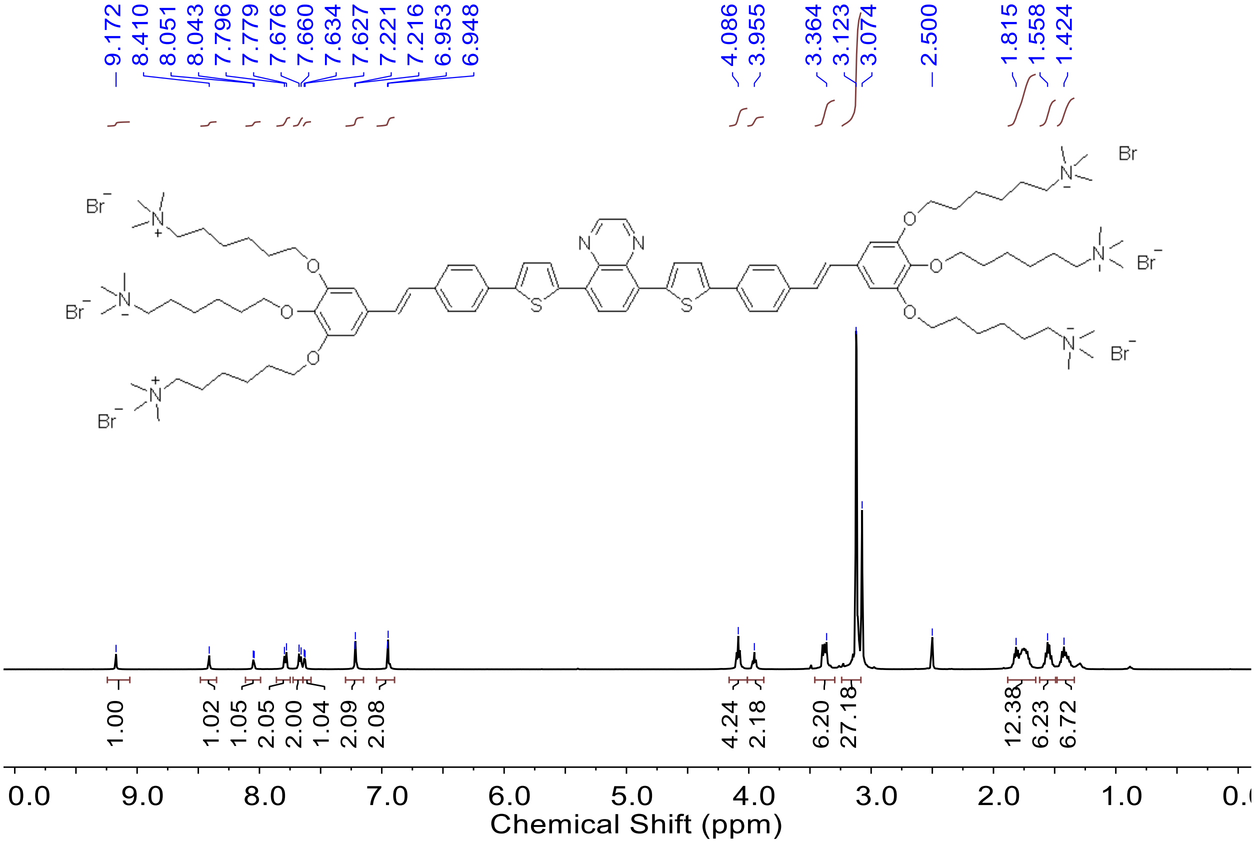

Fig. S65. Structure characterization by NMR. ^{1}H NMR spectrum of compound **COE-QX** in deuterated DMSO.

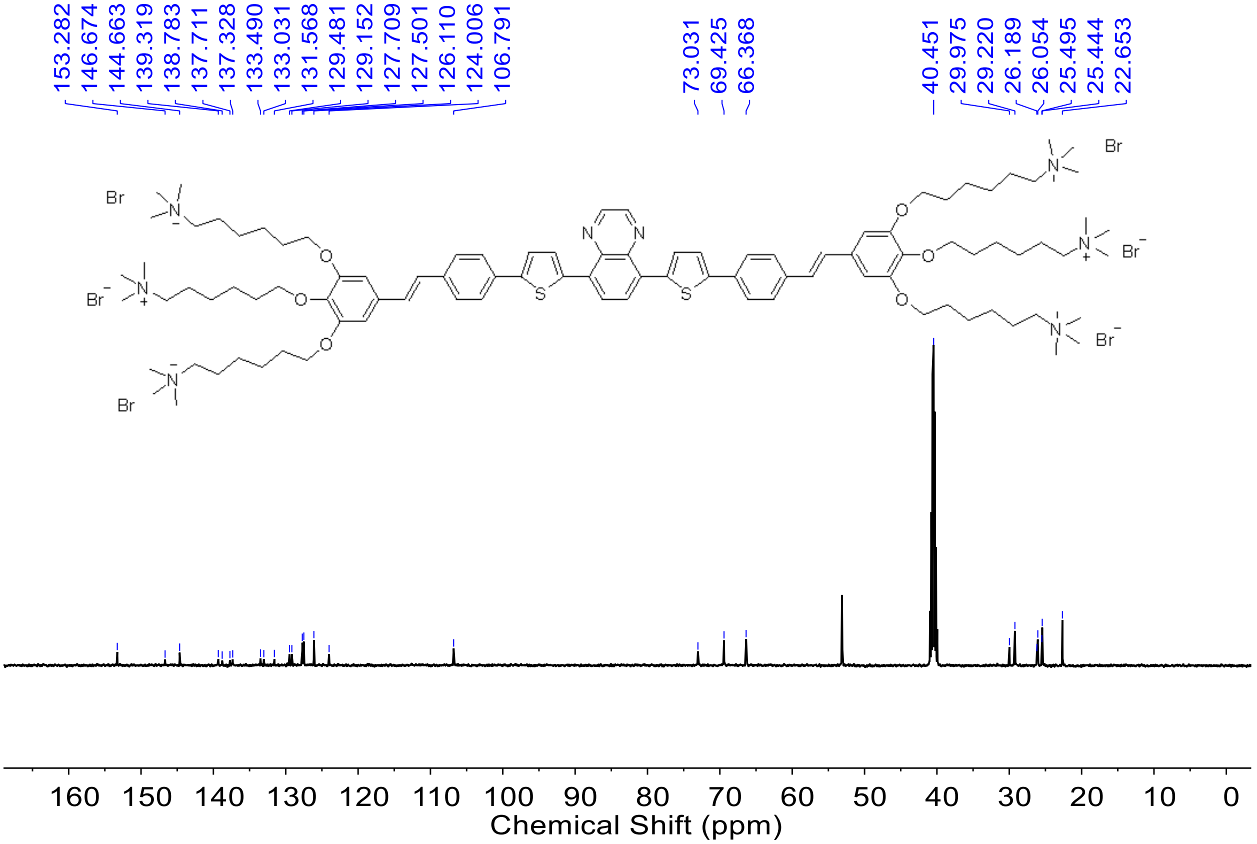

Fig. S66. Structure characterization by NMR. ^{13}C NMR spectrum of compound **COE-QX** in deuterated DMSO.

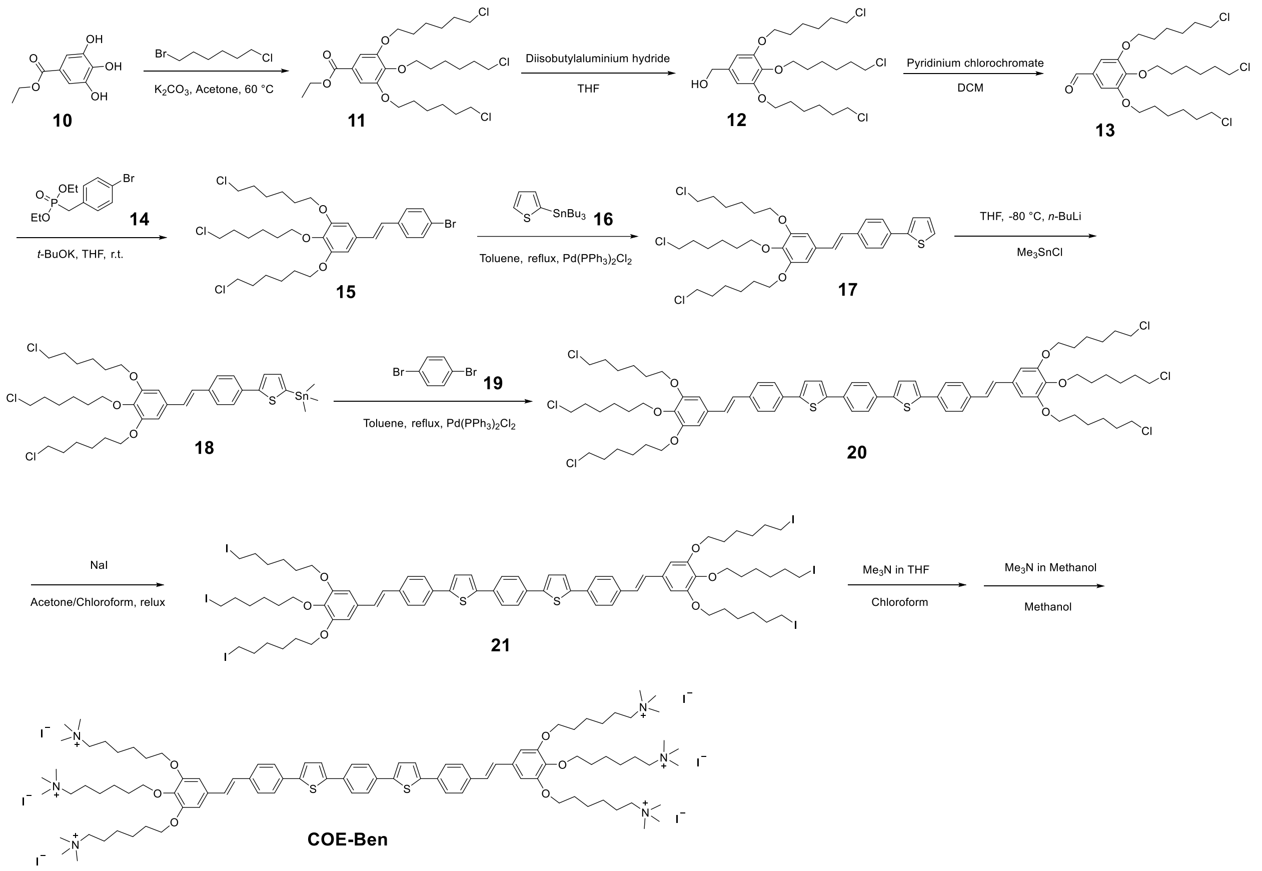

Fig. S67. Synthetic pathway. The synthetical route for **COE-Ben**.

Ethyl 3,4,5-tris((6-chlorohexyl)oxy)benzoate (compound **11**):

Ethyl Gallate (compound **10**) (10.2 g, 51 mmol), anhydrous potassium carbonate (42.5 g, 308 mmol), and 1-bromo-6-chlorohexane (51.4 g, 257 mmol) were added to a round flask and purged with Ar. Subsequently, 200 mL acetone was injected into the mixture and then heated at 70 °C for 2 days with stirring. Upon cooling to room temperature, the reaction mixture was poured into water, and extracted with ethyl acetate. The transparent organic phase was dried over Na_2SO_4 and then removed the organic solvent by rotary evaporator. The crude product was purified with silica gel column chromatography using hexane : dichloromethane : ethyl acetate = 5 : 5 : 1 as eluent, and then the product was obtained as colorless oil (27.7 g, 97 % yield). ^{1}H NMR (400 MHz, Chloroform-*d*, 300K) δ 7.28 (s, 2H), 4.38 (q, *J* = 7.1 Hz, 2H), 4.09 – 4.01 (m, 6H), 3.61 – 3.54 (m, 6H), 1.91 – 1.74 (m, 12H), 1.58 – 1.50 (m, 12H), 1.41 (t, *J* = 7.1 Hz, 3H). ^{13}C NMR (101 MHz, $CDCl_3$, 300K) δ 166.70, 153.03, 142.50, 125.60, 108.40, 73.54, 69.28, 61.38, 45.39, 45.29, 32.98, 32.89, 30.49, 29.52, 27.10, 26.97, 25.77, 25.74, 14.76.

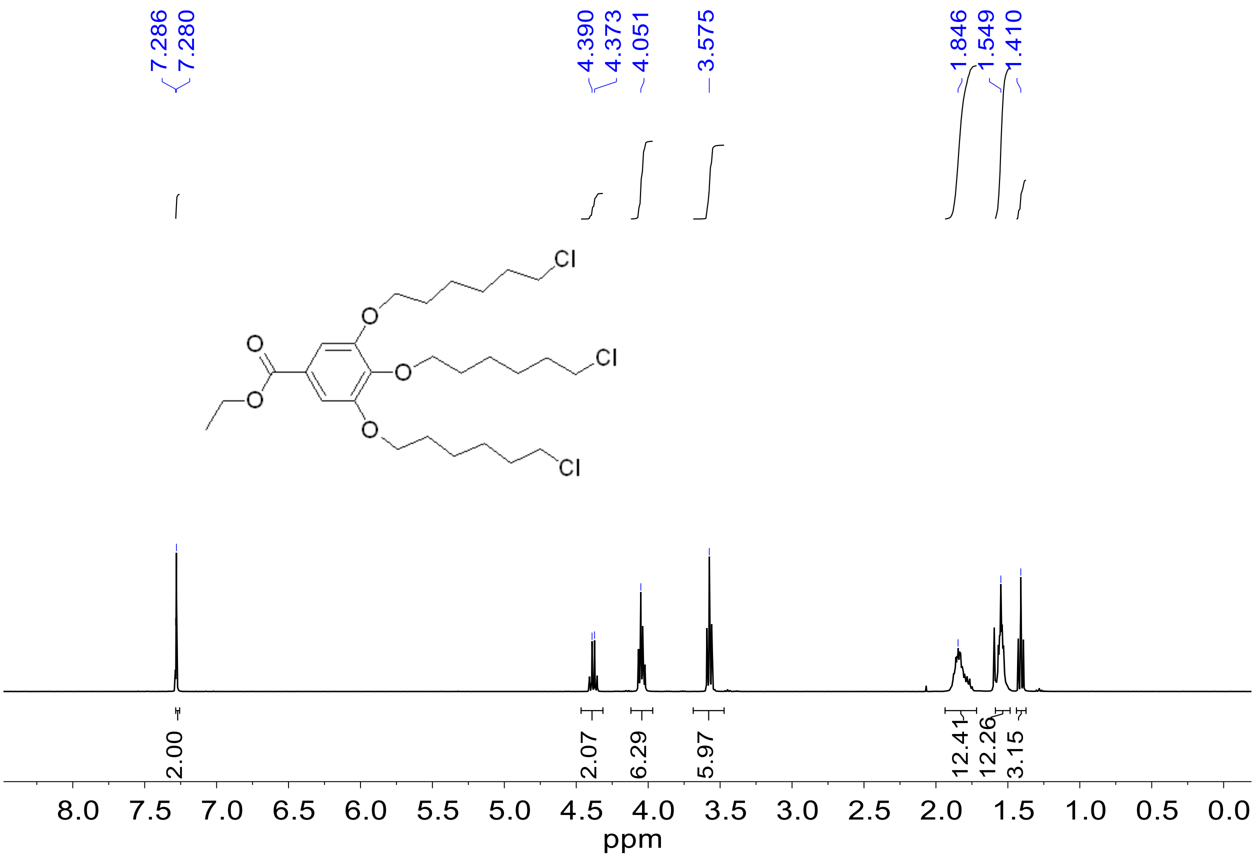

Fig. S68. Structure characterization by NMR. ^{1}H NMR spectrum of compound **11** in deuterated chloroform.

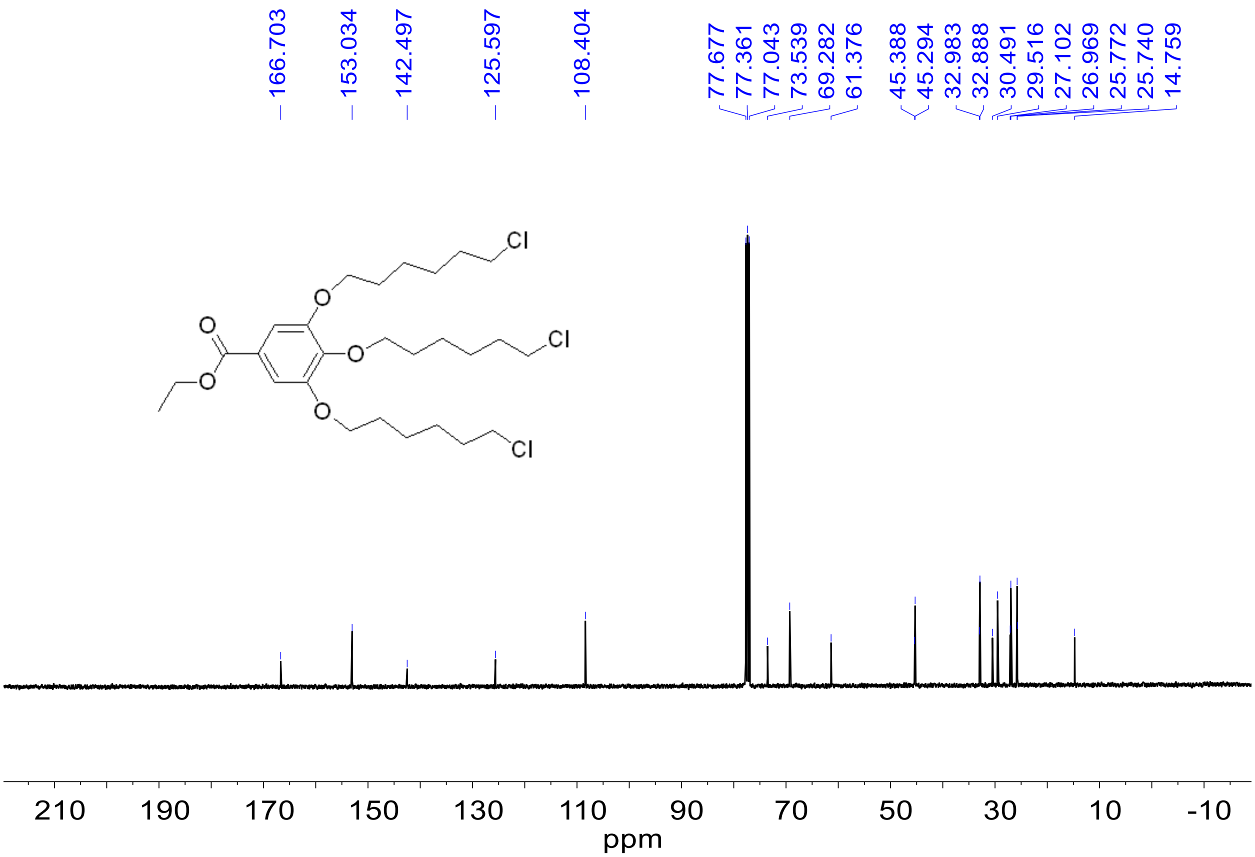

Fig. S69. Structure characterization by NMR. ^{13}C NMR spectrum of compound **11** in deuterated chloroform.

(3,4,5-Tris((6-chlorohexyl)oxy)phenyl)methanol (compound **12**):

Ethyl 3,4,5-tris((6-chlorohexyl)oxy)benzoate (compound **11**) (27.7 g, 50 mmol) was added to a round flask and purged with argon, then 150 mL dry THF was added under the protection of argon. After cooled down to –80 °C, diisobutylaluminium hydride (DIBAL) solution in THF (1 M, 150 mL) was added dropwise into the reaction mixture and stirred for another 2 hours at –80 °C, then allowed to warm up to room temperature and stirred for another 16 hours. The reaction mixture was cooled down using ice/water bath, and water was slowly added dropwise to quench the reaction. Then, the reaction mixture was washed with saline water (NaCl) and extracted with ethyl acetate. The transparent organic phase was dried over Na_2SO_4 and then removed the organic solvent by rotary evaporator. The crude product was purified with silica gel column chromatography using hexane : ethyl acetate = 5 : 1 as eluent, and then the final product was obtained as colorless oil (23.7 g, 93 % yield). ^1H NMR (400 MHz, Chloroform-*d*, 300K) δ 6.56 (s, 2H), 4.59 (d, *J* = 5.9 Hz, 2H), 4.02 – 3.89 (m, 6H), 3.62 – 3.47 (m, 6H), 1.86 – 1.71 (m, 12H), 1.56 – 1.45 (m, 12H). ^{13}C NMR (101 MHz, $CDCl_3$, 300K) δ 153.50, 137.78, 136.56, 105.73, 73.46, 69.19, 65.92, 45.42, 45.32, 33.00, 32.89, 30.47, 29.60, 27.14, 26.96, 25.80, 25.77.

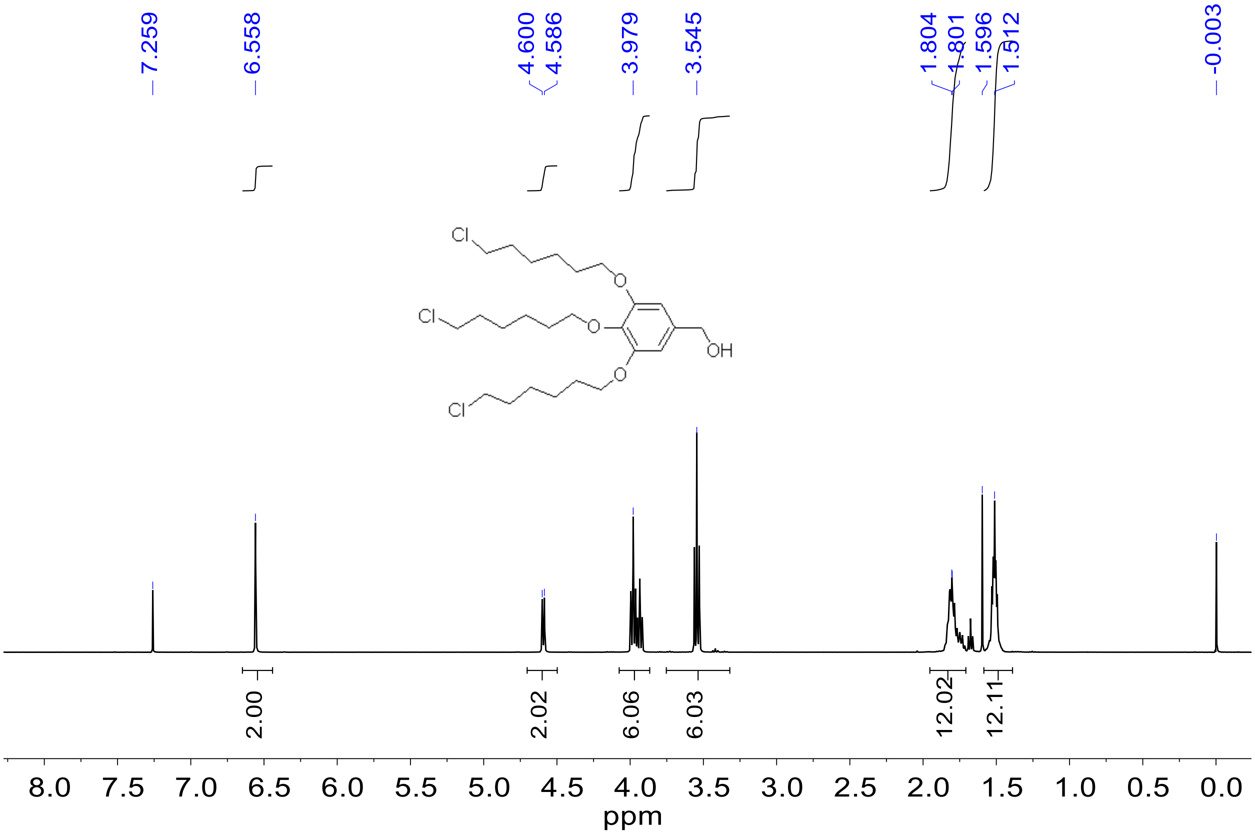

Fig. S70. Structure characterization by NMR. ^{1}H NMR spectrum of compound **12** in deuterated chloroform.

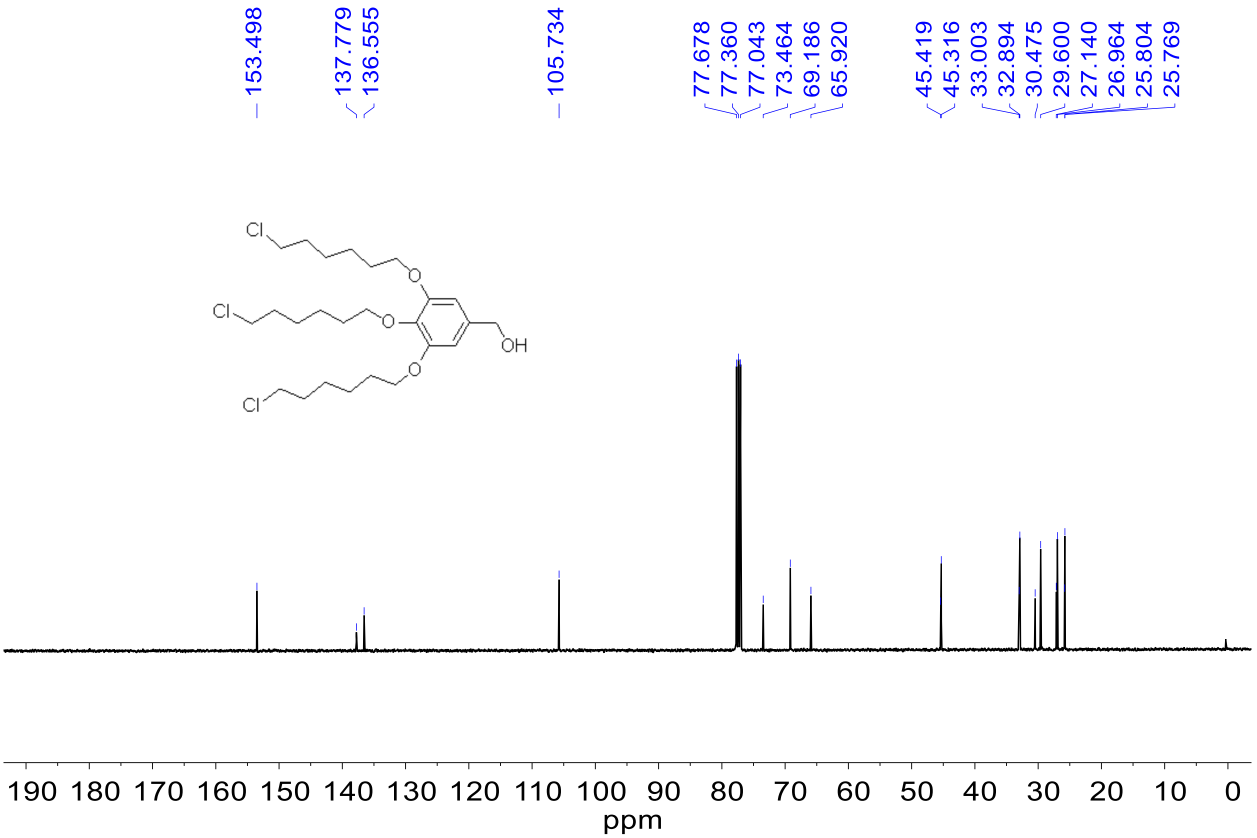

Fig. S71. Structure characterization by NMR. ^{13}C NMR spectrum of compound **12** in deuterated chloroform.

3,4,5-Tris((6-chlorohexyl)oxy)benzaldehyde (compound **13**):
(3,4,5-Tris((6-chlorohexyl)oxy)phenyl)methanol (compound **12**) (19.4 g, 37.9 mmol) was dissolved into 50 mL dichloromethane, and then pyridinium chlorochromate (16.3 g, 75.8 mmol) was added to the reaction mixture and stirred for 2 hours at room temperature. The reaction mixture was applied to silica gel column chromatography directly and purified using dichloromethane as eluent, and then the final product was obtained as colorless oil (17.1 g, 89 % yield). ^{1}H NMR (400 MHz, Chloroform-*d*, 300K) δ 9.83 (s, 1H), 7.08 (s, 2H), 4.10 – 3.99 (m, 6H), 3.60 – 3.49 (m, 6H), 1.91 – 1.72 (m, 12H), 1.57 – 1.47 (m, 12H). ^{13}C NMR (101 MHz, $CDCl_3$, 300K) δ 191.50, 153.77, 144.01, 131.92, 108.24, 73.69, 69.35, 45.37, 45.27, 32.96, 32.86, 30.51, 29.46, 27.07, 26.95, 25.76, 25.70.

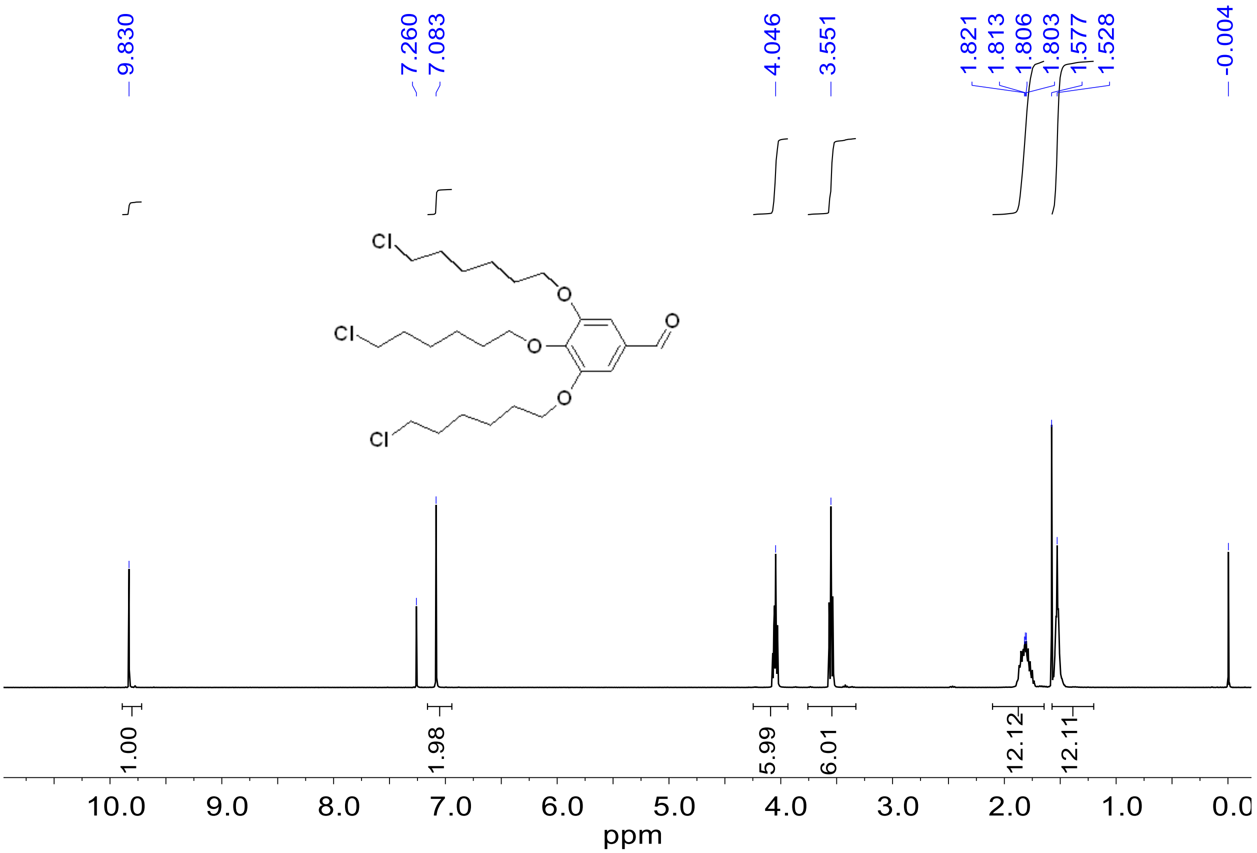

Fig. S72. Structure characterization by NMR. ^{1}H NMR spectrum of compound **13** in deuterated chloroform.

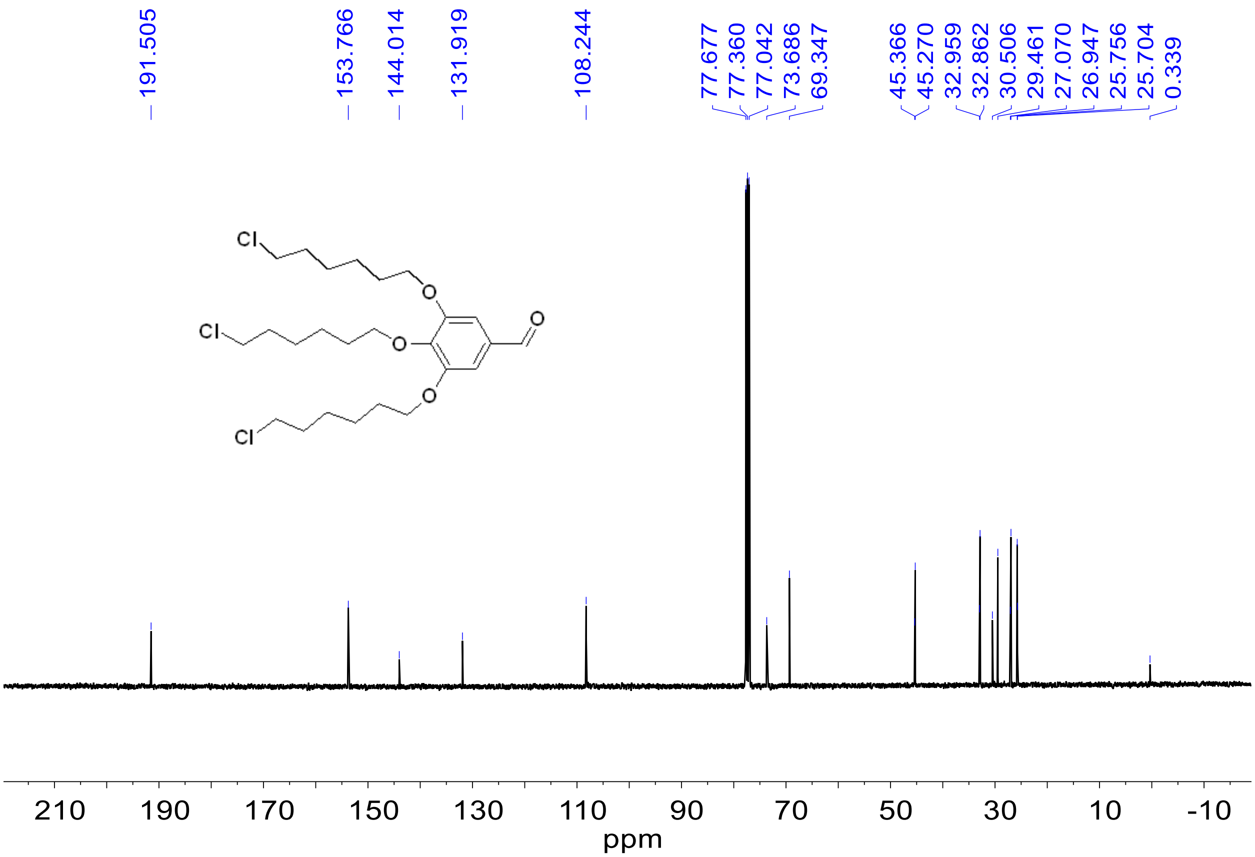

Fig. S73. Structure characterization by NMR. ^{13}C NMR spectrum of compound **13** in deuterated chloroform.

(*E*)-5-(4-bromostyryl)-1,2,3-tris((6-chlorohexyl)oxy)benzene (compound **15**):
3,4,5-Tris((6-chlorohexyl)oxy)benzaldehyde (compound **13**) (7.11 g, 13.9 mmol), diethyl (4-bromobenzyl)phosphonate (compound **14**) (6.42 g, 20.9 mmol), potassium *tert*-butoxide (2.03 g, 18.1 mmol), and 100 mL dry THF were added to a round flask under the protection of argon atmosphere. After 20 hours reaction under stirring at room temperature, the reaction mixture was poured into water, washed using NH_4Cl aqueous solution, and extracted with dichloromethane. The transparent organic phase was dried over Na_2SO_4 and then removed the organic solvent by rotary evaporator. The crude product was purified with column chromatography using hexane : dichloromethane = 1 : 1as eluent, and then the product was obtained as white solid (8.50 g, 92 % yield). ^{1}H NMR (400 MHz, Chloroform-*d*, 300K) δ 7.47 (d, *J* = 8.5 Hz, 2H), 7.35 (d, *J* = 8.5 Hz, 2H), 6.99 (d, *J* = 16.2 Hz, 1H), 6.89 (d, *J* = 16.2 Hz, 1H), 6.70 (s, 2H), 4.07 – 3.94 (m, 6H), 3.61 – 3.51 (m, 6H), 1.91 – 1.72 (m, 12H), 1.57 – 1.46 (m, 12H). ^{13}C NMR (101 MHz, $CDCl_3$, 300K) δ 153.56, 138.70, 136.62, 132.68, 132.11, 129.83, 128.17, 126.93, 121.49, 105.66, 73.57, 69.28, 45.41, 45.32, 33.00, 32.89, 30.49, 29.63, 27.14, 26.98, 25.79.

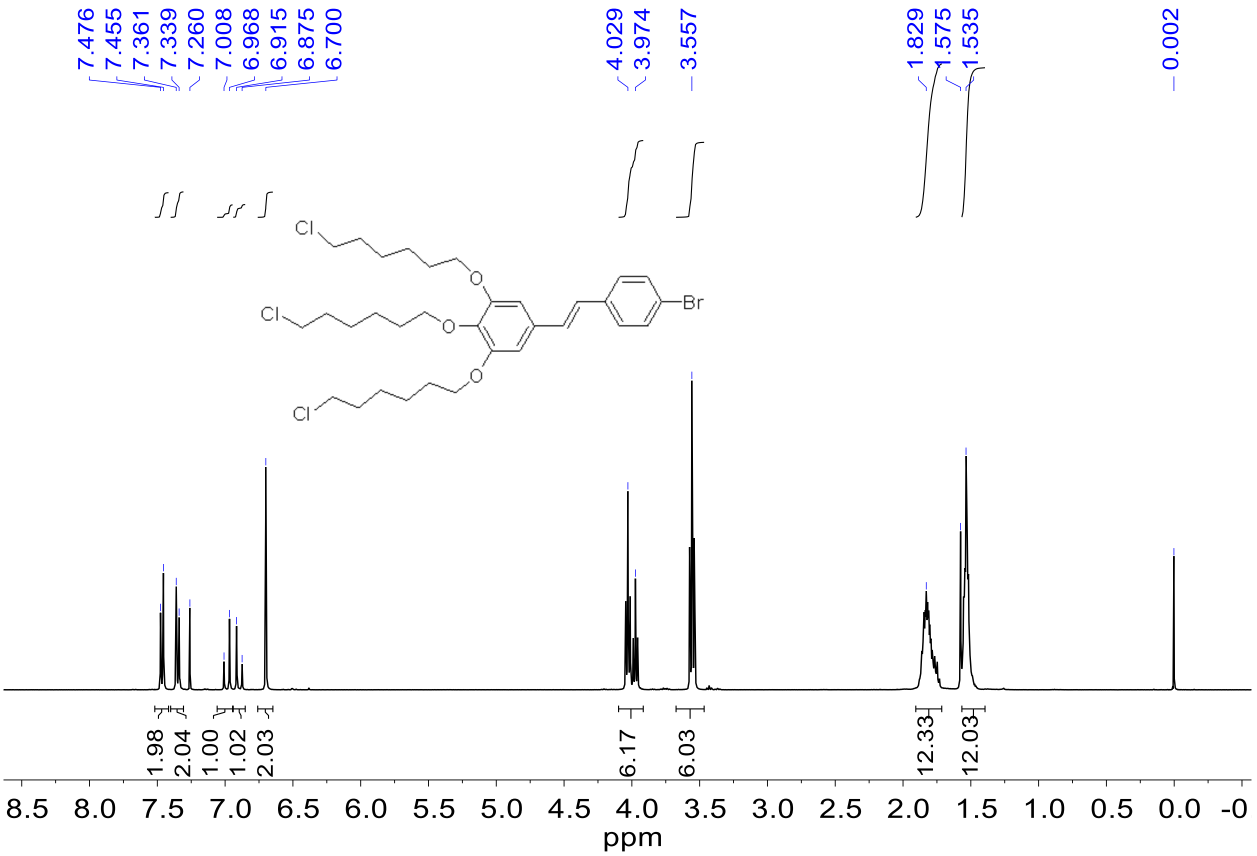

Fig. S74. Structure characterization by NMR. ^{1}H NMR spectrum of compound **15** in deuterated chloroform.

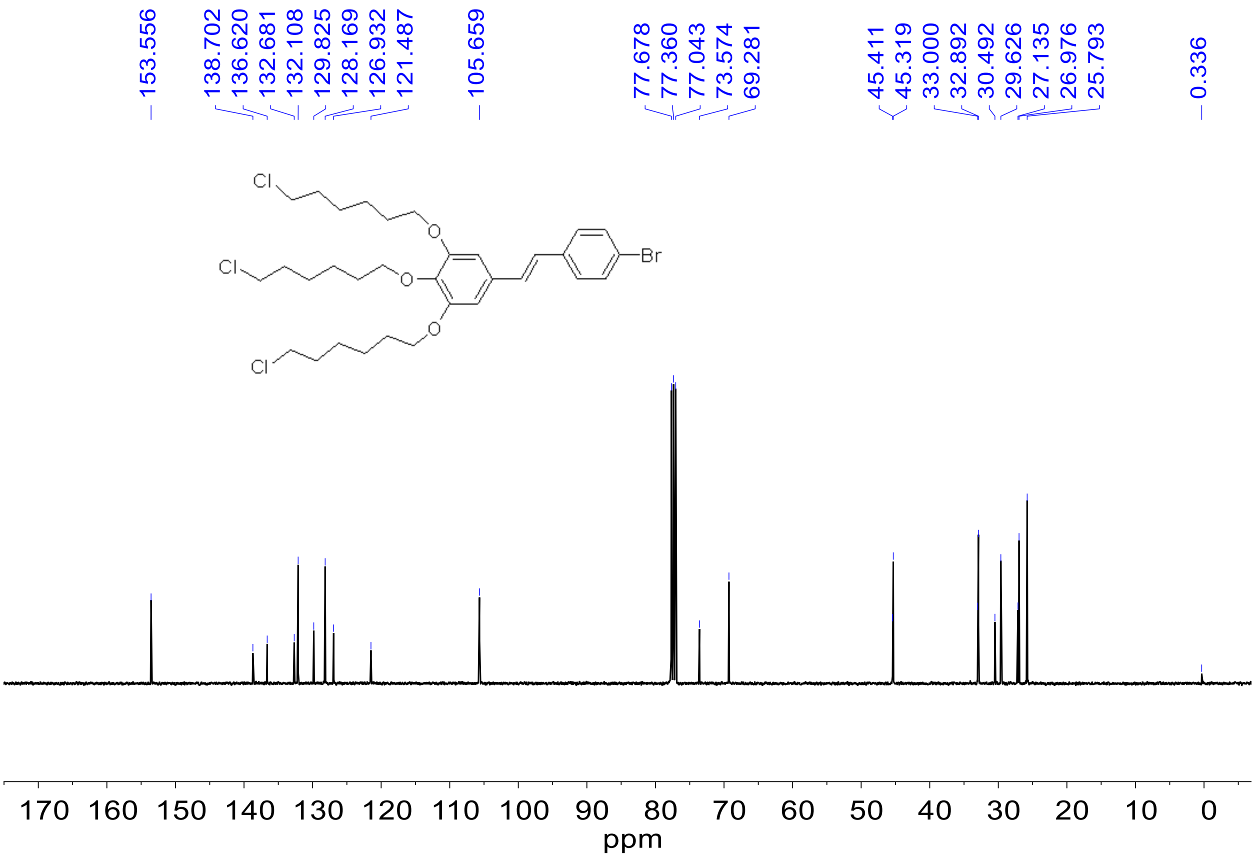

Fig. S75. Structure characterization by NMR. ^{13}C NMR spectrum of compound **15** in deuterated chloroform.

(*E*)-2-(4-(3,4,5-tris((6-chlorohexyl)oxy)styryl)phenyl)thiophene (compound **17**): (*E*)-5-(4-bromostyryl)-1,2,3-tris((6-chlorohexyl)oxy)benzene (compound **15**) (1.21 g, 1.83 mmol), tributyl(thiophen-2-yl)stannane (compound **16**) (1.02 g, 2.74 mmol), catalyst $Pd(PPh_3)_4$ (42.3 mg, 0.0366 mmol) were added to a round flask under the protection of nitrogen atmosphere. After purged using nitrogen, 20 mL dry toluene and 5 mL dry dimethyl formamide were added to the reaction mixture, which was then heated at 120 °C for 16 hours with stirring. After being cooled to room temperature, the reaction mixture was poured into water, and extracted with dichloromethane. The organic phase was dried over Na_2SO_4 and then the organic solvent was removed by rotary evaporator. The crude product was purified with silica gel column chromatography using hexane : dichloromethane = 1 : 1 as eluent, and the product was obtained as light yellow solid (1.04 g, 86 % yield). ^{1}H NMR (400 MHz, Chloroform-*d*, 300K) δ 7.60 (d, *J* = 8.4 Hz, 2H), 7.50 (d, *J* = 8.5 Hz, 2H), 7.33 (dd, *J* = 3.6, 1.2 Hz, 1H), 7.28 (dd, *J* = 5.1, 1.1 Hz, 1H), 7.09 (dd, *J* = 5.1, 3.6 Hz, 1H), 7.03 (d, *J* = 16.2 Hz, 1H), 6.97 (d, *J* = 16.2 Hz, 1H), 6.72 (s, 2H), 4.08 – 3.94 (m, 6H), 3.60 – 3.51 (m, 6H), 1.91 – 1.72 (m, 12H), 1.59 – 1.47 (m, 12H). ^{13}C NMR (101 MHz, $CDCl_3$, 300K) δ 153.56, 144.48, 138.59, 136.86, 133.85, 133.03, 129.12, 128.44,

127.64, 127.20, 126.46, 125.15, 123.34, 105.65, 73.60, 69.30, 45.43, 45.34, 33.03, 32.92, 30.52, 29.66, 27.16, 27.01, 25.82.

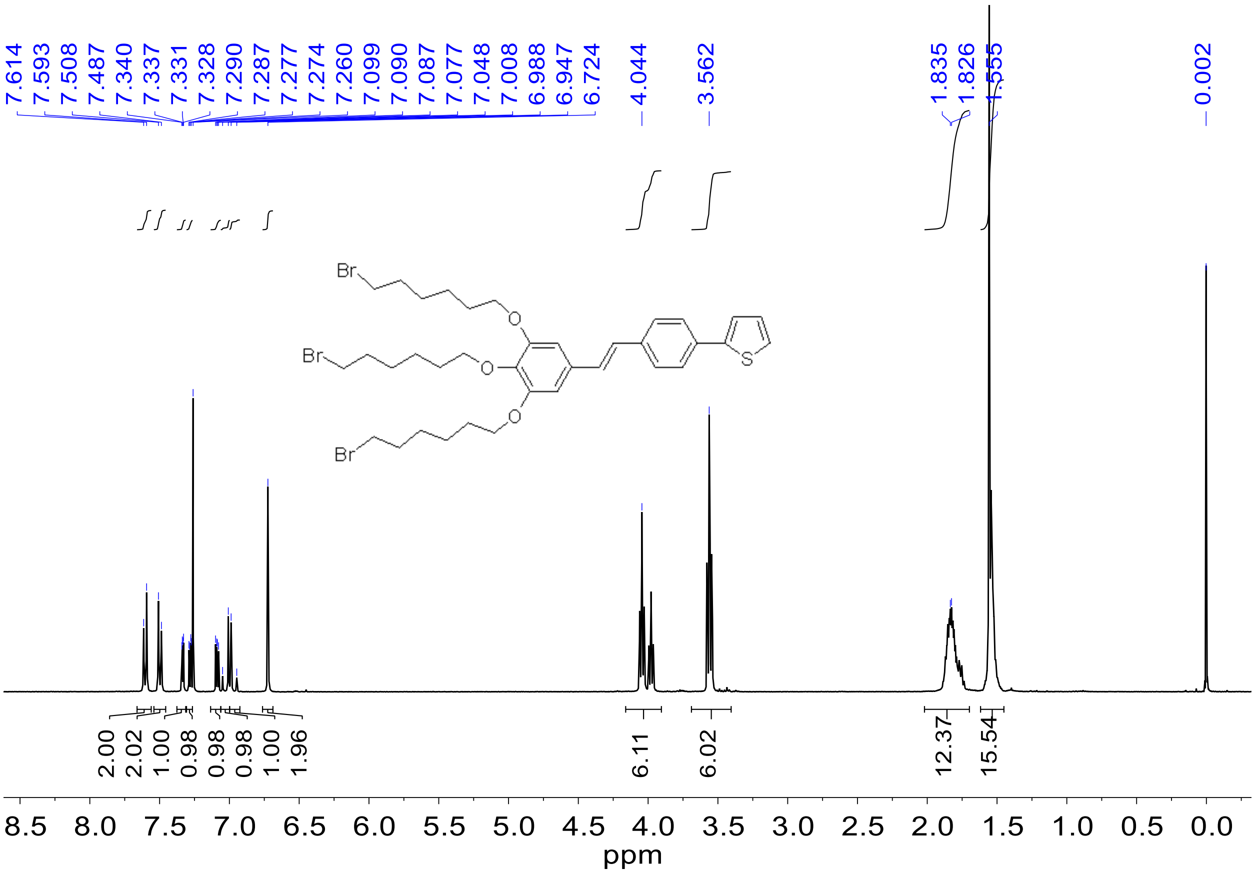

Fig. S76. Structure characterization by NMR. ^{1}H NMR spectrum of compound **17** in deuterated chloroform.

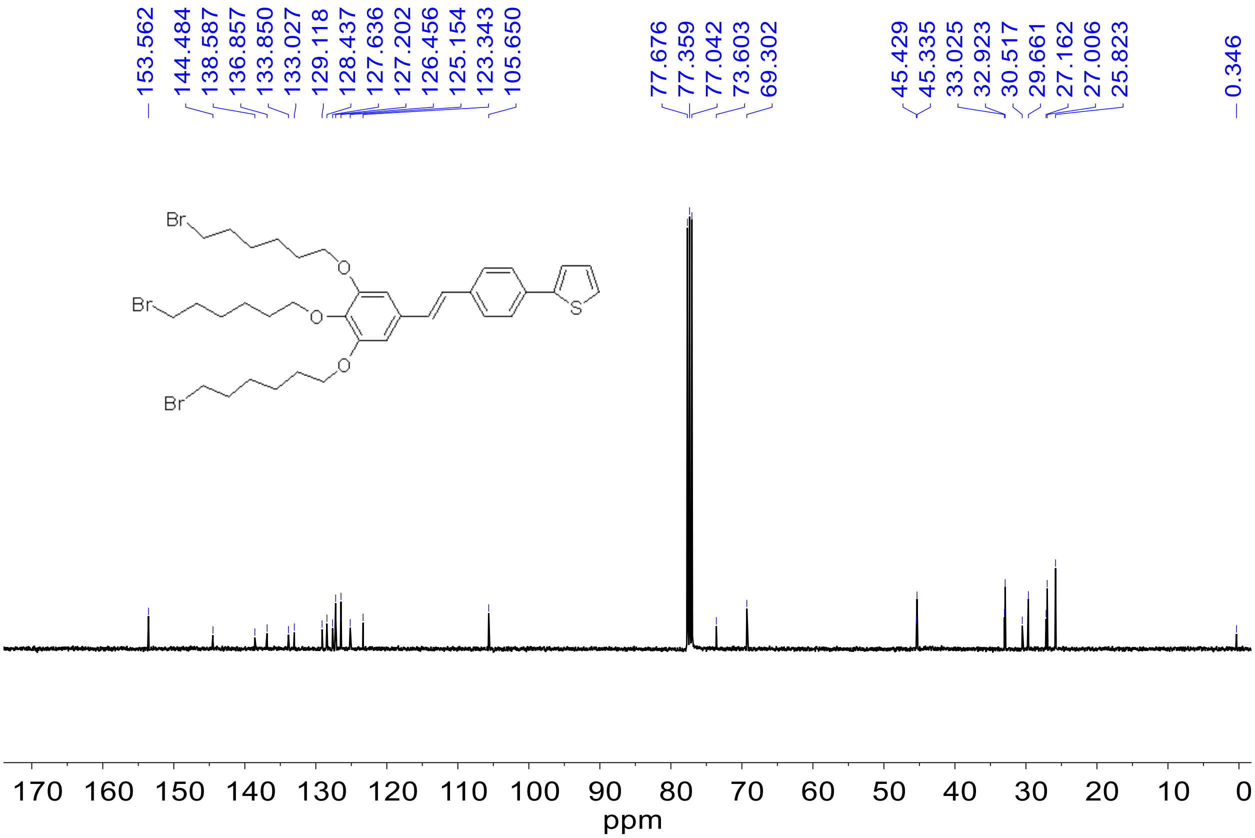

Fig. S77. Structure characterization by NMR. ^{13}C NMR spectrum of compound **17** in deuterated chloroform.

(*E*)-trimethyl(5-(4-(3,4,5-tris((6-chlorohexyl)oxy)styryl)phenyl)thiophen-2-yl)stannane (compound **18**):

(*E*)-2-(4-(3,4,5-tris((6-chlorohexyl)oxy)styryl)phenyl)thiophene (compound **17**) (1.04 g, 1.56 mmol) was added to a flask and purged using argon, and then 20 mL dry THF was added under argon protection. The mixture was cooled down to –80°C utilizing an ultralow temperature reaction bath. Then, 1.6 mL *n*-butyllithium solution in cyclohexane (2 M, 3.2 mmol) was added drop-wise into the reaction mixture. After stirring at –80°C for 2 hours, 4.7 mL trimethyltin chloride solution in hexane (1 M, 4.7 mmol) was added and then the reaction mixture was warmed up to room temperature. After being stirred at room temperature overnight (about 16 hours), the reaction mixture was poured into water and extracted with hexane. The organic phase was washed using water four times and then dried over Na_2SO_4. After the solvent being removed using rotary evaporator and vacuum pump, the crude product was obtained as a yellow oil, which will be used in subsequent reactions without further purification (1.03 g, 80 % yield). ^{1}H NMR (400 MHz, Chloroform-*d*, 300K) δ 7.61 (d, *J* = 8.4 Hz, 2H), 7.49 (d, *J* = 8.4 Hz, 2H), 7.44 (d, *J* = 3.3 Hz, 1H), 7.18 (d, *J* = 3.4 Hz, 1H), 7.02 (d, *J* = 16.3 Hz, 1H), 6.96 (d, *J* = 16.3 Hz, 1H), 6.72 (s, 2H), 4.08 – 3.94 (m, 6H), 3.60 – 3.52 (m, 6H), 1.90 – 1.73 (m, 12H), 1.58 – 1.47 (m, 22H), 0.40 (s, 9H). ^{13}C NMR (101 MHz, $CDCl_3$, 300K) δ 153.55, 150.19, 138.51, 138.18, 136.61, 136.60, 133.97, 133.08, 128.93, 127.73, 127.17, 126.47, 124.59, 105.58, 73.59, 69.27, 45.44, 45.35, 33.02, 32.92, 30.52, 29.66, 27.16, 27.01, 25.82, -7.87.

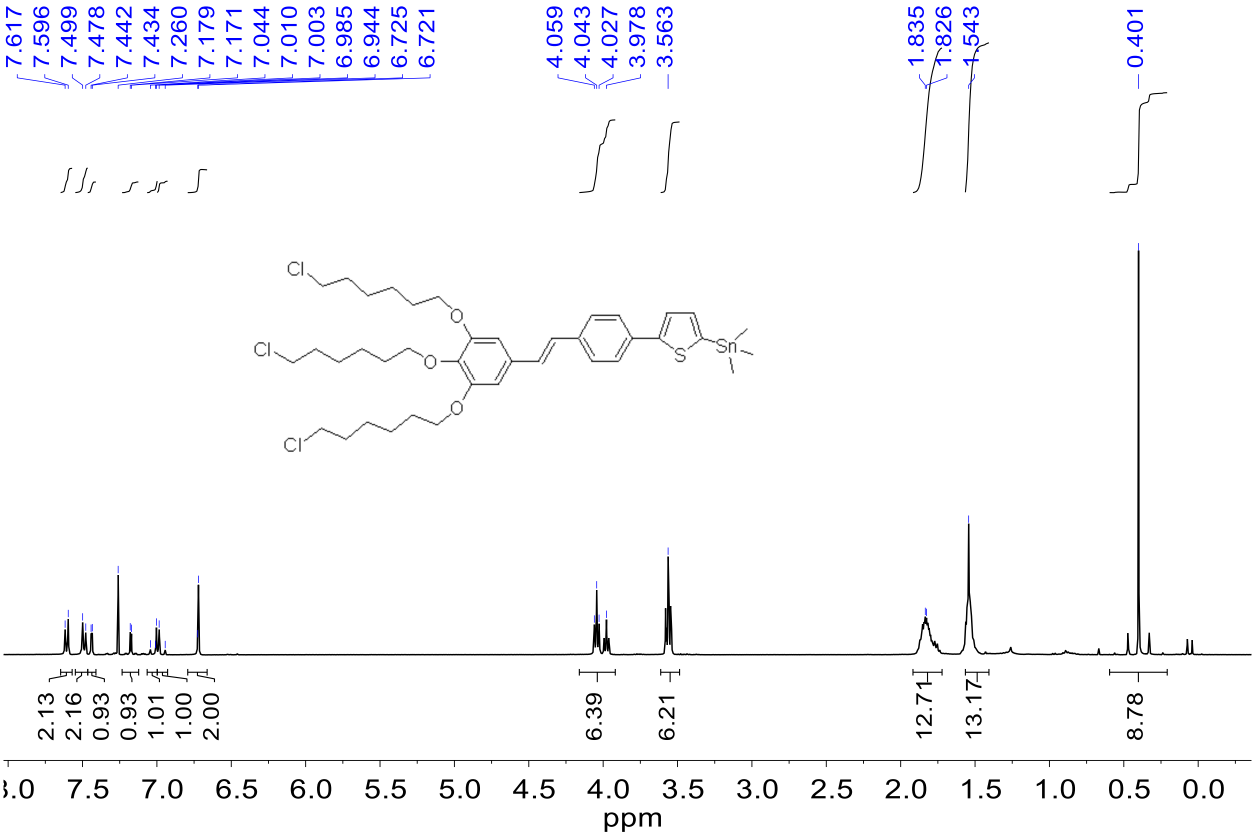

Fig. S78. Structure characterization by NMR. ^{1}H NMR spectrum of compound **18** in deuterated chloroform.

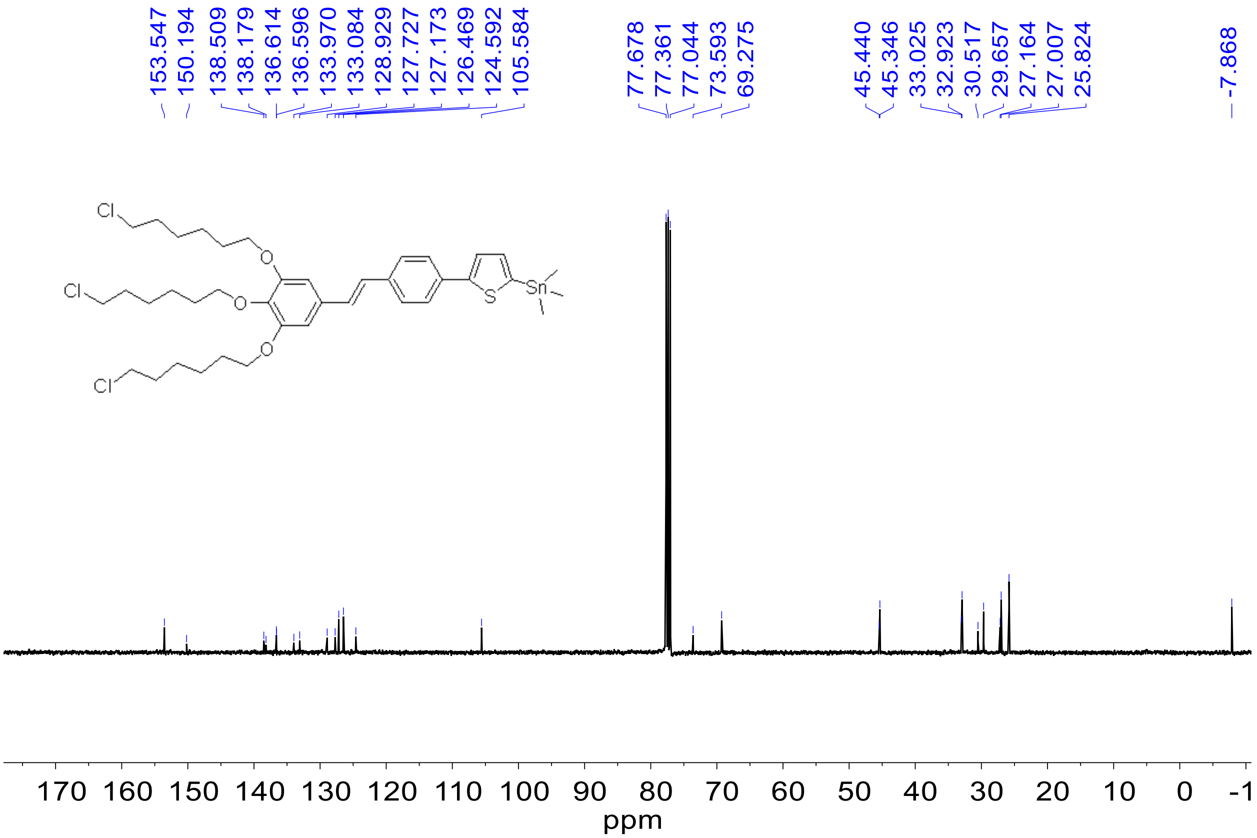

Fig. S79. Structure characterization by NMR. ^{13}C NMR spectrum of compound **18** in deuterated chloroform.

1,4-Bis(5-(4-((*E*)-3,4,5-tris((6-chlorohexyl)oxy)styryl)phenyl)thiophen-2-yl)benzene (compound **20**):

This compound was synthesized according to a similar procedure as described for compound **3** above, but the reactant (*E*)-trimethyl(5-(4-(3,4,5-tris((6-bromohexyl)oxy)styryl)phenyl)thiophen-2-yl)stannane (compound **1**) was replaced to (*E*)-trimethyl(5-(4-(3,4,5-tris((6-chlorohexyl)oxy)styryl)phenyl)thiophen-2-yl)stannane (compound **18**), the reactant 4,7-dibromobenzo[c][1,2,5]selenadiazole (compound **2**) was replaced to 1,4-dibromobenzene (compound **19**). The final product was obtained as a yellow solid (810 mg, 78 % yield). ^{1}H NMR (500 MHz, Chloroform-*d*, 298K) δ 7.65 (s, 4H), 7.63 (d, *J* = 8.3 Hz, 4H), 7.52 (d, *J* = 8.4 Hz, 4H), 7.33 (s, 4H), 7.04 (d, *J* = 16.1 Hz, 2H), 6.98 (d, *J* = 16.2 Hz, 2H), 6.73 (s, 4H), 4.09 – 4.02 (m, 8H), 4.01 – 3.95 (m, 4H), 3.62 – 3.52 (m, 12H), 1.91 – 1.74 (m, 24H), 1.62 – 1.48 (m, 24H). ^{13}C NMR (126 MHz, $CDCl_3$, 298K) δ 153.55, 143.80, 143.35, 138.55, 136.97, 133.70, 133.62, 132.99, 129.19, 127.57, 127.25, 126.29, 126.12, 124.52, 124.41, 105.59, 73.60, 69.27, 45.45, 45.36, 33.02, 32.91, 30.51, 29.65, 27.16, 27.00, 25.82.

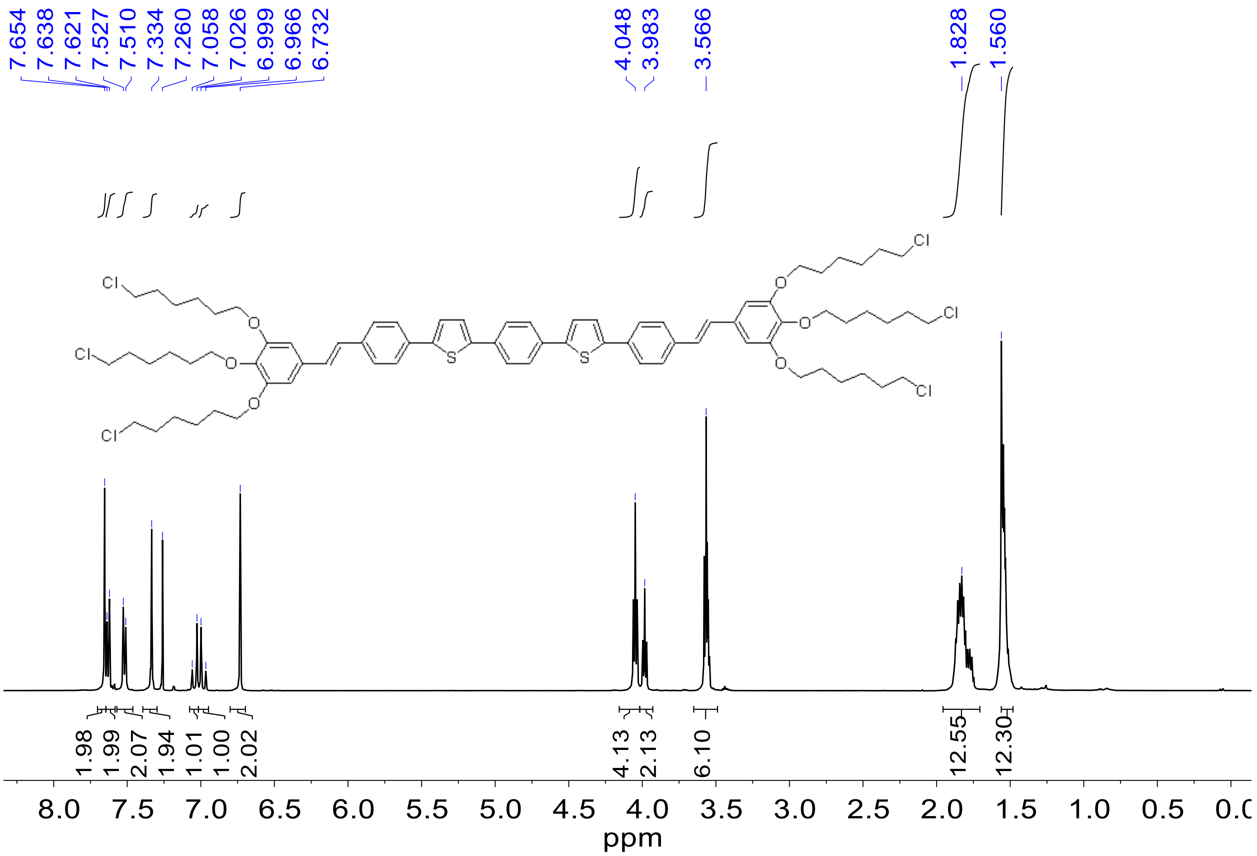

Fig. S80. Structure characterization by NMR. ^{1}H NMR spectrum of compound **20** in deuterated chloroform.

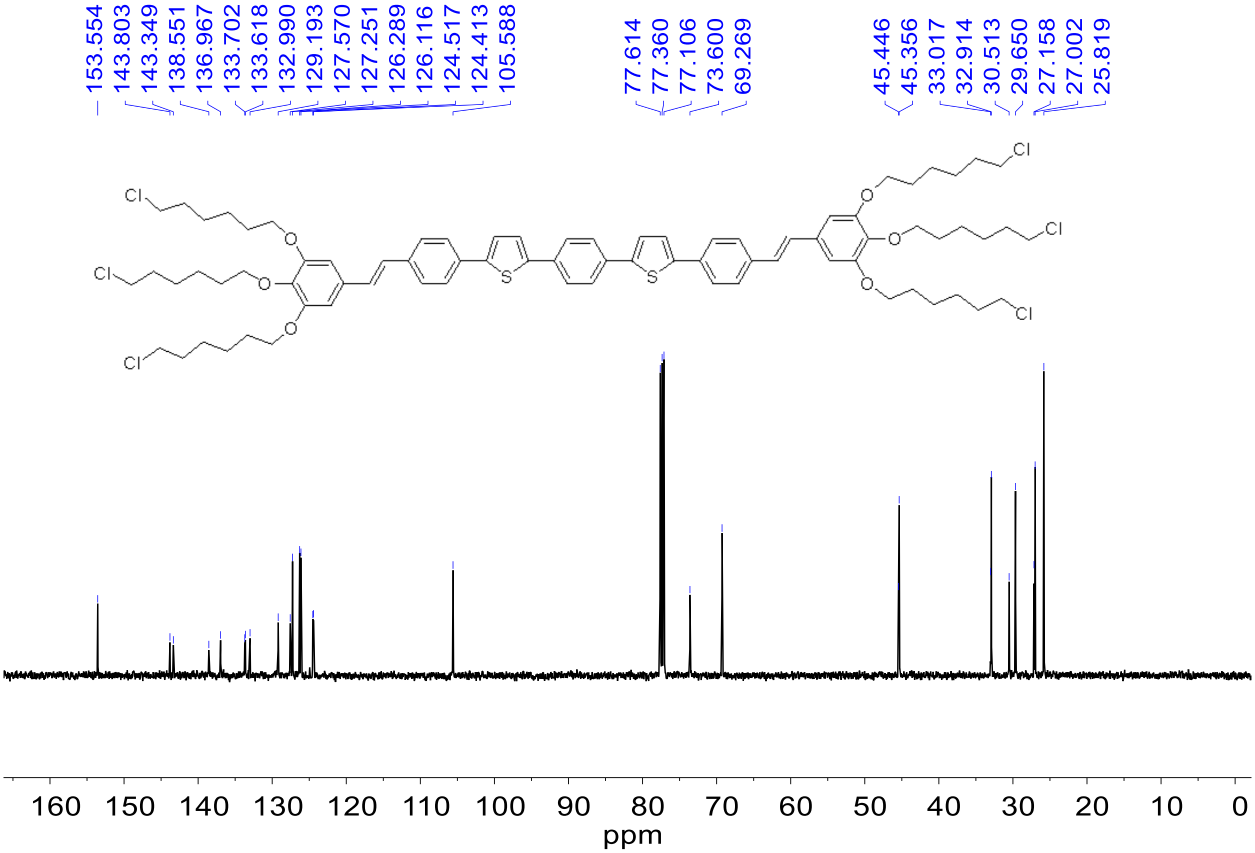

Fig. S81. Structure characterization by NMR. ^{13}C NMR spectrum of compound **20** in deuterated chloroform.

1,4-Bis(5-(4-((*E*)-3,4,5-tris((6-iodohexyl)oxy)styryl)phenyl)thiophen-2-yl)benzene (compound **21**):

1,4-Bis(5-(4-((*E*)-3,4,5-tris((6-chlorohexyl)oxy)styryl)phenyl)thiophen-2-yl)benzene (compound **20**) (0.51 g, 0.356 mmol) and excessive sodium iodide (30 g, 200 mmol) were added to a round flask and purged with argon. Subsequently, 350 mL acetone and 50 mL chloroform were injected into the mixture and then stirred at reflux for 3 days. Upon cooling to room temperature, most of solvent was removed by rotary evaporator. Then, the reaction mixture was poured into water, and extracted with chloroform. The transparent organic phase was dried over Na_2SO_4 and then removed the organic solvent by rotary evaporator. The crude product was purified with silica gel column chromatography using hexane : dichloromethane = 1 : 5 as eluent, and then the product was obtained as yellow solid (0.62 g, 87 % yield). ^{1}H NMR (500 MHz, Chloroform-*d*, 298K) δ 7.65 (s, 4H), 7.63 (d, *J* = 8.4 Hz, 4H), 7.52 (d, *J* = 8.4 Hz, 4H), 7.33 (s, 4H), 7.04 (d, *J* = 16.2 Hz, 2H), 6.98 (d, *J* = 16.1 Hz, 2H), 6.73 (s, 4H), 4.07 – 4.02 (m, 8H), 4.00 – 3.95 (m, 4H), 3.26 – 3.18 (m, 12H), 1.93 – 1.72 (m, 24H), 1.60 – 1.44 (m, 24H). ^{13}C NMR (126 MHz, $CDCl_3$, 298K) δ 153.54, 143.80, 143.35, 138.54, 136.96, 133.70, 133.62, 132.99, 129.19, 127.58, 127.26, 126.29,

126.11, 124.52, 124.41, 105.60, 73.61, 69.27, 33.96, 33.80, 30.80, 30.62, 30.49, 29.61, 25.51, 25.48, 7.53, 7.34.

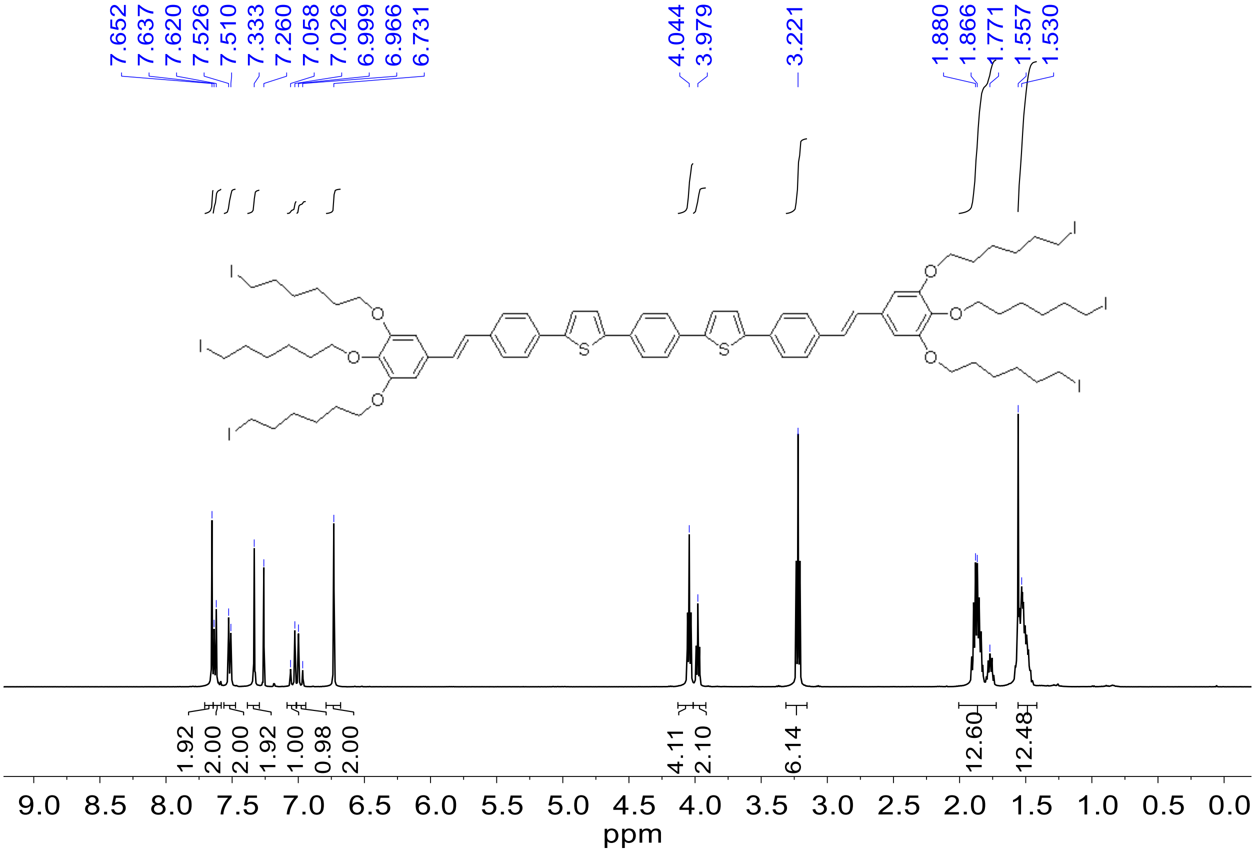

Fig. S82. Structure characterization by NMR. ^{1}H NMR spectrum of compound **21** in deuterated chloroform.

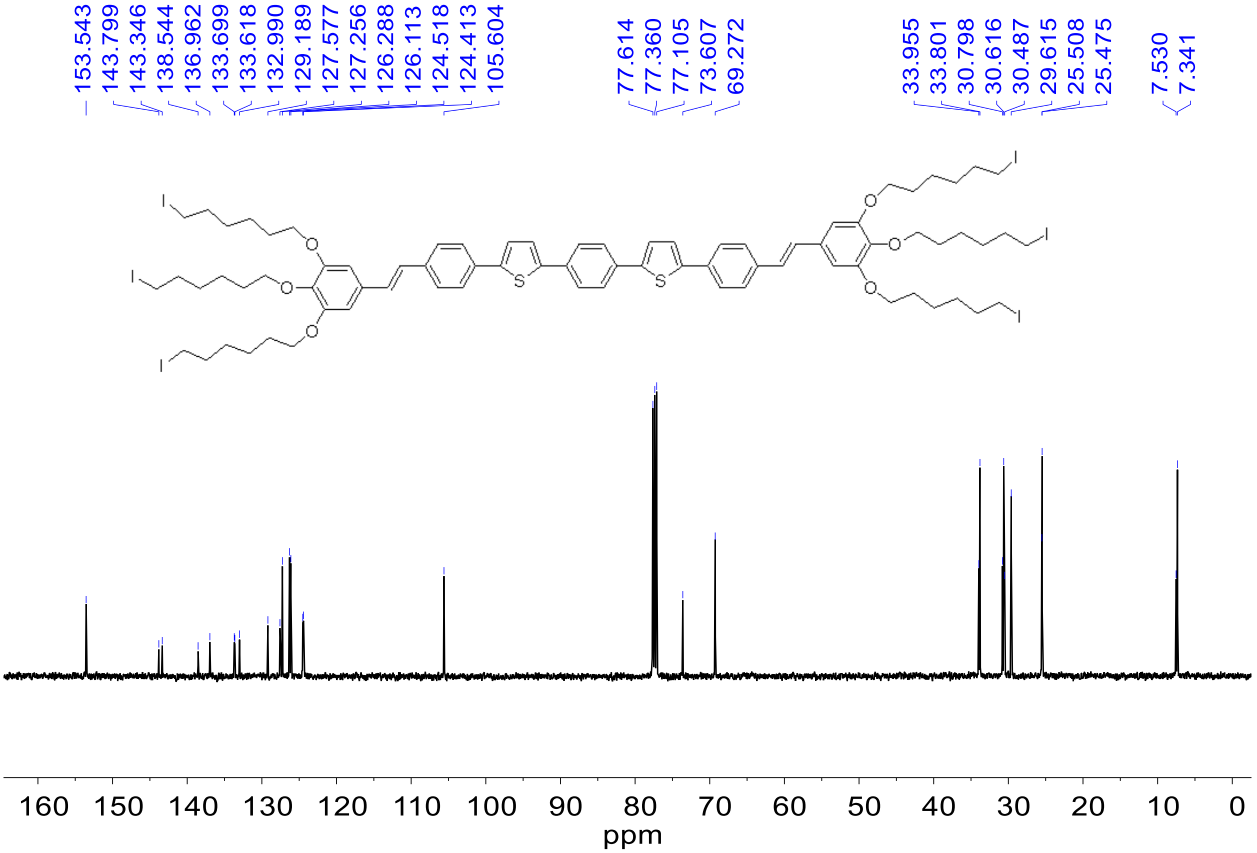

Fig. S83. Structure characterization by NMR. ^{13}C NMR spectrum of compound **21** in deuterated chloroform.

Compound **COE-Ben**:

To obtain this compound, compound **21** was used for a similar quaternization reaction as described above for compound **COE-BSe**. The product **COE-Ben** was afforded as a yellow solid (397 mg, 95 % yield). ^{1}H NMR (500 MHz, DMSO-d_6, 353K) δ 7.76 (s, 4H), 7.72 (d, *J* = 8.2 Hz, 4H), 7.65 (d, *J* = 8.4 Hz, 4H), 7.58 (d, *J* = 3.9 Hz, 2H), 7.56 (d, *J* = 3.9 Hz, 2H), 7.20 (s, 4H), 6.94 (s, 4H), 4.12 – 4.05 (m, 8H), 3.98 – 3.92 (m, 4H), 3.41 – 3.32 (m, 12H), 3.11 (s, 54H), 1.86 – 1.68 (m, 24H), 1.59 – 1.51 (m, 12H), 1.47 – 1.36 (m, 12H). ^{13}C NMR (126 MHz, DMSO, 353K) δ 153.27, 143.29, 142.62, 138.53, 137.34, 133.40, 133.03, 129.54, 127.60, 127.54, 126.38, 126.05, 125.71, 125.47, 106.54, 73.01, 69.29, 66.26, 30.02, 29.23, 26.19, 26.06, 25.55, 25.50, 22.68.

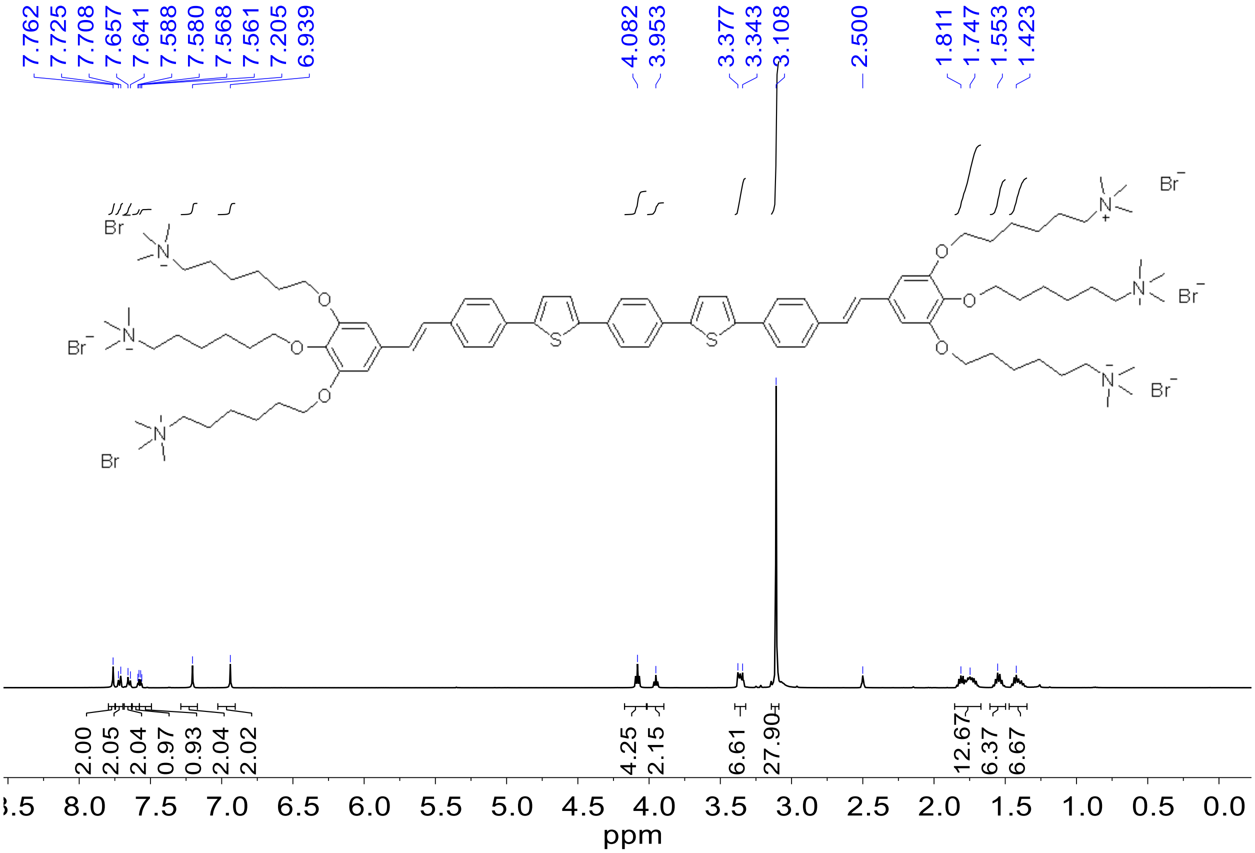

Fig. S84. Structure characterization by NMR. ^{1}H NMR spectrum of compound **COE-Ben** in deuterated DMSO.

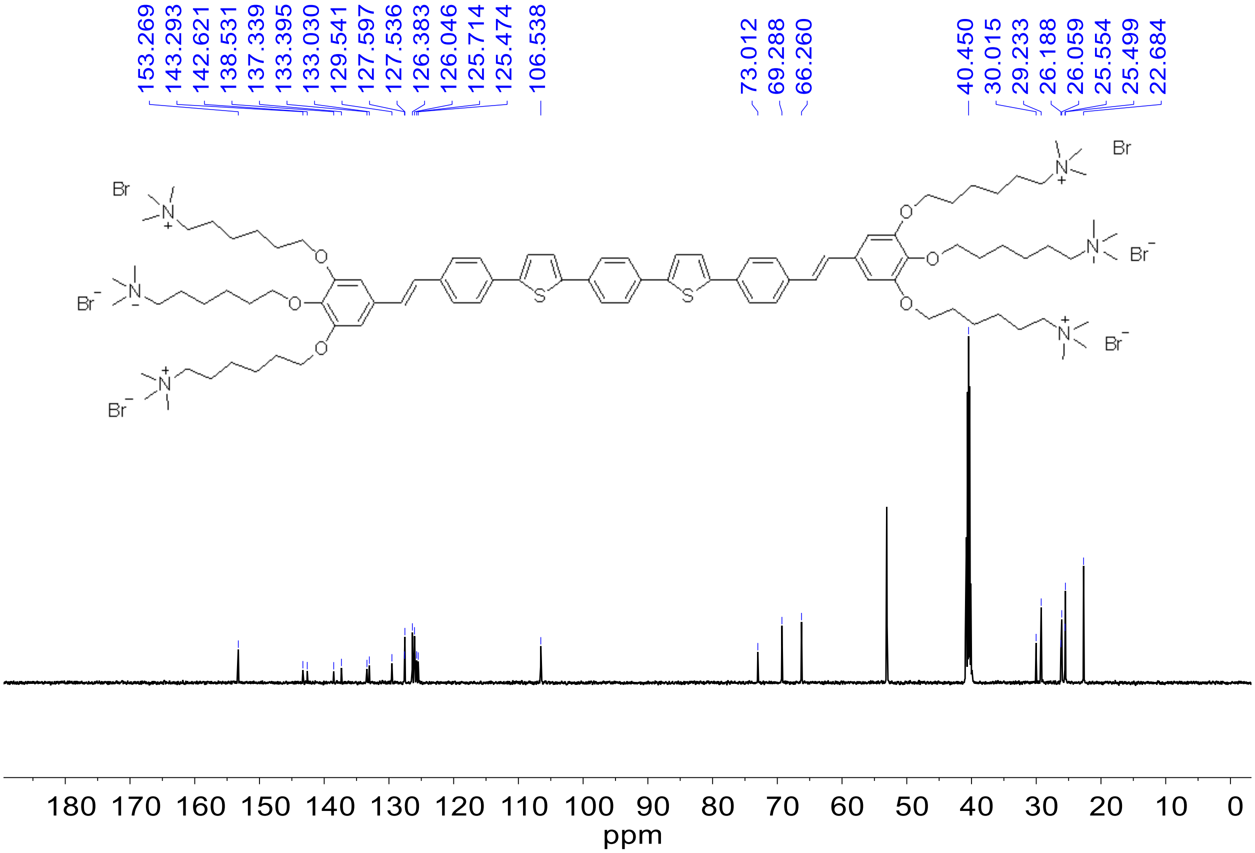

Fig. S85. Structure characterization by NMR. ^{13}C NMR spectrum of compound **COE-Ben** in deuterated DMSO.

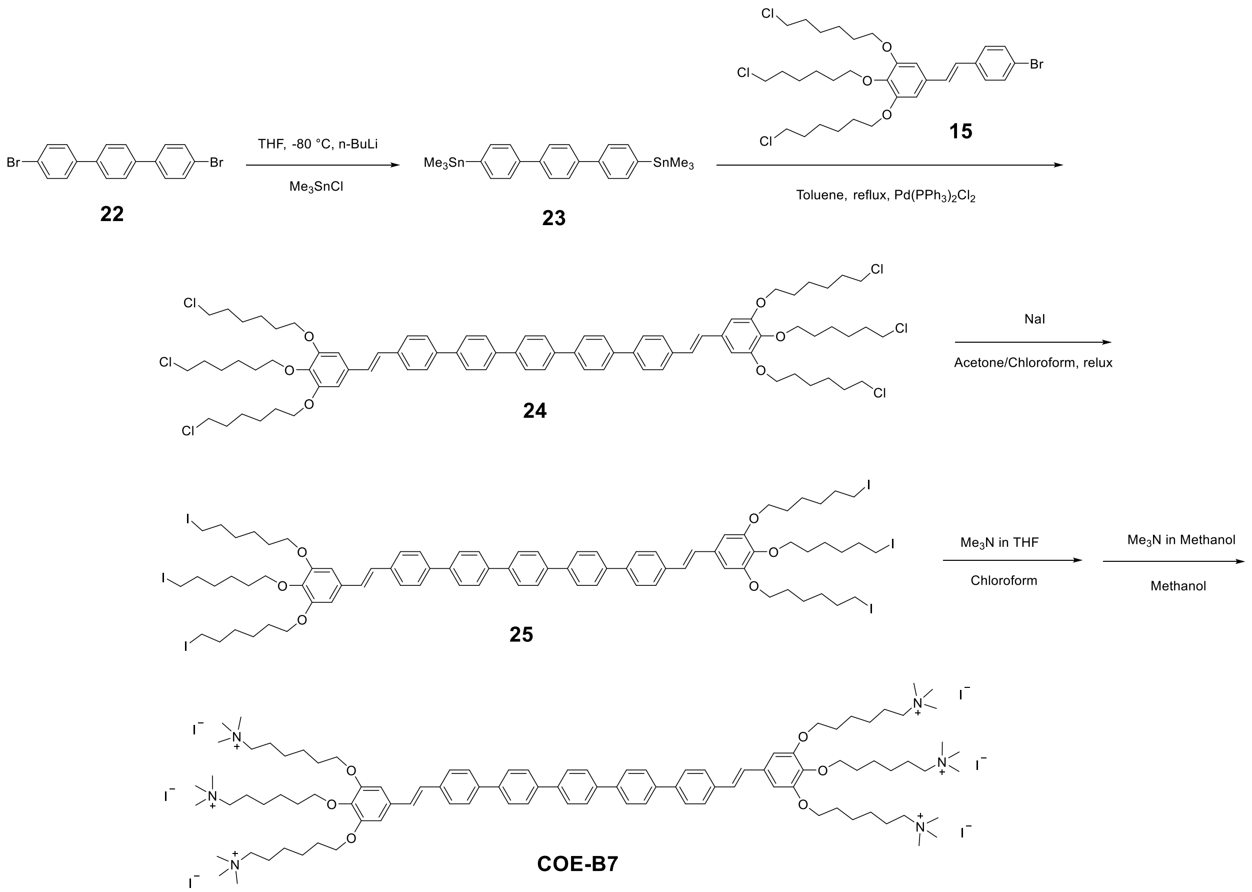

Fig. S86. Synthetic pathway. The synthetical route for **COE-B7**.

4,4''-Bis(trimethylstannyl)-1,1':4',1''-terphenyl (compound **23**):
4,4''-Dibromo-1,1':4',1''-terphenyl (compound **22**) (1.10 g, 2.82 mmol) was added to a flask and purged using argon, and then 150 mL dry THF were added under argon protection. The mixture was cooled down to –80 °C utilizing an ultralow temperature reaction bath. Then, 5.64 mL *n*-butyllithium solution in cyclohexane (2 M, 11.28 mmol) was added drop-wise into the reaction mixture. After stirring at -80 °C for 2 hours, the reaction mixture was warmed up to room temperature and stirred for 1 hour at room temperature. After that, the reaction mixture was cooled down to –80 °C utilizing an ultralow temperature reaction bath again. Then, 16.9 mL trimethyltin chloride solution in hexane (1 M, 16.9 mmol) was added and the reaction mixture was warmed up to room temperature. After being stirred at room temperature overnight (about 16 hours), the reaction mixture was concentrated using rotary evaporator and then poured into water and extracted with chloroform. The organic phase was washed using water and then dried over Na_2SO_4. After the solvent being removed using rotary evaporator, the crude product was purified via recrystallization using chloroform and methane as the solvent pair. The product was obtained by filtration and drying in vacuum as white solid (0.64, 41 % yield). ^{1}H NMR (500 MHz, Chloroform-

d, 298K) δ 7.69 – 7.67 (m, 4H), 7.65 – 7.58 (m, 8H), 0.34 (s, 18H). ^{13}C NMR (126 MHz, $CDCl_3$, 298K) δ 141.61, 140.96, 140.51, 136.67, 127.84, 126.99, -9.17.

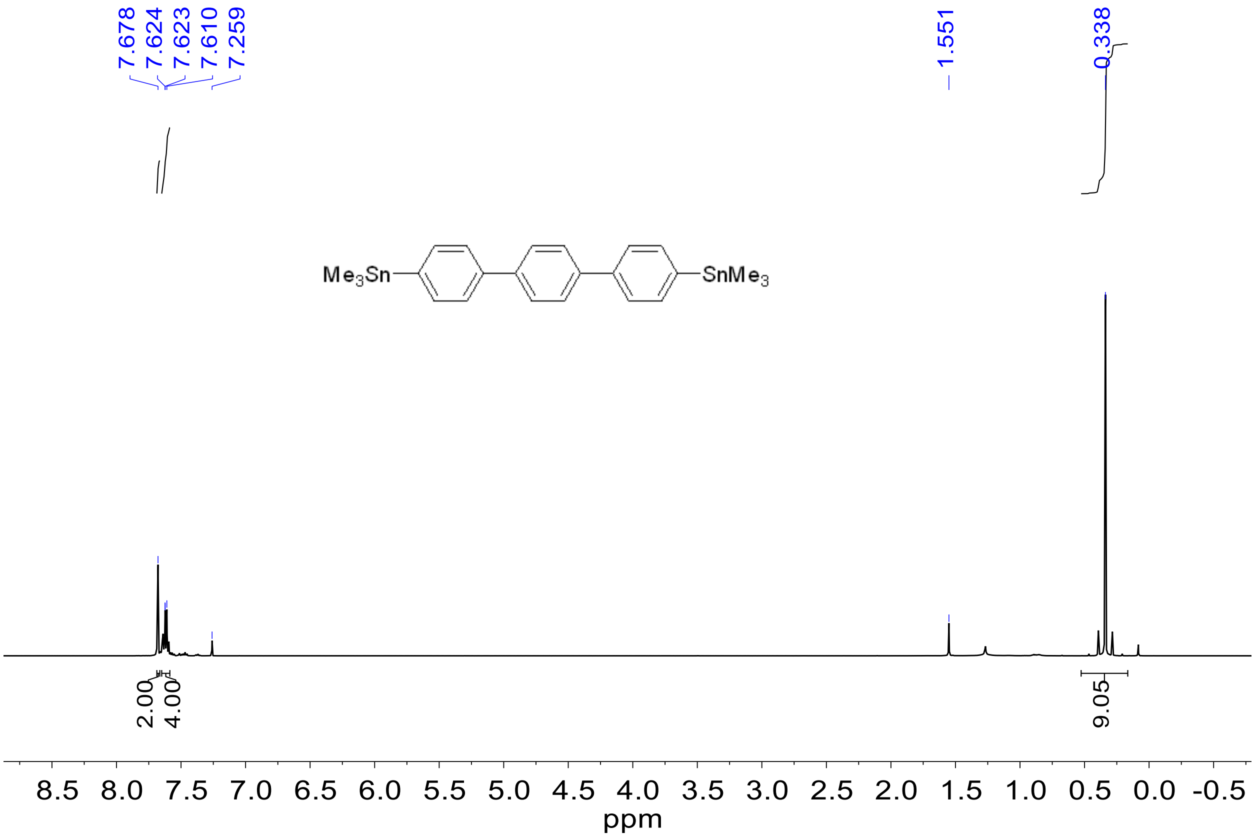

Fig. S87. Structure characterization by NMR. ^{1}H NMR spectrum of compound **23** in deuterated chloroform.

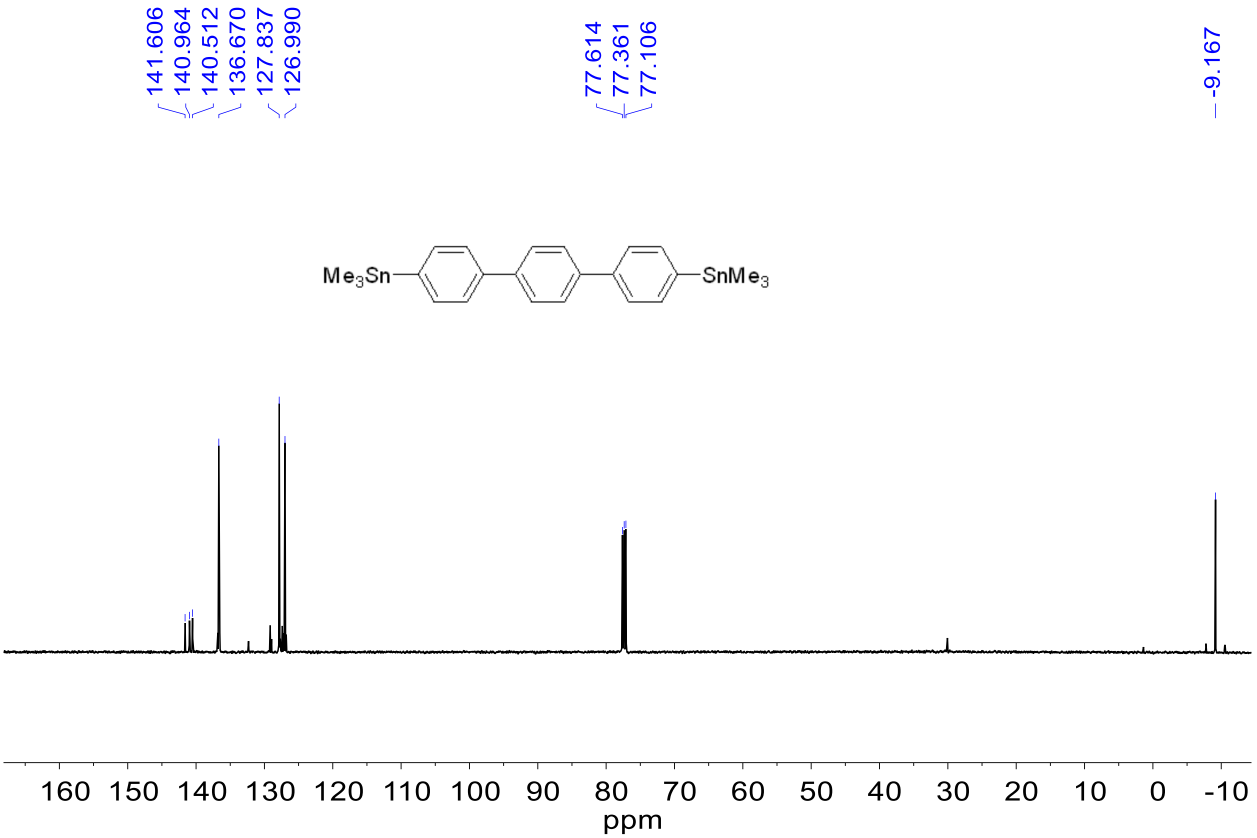

Fig. S88. Structure characterization by NMR. ^{13}C NMR spectrum of compound **23** in deuterated chloroform.

4,4''''-Bis((E)-3,4,5-tris((6-chlorohexyl)oxy)styryl)-1,1':4',1'':4'',1''':4''',1''''-quinquephenyl (compound **24**):

4,4''-Bis(trimethylstannyl)-1,1':4',1''-terphenyl (compound **23**) (0.271 g, 0.487 mmol), (*E*)-5-(4-bromostyryl)-1,2,3-tris((6-chlorohexyl)oxy)benzene (compound **15**) (0.969 g, 1.46 mmol), catalyst $Pd(PPh_3)_4$ (22.5 mg, 0.0195 mmol) were added to a round flask under the protection of argon atmosphere. After purged using argon, 20 mL dry toluene was added to the reaction mixture, which was then heated at 110 °C for 24 hours with stirring. After being cooled to room temperature, the reaction mixture was poured into water, and extracted with chloroform. The organic phase was dried over Na_2SO_4 and then the organic solvent was removed by rotary evaporator. The crude product was purified with silica gel column chromatography using hexane : dichloromethane = 1 : 10 as eluent. The product was dissolved in chloroform and precipitated using methanol, and the precipitation were collected by filtration and washed using methanol. After being dried in vacuum, the product was obtained as yellow solid (0.546 g, 80 % yield). ^{1}H NMR (500 MHz, Chloroform-*d*, 298K) δ 7.80 – 7.71 (m, 12H), 7.67 (d, *J* = 8.0 Hz, 4H), 7.60 (d, *J* = 8.1 Hz, 4H), 7.08 (d, *J* = 16.1 Hz, 2H), 7.03 (d, *J* = 16.2 Hz, 2H), 6.75 (s, 4H), 4.12 – 3.93 (m, 12H), 3.64 – 3.50 (m, 12H), 1.95 – 1.72 (m, 24H), 1.61 – 1.45 (m, 24H). ^{13}C NMR (126 MHz, $CDCl_3$, 298K) δ 153.57, 139.95, 139.90, 138.53, 136.85, 133.07, 129.22, 127.75, 127.71, 127.63, 127.56, 127.24, 105.60, 73.60, 69.27, 45.44, 45.35, 33.01, 32.91, 30.51, 29.65, 27.16, 27.00, 25.82.

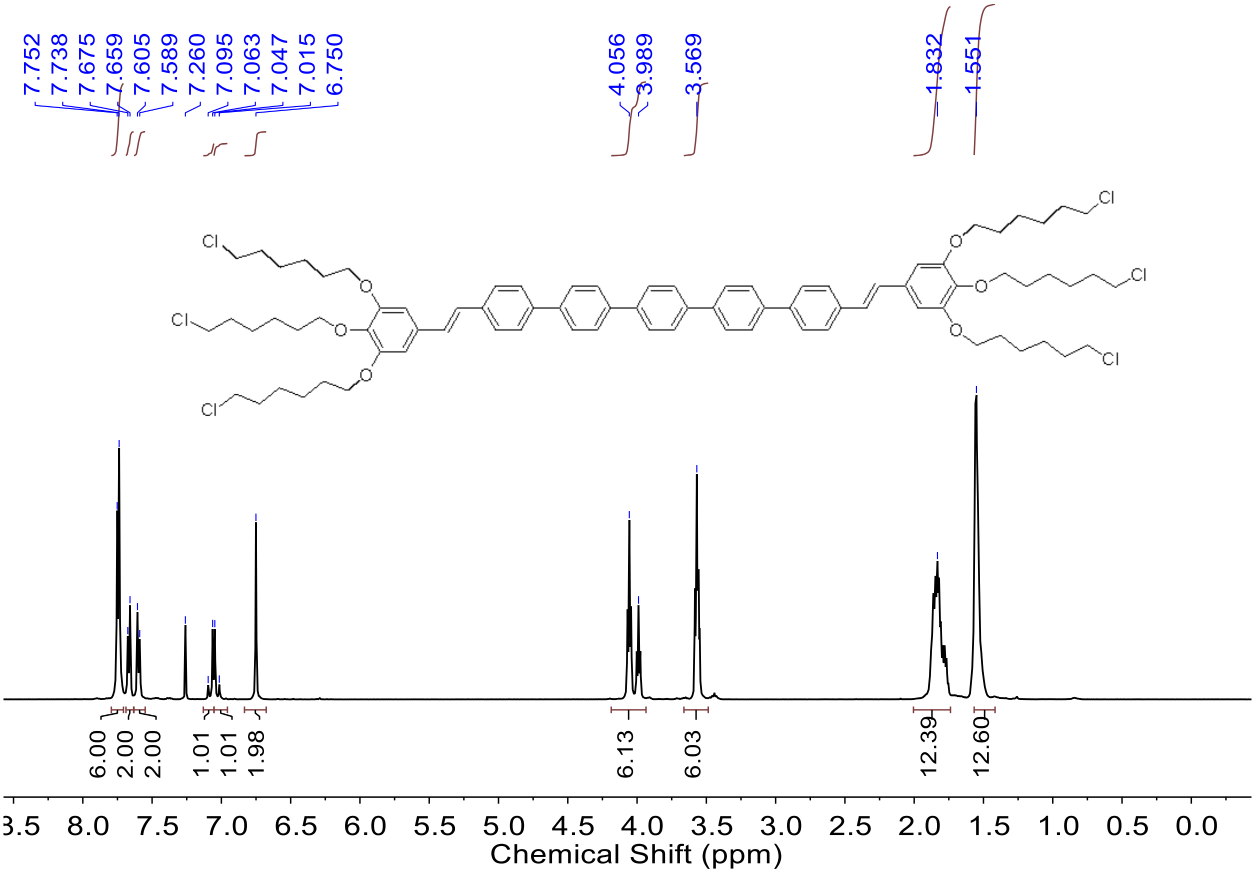

Fig. S89. Structure characterization by NMR. ^{1}H NMR spectrum of compound **24** in deuterated chloroform.

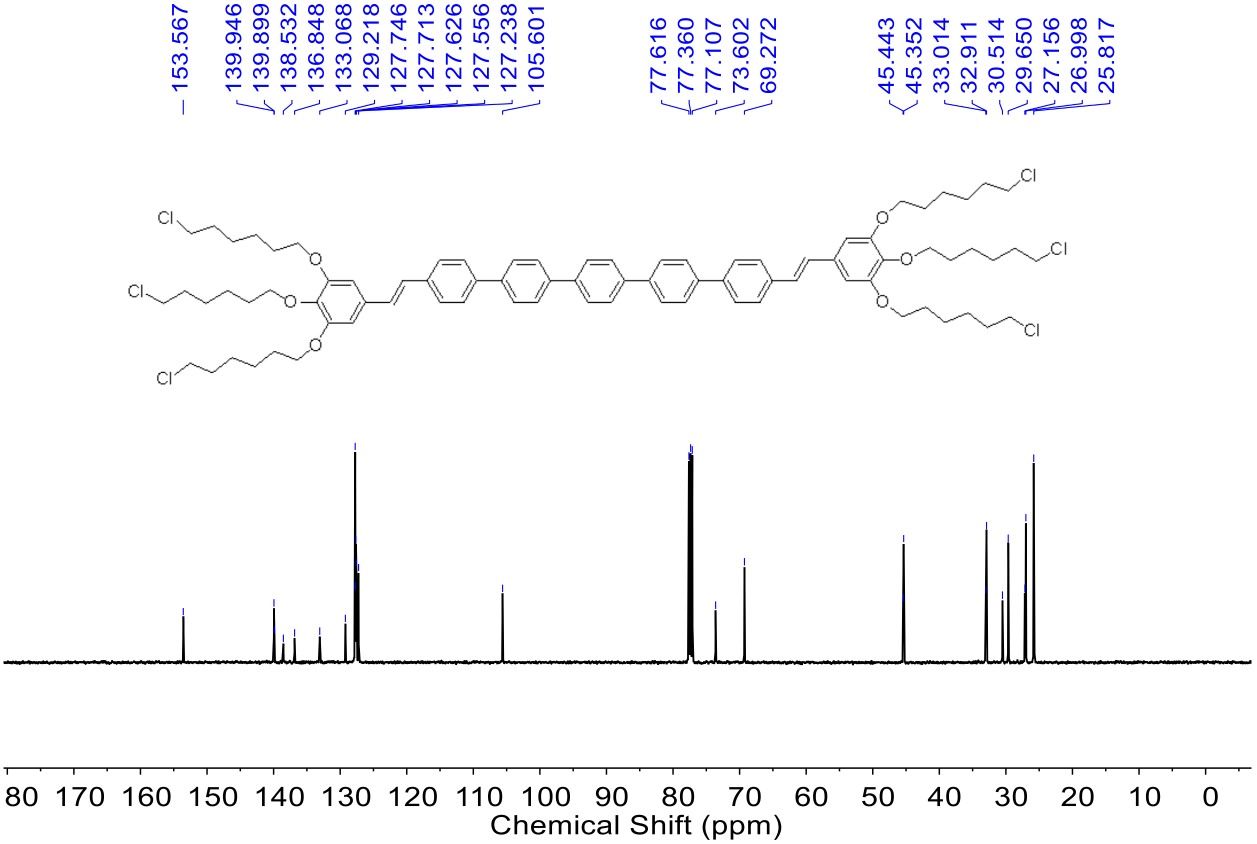

Fig. S90. Structure characterization by NMR. ^{13}C NMR spectrum of compound **24** in deuterated chloroform.

5,8-Bis(5-(4-((*E*)-3,4,5-tris((6-bromohexyl)oxy)styryl)phenyl)thiophen-2-yl)quinoxaline (compound **25**):

This compound was synthesized according to a similar procedure as described for compound **21** above, but the reactant 1,4-bis(5-(4-((*E*)-3,4,5-tris((6-chlorohexyl)oxy)styryl)phenyl)thiophen-2-yl)benzene (compound **20**) was replaced by 4,4''''-bis((*E*)-3,4,5-tris((6-chlorohexyl)oxy)styryl)-1,1':4',1'':4'',1''':4''',1''''-quinquephenyl (compound **24**). The final product was obtained as a yellow solid (390 mg, 88 % yield). ^{1}H NMR (500 MHz, Chloroform-*d*) δ 7.75 (d, *J* = 6.6 Hz, 12H), 7.67 (d, *J* = 8.1 Hz, 4H), 7.60 (d, *J* = 8.1 Hz, 4H), 7.06 (d, *J* = 16.1 Hz, 2H), 7.05 (d, *J* = 16.3Hz, 2H), 6.75 (s, 4H), 4.12 – 3.94 (m, 12H), 3.30 – 3.16 (m, 12H), 1.95 – 1.73 (m, 24H), 1.60 – 1.44 (m, 24H). ^{13}C NMR (126 MHz, $CDCl_3$, 298K) δ 153.55, 139.95, 138.50, 136.84, 133.06, 129.21, 127.75, 127.72, 127.63, 127.56, 127.24, 105.58, 73.61, 69.26, 33.95, 33.79, 30.80, 30.61, 30.48, 29.61, 25.50, 25.47, 7.55, 7.36.

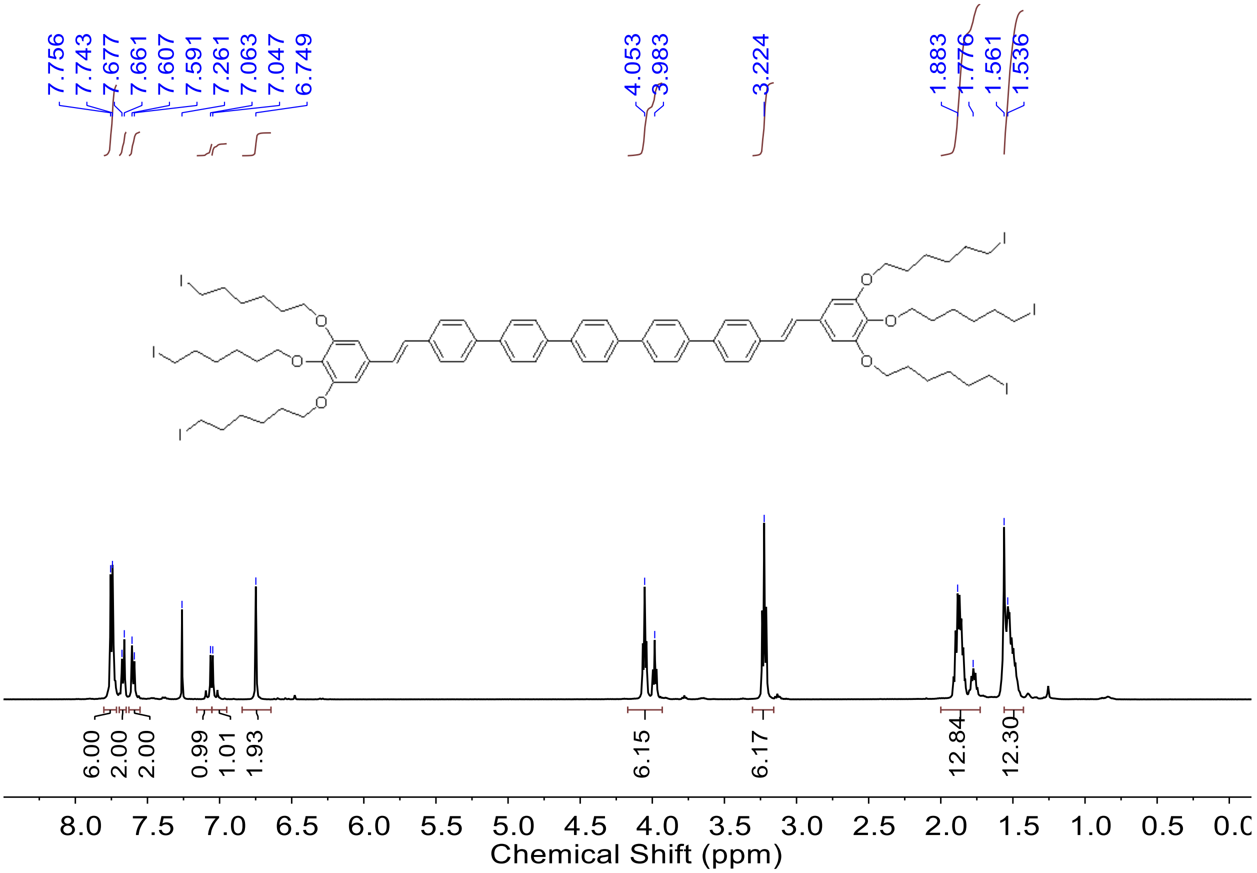

Fig. S91. Structure characterization by NMR. ^{1}H NMR spectrum of compound **25** in deuterated chloroform.

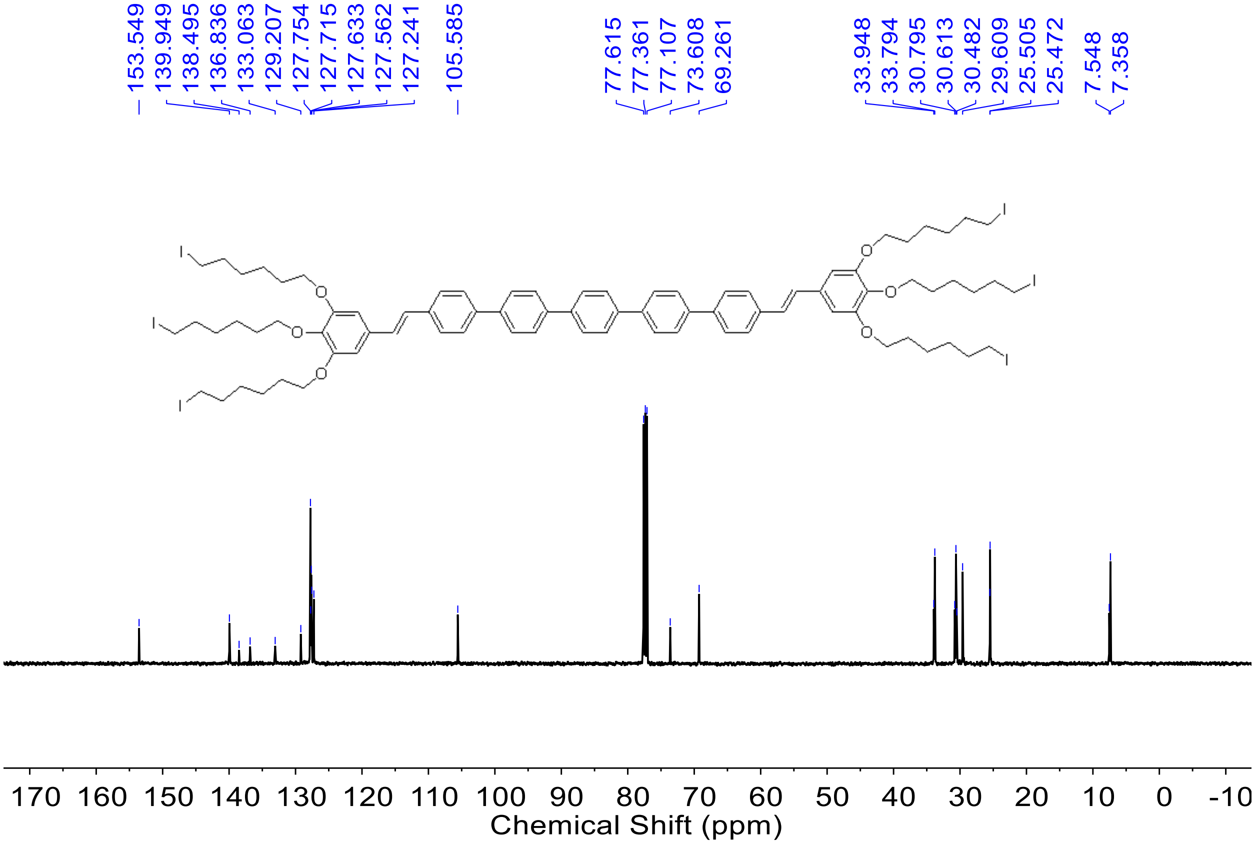

Fig. S92. Structure characterization by NMR. ^{13}C NMR spectrum of compound **25** in deuterated chloroform.

Compound **COE-B7**:

To obtain this compound, compound **25** was used for a similar quaternization reaction as described above for compound **COE-BSe**. The product **COE-B7** was afforded as a yellow solid (214 mg, 93 % yield). ^{1}H NMR (500 MHz, DMSO-d_6, 353K) δ 7.89 – 7.82 (m, 12H), 7.78 (d, J = 8.3 Hz, 4H), 7.70 (d, J = 8.3 Hz, 4H), 7.23 (s, 4H), 6.95 (s, 4H), 4.12 – 4.05 (m, 8H), 3.98 – 3.92 (m, 4H), 3.39 – 3.31 (m, 12H), 3.11 (s, 54H), 1.87 – 1.68 (m, 24H), 1.60 – 1.50 (m, 12H), 1.47 – 1.36 (m, 12H). ^{13}C NMR (126 MHz, DMSO, 353K) δ 153.30, 139.32, 139.26, 139.18, 139.05, 138.52, 137.14, 133.11, 129.47, 127.76, 127.57, 127.46, 127.27, 106.55, 73.02, 69.31, 66.27, 53.13, 53.10, 53.07, 30.02, 29.25, 26.20, 26.07, 25.56, 25.51, 22.69.

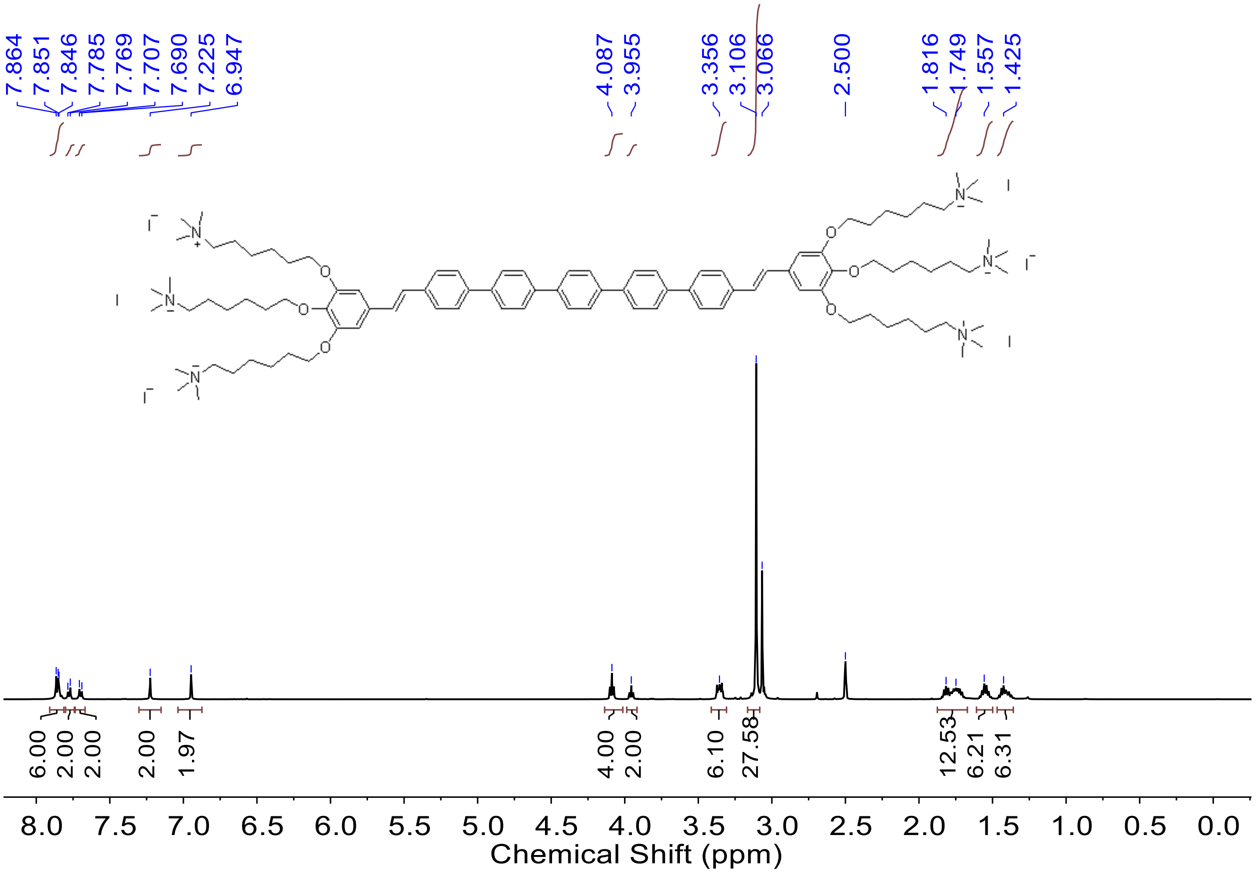

Fig. S93. Structure characterization by NMR. ^{1}H NMR spectrum of compound **COE-B7** in deuterated DMSO.

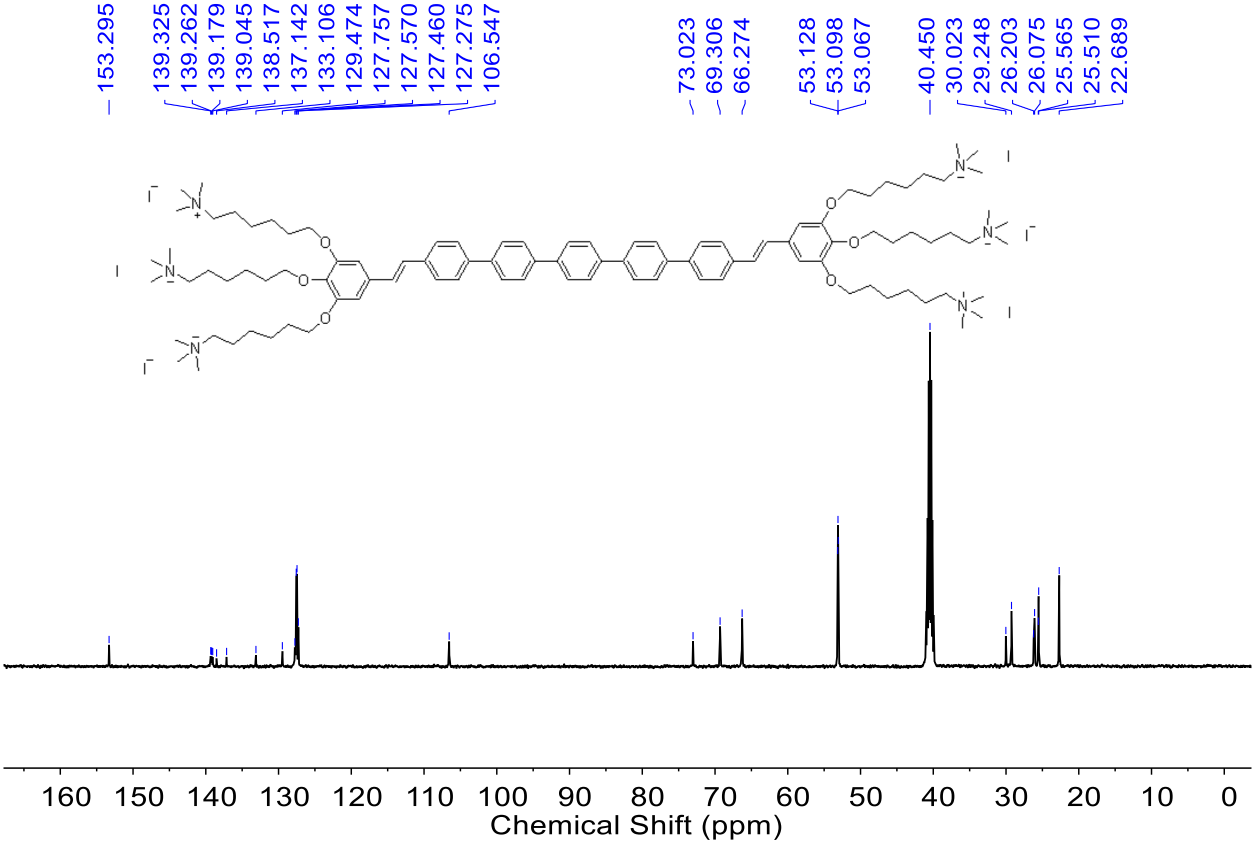

Fig. S94. Structure characterization by NMR. ^{13}C NMR spectrum of compound **COE-B7** in deuterated DMSO.
